# *APOE* Genotype and Alzheimer Disease Risk Across Age, Sex, and Population Ancestry

**DOI:** 10.1001/jamaneurol.2023.3599

**Published:** 2023-11-06

**Authors:** Michael E. Belloy, Shea J. Andrews, Yann Le Guen, Michael Cuccaro, Lindsay A. Farrer, Valerio Napolioni, Michael D. Greicius

**Affiliations:** 1 Department of Neurology and Neurological Sciences, Stanford University, Stanford, California; 2 NeuroGenomics and Informatics Center, Washington University School of Medicine, St. Louis, Missouri; 3 Department of Neurology, Washington University School of Medicine, St. Louis, Missouri; 4 Department of Psychiatry and Behavioral Sciences, University of California, San Francisco; 5 John P. Hussman Institute for Human Genomics, University of Miami Miller School of Medicine, Miami, Florida; 6 Dr. John T. Macdonald Foundation, Department of Human Genetics, University of Miami Miller School of Medicine, Miami, Florida; 7 Department of Medicine, Biomedical Genetics, Boston University Chobanian & Avedisian School of Medicine, Boston, Massachusetts; 8 Department of Neurology, Boston University Chobanian & Avedisian School of Medicine, Boston, Massachusetts; 9 Department of Ophthalmology, Boston University Chobanian & Avedisian School of Medicine, Boston, Massachusetts; 10 Department of Biostatistics, Boston University School of Public Health, Boston, Massachusetts; 11 Department of Epidemiology, Boston University School of Public Health, Boston, Massachusetts; 12 School of Biosciences and Veterinary Medicine, University of Camerino, Camerino, Italy

## Abstract

**Importance:**

Apolipoprotein E (*APOE*)*2 and APOE*4 are, respectively, the strongest protective and risk-increasing, common genetic variants for late-onset Alzheimer disease (AD), making *APOE* status highly relevant toward clinical trial design and AD research broadly. The associations of *APOE* genotypes with AD are modulated by age, sex, race and ethnicity, and ancestry, but these associations remain unclear, particularly among racial and ethnic groups understudied in the AD and genetics research fields.

**Objective:**

To assess the stratified associations of *APOE* genotypes with AD risk across sex, age, race and ethnicity, and global population ancestry.

**Design, Setting, Participants:**

This genetic association study included case-control, family-based, population-based, and longitudinal AD-related cohorts that recruited referred and volunteer participants. Data were analyzed between March 2022 and April 2023. Genetic data were available from high-density, single-nucleotide variant microarrays, exome microarrays, and whole-exome and whole-genome sequencing. Summary statistics were ascertained from published AD genetic studies.

**Main Outcomes and Measures:**

The main outcomes were risk for AD (odds ratios [ORs]) and risk of conversion to AD (hazard ratios [HRs]), with 95% CIs. Risk for AD was evaluated through case-control logistic regression analyses. Risk of conversion to AD was evaluated through Cox proportional hazards regression survival analyses.

**Results:**

Among 68 756 unique individuals, analyses included 21 852 East Asian (demographic data not available), 5738 Hispanic (68.2% female; mean [SD] age, 75.4 [8.8] years), 7145 non-Hispanic Black (hereafter referred to as Black) (70.8% female; mean [SD] age, 78.4 [8.2] years), and 34 021 non-Hispanic White (hereafter referred to as White) (59.3% female; mean [SD] age, 77.0 [9.1] years) individuals. There was a general, stepwise pattern of ORs for APOE*4 genotypes and AD risk across race and ethnicity groups. Odds ratios for APOE*34 and AD risk attenuated following East Asian (OR, 4.54; 95% CI, 3.99-5.17),White (OR, 3.46; 95% CI, 3.27-3.65), Black (OR, 2.18; 95% CI, 1.90-2.49) and Hispanic (OR, 1.90; 95% CI, 1.65-2.18) individuals. Similarly, ORs for APOE*22+23 and AD risk attenuated following White (OR, 0.53, 95% CI, 0.48-0.58), Black (OR, 0.69, 95% CI, 0.57-0.84), and Hispanic (OR, 0.89; 95% CI, 0.72-1.10) individuals, with no association for Hispanic individuals. Deviating from the global pattern of ORs, APOE*22+23 was not associated with AD risk in East Asian individuals (OR, 0.97; 95% CI, 0.77-1.23). Global population ancestry could not explain why Hispanic individuals showed APOE associations with less pronounced AD risk compared with Black and White individuals. Within Black individuals, decreased global African ancestry or increased global European ancestry showed a pattern of APOE*4 dosage associated with increasing AD risk, but no such pattern was apparent for APOE*2 dosage with AD risk. The sex-by-age–specific interaction effect of APOE*34 among White individuals (higher risk in women) was reproduced but shifted to ages 60 to 70 years (OR, 1.48; 95% CI, 1.10-2.01) and was additionally replicated in a meta-analysis of Black individuals and Hispanic individuals (OR, 1.72; 95% CI, 1.01-2.94).

**Conclusion and Relevance:**

Through recent advances in AD-related genetic cohorts, this study provided the largest-to-date overview of the association of *APOE* with AD risk across age, sex, race and ethnicity, and population ancestry. These novel insights are critical to guide AD clinical trial design and research.

## Key Points

**Question:**

How do associations of apolipoprotein E (*APOE*) genotypes with late-onset Alzheimer disease (AD) risk differ across age, sex, and population ancestry?

**Findings:**

In this genetic association study of 68 756 unique individuals, there was a stepwise pattern of decreasing effect estimates for APOE*4 following East Asian, non-Hispanic White, non-Hispanic Black, and Hispanic individuals. There was a similar stepwise pattern of increasing effect estimates for APOE*2 following non-Hispanic White, non-Hispanic Black, and Hispanic individuals, with no association for APOE*2 in East Asian individuals and Hispanic individuals.

**Meaning:**

This study found associations of *APOE* with AD risk across important biologic and demographic strata, which may guide AD clinical trial design and research.

## Teaser

This genetic association study assesses associations between apolipoprotein E and Alzheimer disease risk across age, sex, race and ethnicity, and global population ancestry.

## Introduction

Apolipoprotein E (*APOE*)*2 and APOE*4 are, respectively, the strongest protective and risk-increasing, common genetic variants for late-onset Alzheimer disease (AD), making an individual’s *APOE* status highly relevant toward clinical trial design and AD research broadly.^1,2^ Importantly, associations of *APOE* genotypes with AD are modulated by age, sex, race and ethnicity, and population ancestry, but these associations remain unclear, particularly in racial and ethnic groups historically understudied in the AD and genetics research fields.^1,3,4,5,6^ While the field has advanced insights into this matter across the past decade, a 1997 landmark study by Farrer et al^3^ published in *JAMA* remains a common reference given its comprehensive assessment of the associations of *APOE* with AD risk across East Asian, Hispanic, non-Hispanic Black (hereafter referred to as Black), and non-Hispanic White (hereafter referred to as White) individuals as well as having launched initial insights into the age-specific sex dimorphism of the association of APOE*4 with AD risk (which was later replicated for APOE*34 in a more narrow age window).^3,7^ The sample sizes for Black, East Asian, and Hispanic individuals in the Farrer et al^3^ study were small, however, leaving many questions on the associations of *APOE* genotypes with AD risk in these racial and ethnic groups.

In parallel, there has been substantial evolution and debate regarding the appropriate use of race and ethnicity and genetic ancestry in biomedical research and clinical practice. Importantly, race and ethnicity are socially ascribed identities that capture risk related to epidemiologic factors and social determinants of health, while genetic ancestry relates to geographical origins and inherent biologic variation.^8,9^ Genetic population ancestry may be particularly relevant in identifying genetic variants with variable allele frequencies and effects across ancestry populations, which in turn could help explain heterogeneity in the associations of *APOE* with AD risk.^10,11,12^ Importantly, while race and ethnicity correlate with genetic ancestry (eg, a mixture of African, Amerindian, and European among Hispanic individuals; more African ancestry among Black individuals; and more European ancestry among White individuals),^13^ they are less accurate identifiers of genetic risk for disease.^9^ As such, it is relevant to study the association of *APOE* with AD risk considering both race and ethnicity and population ancestry as well as whether and how they interact to affect AD risk.

Through substantial advances in AD genetics, including the addition of various novel cohorts and increased efforts from the Alzheimer’s Disease Genetics Consortium and the Alzheimer’s Disease Sequencing Project to increase sample diversity, we now have access to publicly available data sets with substantially larger sample sizes for Black, Hispanic, and White individuals.^10,12,14,15,16^ Furthermore, given that these samples underwent array-based or sequencing-based genotyping, we can also perform extensive genetic and phenotypic data harmonization, evaluate effects of global population ancestry, and apply state-of-the-art quality control for more robust *APOE* genotyping.^17,18,19^ In parallel, other efforts in the field have led to the construction of genetic cohorts among East Asian individuals, for which summary statistics are available.^20^ Considering these important advances, we sought to reassess, in the largest such study to date, the association of *APOE* genotype with AD risk across important demographic and biologic variables that are known to interact with *APOE*. We specifically made use of racial and ethnic labels available in the considered genetic cohorts (following the National Institutes of Health definitions) and considered stratifications consistent with the 1997 Farrer et al study.^3^ We additionally evaluated the effects of stratifying by global population ancestry proportions.

## Methods

An in-depth overview of all methods is provided in the eMethods in Supplement 1. The current genetic association study followed the Strengthening the Reporting of Genetic Association Studies (STREGA) reporting guideline. Participants or their caregivers provided written informed consent in the original studies. The current study protocol was granted an exemption by the Stanford University institutional review board because the analyses were carried out on deidentified, “off-the-shelf” data; therefore, additional informed consent was not required.

### Ascertainment of Genotype and Phenotype Data

Case-control, family-based, and longitudinal AD-related genetic cohorts were available through public repositories, with genetic data from high-density single-nucleotide variant microarrays, exome microarrays, whole-exome sequencing, and whole-genome sequencing (eTables 1 and 2 in Supplement 1). Associations of *APOE* genotypes with AD risk among East Asian individuals were obtained through meta-analysis of 2 prior meta-analyses.^3,20^

### Ascertainment of Race and Ethnicity Data

Race and ethnicity were self-reported by study participants for which genetic data were directly available. Specifically, categories were defined by the National Institutes of Health. Race categories included American Indian or Alaska Native, Asian, Black or African American, Native Hawaiian or Other Pacific Islander, and White. Ethnicity categories included Hispanic or Latino or not Hispanic or Latino. If individuals did not identify with these racial and ethnic categories, they could report “other.” To increase sample size, for a subset of individuals, race information was inferred through a combination of cohort descriptive information and global population ancestry (eMethods in Supplement 1). Overall, the primary combined racial and ethnic groups available in the genetic samples were Black, Hispanic (including all race categories and other race), and White groups. Results for East Asian individuals were based on external data that included participants from Japan and Korea.

### Ancestry Determination, Quality Control, and Sample Processing

The design of sample processing is shown in eFigure 1 in Supplement 1. Genetic data underwent extensive quality control, imputation to the Trans-Omics for Precision Medicine reference panel (array-based samples),^21,22^ and ancestry determination (SNPweights, version 2.1 [Harvard T.H. Chan School of Public Health]) (eFigure 2 in Supplement 1).^23^ Global (ie, genome-wide) ancestry was determined with populations from the 1000 Genomes Project Consortium as a reference.^24^ By applying an ancestry percentage cutoff of 75% or greater, samples were stratified into the 5 super populations: African, Amerindian, East Asian, European, and South Asian. Participant relatedness was estimated from identity-by-descent analysis. Duplicate individuals were identified, and their clinical, diagnostic, and pathological data as well as age at onset of cognitive symptoms, age at examination for clinical diagnosis, age at last examination, age at death, sex, race, ethnicity, and *APOE* genotype were cross-referenced across cohorts. Duplicate entries with irreconcilable phenotypes were excluded. *APOE* genotypes were adjudicated using state-of-the-art *APOE* prioritization approaches, filtering out samples in which *APOE* genotypes lacked robustness (prioritizing *APOE* genotypes from sequencing data and cross-referencing *APOE* genotypes from high-quality imputation with those provided in study demographics through various protein-based and DNA-based methods).^17^ Finally, samples were filtered to age older than 55 years, cases or controls, belonging to 1 of 3 racial and ethnic groups available (Black, Hispanic, or White), and having no first-degree relatives included in any of the data sets. Inclusion of related individuals for modeling with mixed models was not pursued given that genetic relationship matrices in the current pooled analysis design would have variable accuracy due to various genetic sources. Final sample demographics and cohort or platform distributions are given in eTables 3 to 5 in Supplement 1.

### Statistical Analysis

In primary analyses, case-control logistic regressions (based on status at last visit) evaluated the associations of AD risk with APOE*2 dosage, APOE*4 dosage, or *APOE* genotype in each case with APOE*33 as the reference. APOE*2 dosage effect estimates were evaluated using the subset of APOE*2 allele carriers and APOE*33 individuals while adjusting for APOE*4 dosage (the inverse holds for APOE*4 dosage effects). *APOE* genotype effect estimates were evaluated 1 at a time, with subsetting of data to carriers of the *APOE* genotype of interest and individuals with APOE*33. Models adjusted for sex, cohort and platform (eTable 4 in Supplement 1), and global African, Amerindian, and European ancestry. These 3 primary ancestry covariates were included as there was minor variation from East Asian and South Asian ancestries, and the use of 3 compared with 2 ancestry covariates in the models did not lead to any overfitting or differing effect estimates. Multiple stratified designs were evaluated. *APOE*-by-sex associations were estimated through formal interaction analyses. *APOE*-by-race and ethnicity and *APOE*-by-ancestry associations were estimated through heterogeneity tests. Secondary survival analyses evaluated Cox proportional hazards regression for AD age at onset. Significant discoveries were considered at 2-sided *P* < .05. Age-stratified analyses used a sliding-window approach (10-year windows, 5-year overlap); thus, significant age-stratified discoveries were considered after Bonferroni correction for the number of nonoverlapping windows (*P* < .05; 4 = 0.0125). If windows included less than 100 individuals, associations were not evaluated, and if 95% CIs were excessively large, results were not visualized. If *APOE*-by-sex associations in 1 race and ethnicity or population ancestry group reached significance, we performed meta-analyses in other relevant groups and considered replication at 2-sided *P* < .05. Sensitivity analyses were conducted to evaluate the association of population ancestry proportion within racial and ethnic groups or regardless thereof, and to evaluate the impact of pathology verification status, ascertainment design, and excluding samples in which race status had to be inferred rather than being directly provided through demographic files. Data were analyzed between March 2022 and April 2023. All statistical analyses were conducted using R, version 4.2.1 (R Project for Statistical Computing).

## Results

### Race and Ethnicity Analyses

**Figure 1.**
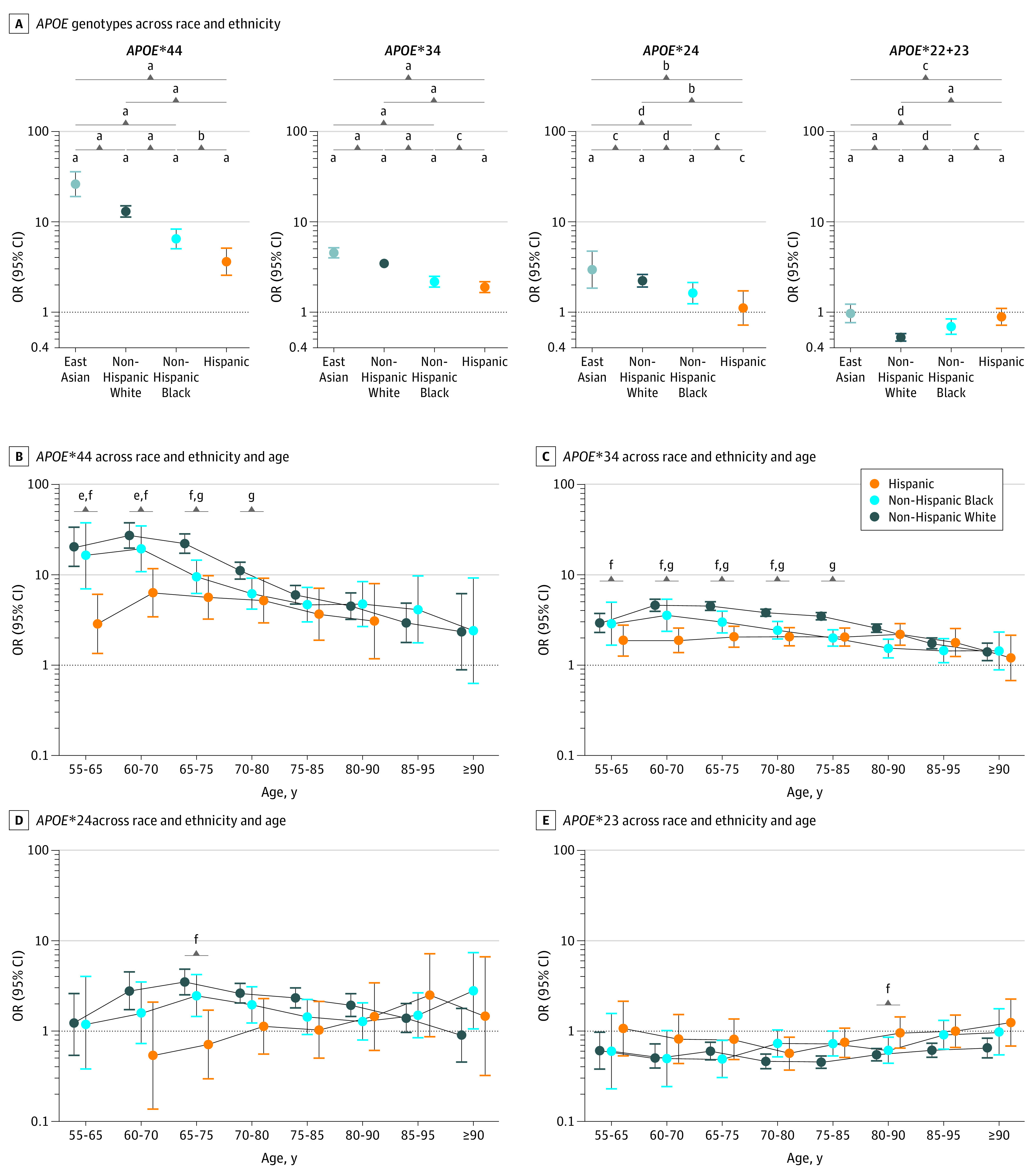
Associations of Apolipoprotein E (*APOE*) Genotypes With Alzheimer Disease Risk Across Race and Ethnicity and Age A, Related summary statistics are given in eTable 6 in Supplement 1. ^a^*P* < .001. ^b^*P* < .01. ^c^*P* ≥ .05. ^d^*P* < .05. ^e^*P* < .05 for Hispanic individuals vs non-Hispanic Black individuals after Bonferroni correction of the amount of overlapping age windows. ^f^*P* < .05 for Hispanic individuals vs non-Hispanic White individuals after Bonferroni correction of the amount of overlapping age windows. ^g^*P* < .05 for non-Hispanic Black individuals vs non-Hispanic White individuals after Bonferroni correction of the amount of overlapping age windows.

Of 68 756 unique individuals available for association analyses, 7145 were Black (70.8% female; mean [SD] age, 78.4 [8.2] years); 21 852, East Asian (demographic data not available); 5738, Hispanic (68.2% female; mean [SD] age, 75.4 [8.8] years); and 34 021, White (59.3% female; mean [SD] age, 77.0 [9.1] years) (eFigure 1 and eTable 3 in Supplement 1). Assessment of *APOE* genotypes among East Asian individuals was limited to results reported in prior studies.^3,20^ Results from all primary analyses across racial and ethnic groups are given in Figure 1, Table, and eFigures 3 and 4 and eTable 6 in Supplement 1. Considerable race and ethnicity differences were observed across age and non–age-stratified analyses. There was a general, stepwise pattern of ORs for APOE*4 genotypes and AD risk across race and ethnicity groups. Odds ratios for APOE*34 and AD risk attenuated following East Asian (OR, 4.54; 95% CI, 3.99-5.17), White (OR, 3.46; 95% CI, 3.27-3.65), Black (OR, 2.18; 95% CI, 1.90-2.49), and Hispanic (OR, 1.90; 95% CI, 1.65-2.18) individuals. Similarly, ORs for APOE*22+23 and AD risk attenuated following White (OR, 0.53, 95% CI, 0.48-0.58), Black (OR, 0.69, 95% CI, 0.57-0.84), and Hispanic (OR, 0.89; 95% CI, 0.72-1.10) individuals, with no association for Hispanic individuals. Deviating from the global pattern of ORs, APOE*22+23 was not associated with AD risk in East Asian individuals (OR, 0.97; 95% CI, 0.77-1.23). Notably, the OR for APOE*4 genotypes and dosage was lowest in Hispanic individuals compared with other race and ethnicity groups. Similarly, there was no association of APOE*2 with AD risk in Hispanic individuals (Table). Sensitivity analyses indicated that this was not due to *APOE* associations differing across Black or White race (eTable 7 in the Supplement 1). More White individuals had pathology-verified diagnoses, which may have caused bias in cross–race and ethnicity comparisons (eTable 3 in Supplement 1). Sensitivity analyses using only clinically determined diagnoses or adjusting for pathology verification status, however, suggested that results were consistent (eTables 8 and 9 in Supplement 1). Similarly, different cohort ascertainment design proportions across racial and ethnic groups may bias results,^3^ but related sensitivity analyses showed consistent findings (eTables 10 and 11 in Supplement 1). It was notable that in non–community-based samples across Black and White groups, there was similar AD risk associated with APOE*23 (slightly more protective in White individuals), and in Black individuals, APOE*34 and APOE*44 outcome estimates showed greater AD risk compared with what was observed in primary analyses. Furthermore, sensitivity analyses excluding samples in which race information had to be inferred showed no notable differences compared with primary analyses except for potentially a slightly lower AD risk associated with APOE*4 among White individuals (eTable 12 in Supplement 1).

**Table.** *APOE* Dosage and Genotype Associations With Alzheimer Disease Risk Across Racial and Ethnic Groups and Sex^a^

	White individuals (n = 34 021)			Black individuals (n = 7145)			Hispanic individuals (n = 5738)				*P* value				
	Carriers, No.	OR (95% CI)	*P* value	Carriers, No.	OR (95% CI)	*P* value	Carriers, No.	OR (95% CI)		*P* value	Black vs White		Hispanic vs White		Hispanic vs Black
***APOE* across race and ethnicity**															
APOE*4 and APOE*2 dosage															
APOE*2 dosage	3760	0.55 (0.50-0.60)	1.07E−39^b^	1411	0.72 (0.60-0.86)	4.14E−04^b^	607	0.90 (0.74-1.10)		.32	8.57E−03^b^		8.12E−06^b^		.10
APOE*33	17 199	1 [Reference]	NA	3095	1 [Reference]	NA	3517	1 [Reference]		NA	NA		NA		NA
APOE*4 dosage	13 803	3.48 (3.32-3.64)	<1.0E−300^b^	2973	2.39 (2.16-2.65)	3.74E−62^b^	1715	1.90 (1.70-2.13)		2.46E−28^b^	8.08E−11^b^		7.63E−22^b^		3.36E−03^b^
*APOE* genotype															
APOE*22	133	0.39 (0.25-0.61)	3.33E−05^b^	65	0.78 (0.38-1.58)	.49	24	1.02 (0.41-2.51)		.97	.11		.06		.65
APOE*23	2886	0.53 (0.48-0.59)	1.86E−37^b^	1012	0.69 (0.56-0.84)	2.79E−04^b^	482	0.89 (0.71-1.1)		.28	.03^b^		3.43E−05^b^		.10
APOE*33	17 199	1 [Reference]	NA	3095	1 [Reference]	NA	3517	1 [Reference]		NA	NA		NA		NA
APOE*24	741	2.23 (1.90-2.62)	6.39E−23^b^	334	1.63 (1.24-2.13)	4.06E−04^b^	101	1.11 (0.72-1.72)		.63	.046^b^		3.43E−03^b^		.15
APOE*34	10 807	3.46 (3.27-3.65)	<1.0E−300^b^	2233	2.18 (1.90-2.49)	9.82E−30^b^	1426	1.90 (1.65-2.18)		8.39E−20^b^	4.82E−10^b^		2.44E−15^b^		.16
APOE*44	2255	13.04 (11.31-15.04)	1.16E−273^b^	406	6.49 (5.07-8.31)	1.10E−49^b^	188	3.62 (2.56-5.11)		2.55E−13^b^	1.64E−06^b^		1.63E−11^b^		7.01E−03^b^
***APOE* across race and ethnicity and sex**															
APOE*4 and APOE*2 dosage															
APOE*2 dosage, female	2277	0.51 (0.45-0.57)	1.81E−29^b^	970	0.76 (0.61-0.94)	.01^b^	430	0.98 (0.77-1.24)		.85	1.47E−03^b^		1.16E−06^b^		.12
APOE*33, female	10 274	1 [Reference]	NA	2217	1 [Reference]	NA	2392	1 [Reference]		NA	NA		NA		NA
APOE*4 dosage, female	8085	3.47 (3.27-3.69)	<1.0E−300^b^	2108	2.37 (2.10-2.67)	3.97E−45^b^	1163	1.94 (1.68-2.23)		1.75E−20^b^	2.12E−08^b^		5.22E−14^b^		.03^b^
APOE*2 dosage, male	1483	0.61 (0.53-0.70)	2.78E−12^b^	441	0.64 (0.46-0.91)	.01^b^	177	0.73 (0.50-1.06)		.10	.76		.39		.65
APOE*33, male	6925	1 [Reference]	NA	878	1 [Reference]	NA	1125	1 [Reference]		NA	NA		NA		NA
APOE*4 dosage, male	5718	3.51 (3.26-3.78)	1.43E−245^b^	865	2.50 (2.04-3.07)	1.94E−18^b^	552	1.85 (1.52-2.27)		2.00E−09^b^	2.28E−03^b^		5.51E−09^b^		.04^b^
APOE*2 dosage, sex interaction^c^	3760	0.87 (0.74-1.03)	.10	1411	1.06 (0.75-1.51)	.74	607	1.23 (0.82-1.85)		.32	.33		.13		.59
APOE*4 dosage, sex interaction^c^	13 803	0.93 (0.85-1.02)	.14	2973	0.95 (0.76-1.19)	.65	1715	1.04 (0.82-1.32)		.73	.90		.40		.57
*APOE* genotype															
APOE*22, female	68	0.32 (0.17-0.61)	5.36E−04^b^	44	0.87 (0.37-2.03)	.75^b^	17	1.58 (0.57-4.35)		.38	.07		9.52E−03^b^		.38
APOE*23, female	1738	0.50 (0.44-0.56)	7.00E−28^b^	691	0.72 (0.57-0.92)	8.33E−03^b^	343	0.93 (0.71-1.20)		.57	6.08E−03^b^		2.52E−05^b^		.17
APOE*33, female	10 274	1 [Reference]	NA	2217	1 [Reference]	NA	2392	1 [Reference]		NA	NA		NA		NA
APOE*24, female	471	2.21 (1.80-2.70)	1.66E−14^b^	235	1.55 (1.13-2.13)	7.16E−03^b^	70	1.03 (0.61-1.75)		.91	.07		8.46E−03^b^		.20
APOE*34, female	6359	3.54 (3.29-3.80)	1.81E−256^b^	1576	2.17 (1.85-2.54)	5.47E−22^b^	970	1.97 (1.67-2.33)		1.94E−15^b^	3.46E−08^b^		3.38E−10^b^		.41
APOE*44, female	1255	12.34 (10.26-14.84)	1.82E−156^b^	297	6.27 (4.72-8.35)	1.97E−36^b^	123	3.51 (2.30-5.36)		6.18E−09^b^	9.76E−05^b^		9.58E−08^b^		.03^b^
APOE*22, male	65	0.51 (0.28-0.95)	.03^b^	21	0.61 (0.16-2.32)	.47	7		0.22 (0.02-2.34)	.21	.81	.49		.46	
APOE*23, male	1148	0.59 (0.50-0.68)	7.95E−12^b^	321	0.61 (0.41-0.89)	.01^b^	139		0.77 (0.52-1.16)	.22	.88	.21		.39	
APOE*33, male	6925	1 [Reference]	NA	878	1 [Reference]	NA	1125		1 [Reference]	NA	NA	NA		NA	
APOE*24, male	270	2.32 (1.78-3.02)	5.38E−10^b^	99	1.92 (1.13-3.25)	.02^b^	31		1.39 (0.63-3.08)	.41	.53	.23		.51	
APOE*34, male	4448	3.38 (3.10-3.69)	8.96E−164^b^	657	2.21 (1.69-2.87)	4.98E−09^b^	456		1.75 (1.37-2.24)	8.05E−06^b^	2.64E−03^b^	6.93E−07^b^		.21	
APOE*44, male	1000	14.48 (11.53-18.20)	1.84E−116^b^	109	7.83 (4.65-13.17)	9.39E−15^b^	65		4.02 (2.17-7.46)	9.91E−06^b^	.03^b^	1.37E−04^b^		.11	
APOE*22, sex interaction^c^	133	0.70 (0.29-1.69)	.43	65	1.41 (0.30-6.65)	.66	24		8.33 (0.63-109.47)	.11	.44	.07		.25	
APOE*23, sex interaction^c^	2886	0.87 (0.72-1.06)	.17	1012	1.15 (0.74-1.78)	.54	482		1.19 (0.74-1.91)	.48	.27	.24		.92	
APOE*24, sex interaction^c^	741	0.92 (0.66-1.28)	.62	334	0.84 (0.47-1.51)	.56	101		0.75 (0.29-1.94)	.56	.79	.70		.85	
APOE*34, sex interaction^c^	10 807	0.98 (0.88-1.10)	.78	2233	1.00 (0.74-1.35)	.99	1426		1.14 (0.85-1.52)	.39	.93	.37		.54	
APOE*44, sex interaction^c^	2255	0.74 (0.56-0.98)	.04	406	0.85 (0.49-1.49)	.57	188		0.89 (0.42-1.85)	.75	.66	.65		.93	

Abbreviations: *APOE*, apolipoprotein E; NA, not applicable; OR, odds ratio.

^a^ Black and White individuals were non-Hispanic. Hispanic individuals included all race categories and other, which included those who did not identify with racial and ethnic categories provided.

^b^ Significance was considered at *P* < .05.

^c^ Male was the reference category.

**Figure 2.**
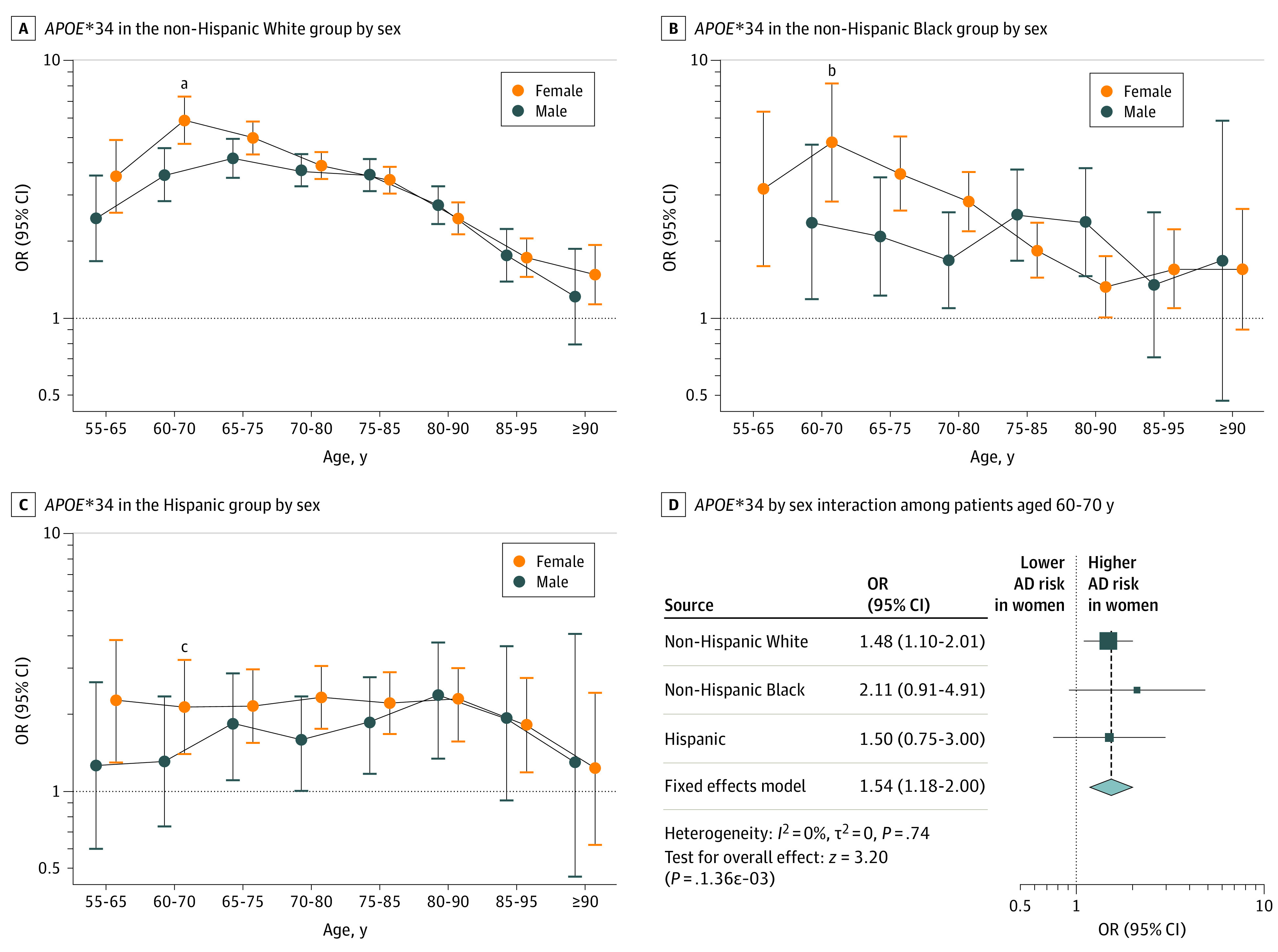
Associations of Apolipoprotein E (*APOE*)*34 Genotypes With Alzheimer Disease Risk Across Race and Ethnicity, Age, and Sex A-C, The age bin of 60 to 70 years indicated a significant APOE*34-by-sex association in non-Hispanic White individuals that was replicated with nominal significance upon meta-analysis of Hispanic individuals and non-Hispanic Black individuals (*P* = .048). D, Squares indicate odds ratios (ORs), with horizontal lines indicating 95% CIs. The diamond indicates the pooled OR, with outer points of the diamond indicating 95% CIs. ^a^*P* = .01 for interaction. ^b^*P* = .08 for interaction. ^c^*P* = .25 for interaction.

*APOE*-by-sex associations reached significance among White individuals for APOE*44, showing lower AD risk among women compared with men in non–age-stratified analyses (Table), and for APOE*34 at ages 60 to 70 years, showing an association with greater AD risk among women compared with men (Figure 2 and eFigure 4 in Supplement 1). In a meta-analysis of Black and Hispanic individuals, the APOE*44-by-sex effect estimate was concordant in direction (lower risk among women) but did not replicate and did not reveal an association with AD risk (OR, 0.86; 95% CI, 0.55-1.35; *P* = .52), while the APOE*34-by-sex association at ages 60 to 70 years was concordant in direction (greater risk among women) and replicated (OR, 1.72; 95% CI, 1.01-2.94; *P* = .046). The latter association remained consistent in sensitivity analyses (eFigure 5 in Supplement 1).

Findings from survival analyses across racial and ethnic groups were similar to case-control regression analyses (eFigure 6 and eTable 13 in Supplement 1) but indicated 2 notable differences. First, the lower risk of AD associated with APOE*2 became more prominent among Hispanic individuals (but remained higher than among Black individuals). Second, significant differences for APOE*4, particularly APOE*44, across Black and White individuals were lost. Survival analyses indicated no difference in the association of APOE*44 with AD risk across Black (hazard ratio [HR], 2.23; 95% CI, 2.07-2.40) and White (HR, 2.32; 95% CI, 2.27-2.38) individuals. Notably, a significant APOE*34-by-sex interaction was observed among White individuals (OR, 1.48; 95% CI, 1.10-2.01; *P* = 1.9 × 10^−3^), which replicated upon meta-analysis of Black and Hispanic individuals (OR, 1.72; 95% CI, 1.01-2.94; *P* = 2.3 × 10^−3^) (eFigure 6 in Supplement 1).

While among East Asian and Hispanic individuals there was no significant association of APOE*22 and APOE*23 with AD risk, there was a comparatively lower risk in these racial and ethnic groups for APOE*24 compared with APOE*34 (Figure 1A, Table, and eFigure 3 and eTable 6 in Supplement 1), suggesting a protective association of APOE*2 with risk of AD when seen together with APOE*4. Conversion risk analyses did indicate a significant association of APOE*23 with reduced AD risk among Hispanic individuals but further showed that the HR of APOE*24 was reduced among Hispanic individuals compared with Black individuals (which was not true in case-control regression analyses; eTable 13 in Supplement 1) and even displayed a protective HR. This again suggested a more protective HR for APOE*2 and AD risk when seen together with APOE*4.

### Global Population Ancestry Analyses

Focusing on global population ancestry effects, proportions of participants having at least 75% of a given population ancestry within racial and ethnic groups (determined through cohort demographics) were Black individuals with African (96.4%), Amerindian (0%), and European (0%) ancestry; Hispanic individuals with African (2.9%), Amerindian (10.8%), and European (7.0%) ancestry; and White individuals with African (0%), Amerindian (0%), and European (99.5%) ancestry (eFigure 2 in Supplement 1). Mean global population ancestries are shown in eTable 3 in Supplement 1. Global population ancestry could not explain why Hispanic individuals showed *APOE* associations with less pronounced AD risk compared with Black and White individuals (eTable 14 in Supplement 1). While decreased African or European ancestry or increased Amerindian ancestry might have contributed to *APOE* associations with reduced AD risk among Hispanic individuals, comparing less than 25% with more than 25% Amerindian ancestry strata showed no differences in associations with APOE*2 and APOE*4 dosages. Within Black individuals, decreased global African ancestry or increased global European ancestry showed a pattern of APOE*4 dosage associated with increasing AD risk, but no such pattern was apparent for APOE*2 dosage with AD risk (eTable 15 in Supplement 1). Within White individuals, we judged that there was insufficient ancestry variation to conduct ancestry-stratified analyses (eFigure 2 in Supplement 1).

Lastly, *APOE* associations with AD risk were evaluated across global population ancestry groups without regard or adjustment for race and ethnicity status: African (n = 5461), Amerindian (n = 621), and European (n = 34 021) ancestry. Case-control regression results are shown in eTable 16 in Supplement 1 and confirmed that differences across African and European ancestry were similar to those across Black and White individuals, although among individuals with African ancestry, the AD risk associated with APOE*4 was slightly diminished compared with that among Black individuals and there was no association of APOE*22 with AD risk.

## Discussion

Motivated by recent advances in AD-related genetic cohorts as well as state-of-the-art genotype and phenotype quality control, we provided, to our knowledge, the largest-to-date overview of the associations of *APOE* with AD risk across age, sex, race and ethnicity, and global population ancestry. Importantly, we also performed interaction and heterogeneity analyses to robustly evaluate stratified associations. Compared with prior work, we expanded sample sizes for all racial and ethnic groups, most notably for Black, East Asian, and Hispanic individuals, leading to important, novel insights.

Interestingly, the effect estimates for *APOE* genotypes and AD risk were least pronounced among Hispanic individuals, which was not explained by Black and White race or global ancestry differences. This observation of attenuated effect estimates for *APOE* and AD risk among Hispanic individuals were observed in a prior, smaller study (using overlapping samples) conducted by Blue et al in 2019.^25^ Although global ancestry did not show a consistent effect on modulating the ORs for *APOE* with AD risk in Hispanic individuals, there were suggestive effects whereby Amerindian and European ancestry modulated the ORs for APOE*44 with AD risk (eTable 14 in Supplement 1). Notably, an attenuated OR for APOE*44 with AD risk was observed with high Amerindian ancestry, which appears in line with a previously reported lack of APOE*4 associations with neurodegeneration among American Indian individuals.^26^ Furthermore, our findings indicated that differences in ORs for APOE*4 and AD risk were more pronounced when comparing African with European ancestry than when comparing Black with White individuals. Similarly, among Black individuals, higher African (or lower European) ancestry showed a pattern of reduced ORs (less risk increasing) for APOE*4 and AD risk. While the reduced effect of APOE*4 among Black individuals is an important, ongoing research question in the field, our observations suggest that research should focus additionally on African ancestry–specific investigations and on the further diminished *APOE* effect estimates among Hispanic individuals.

Given the relative paucity of APOE*2 carriers in prior studies of Black and Hispanic individuals, a robust assessment of the association of APOE*2 with AD risk among these groups remained hampered.^1,3^ We observed that APOE*2, in addition to APOE*4, showed a stepwise pattern of attenuated ORs following White, Black, and Hispanic individuals; there was no association of APOE*2 with AD risk among East Asian and Hispanic individuals. Despite that the APOE*2 allele frequency among East Asian individuals reported by Choi et al^20^ was less than half of that among White individuals, the total sample size of East Asian individuals in our meta-analyses should have been sufficiently large to observe a potential protective association. Global population ancestry did not explain attenuated (less protective) ORs for APOE*2 and AD risk among Black and Hispanic individuals compared with White individuals except for a lack of an association for APOE*22 at high global African ancestry (but not APOE*23). Overall, the findings suggest the need for more research to understand why the ORs for APOE*2 and AD risk were attenuated (less protective) among Black, East Asian, and Hispanic individuals, as well as with increased global African ancestry. It was additionally notable that among East Asian and Hispanic individuals, a protective association of APOE*2 was apparent when seen together with APOE*4. A similar observation was made recently for a sample of African American individuals in the National Alzheimer’s Coordinating Center.^27^ A biologic mechanism explaining this observation is, to our knowledge, not apparent, compelling further research into the already understudied APOE*24 genotype.

It should be noted that compared with Black and White individuals, Hispanic controls were a mean 5 years younger (eTable 3 in Supplement 1), which may have contributed to some diminishment of the ORs for *APOE* and AD risk. However, age-stratified analyses and survival analyses still revealed the most attenuated effect estimates for *APOE* genotypes and AD risk among Hispanic individuals. Another interesting finding was that secondary survival analyses suggested a loss of significant differences between Black and White individuals for AD conversion risk associated with APOE*4, particularly APOE*44. Similarly, case-control sensitivity analyses indicated that in non–community-based samples, among Black individuals, the associations of APOE*23 became more protective and those of APOE*34 and APOE*44 more risk increasing, such that the effect estimates became more similar to those for White individuals. These findings suggest that future studies should explore age-at-onset effects and the role of ascertainment design among racial and ethnic minority groups. It is also relevant to note that the effect estimates for APOE*2 and AD risk became more protective among Hispanic individuals when using survival analyses. Unfortunately, survival estimates could not be obtained for East Asian individuals, but it will be interesting to evaluate this in future studies.

Despite the novel 2023 National Academies of Sciences, Engineering, and Medicine guidelines on using population descriptors in genetics,^28^ it is relevant to note that we focused on the current racial and ethnic groups given their widespread use in AD (including genomics, clinical trials, and broad AD research) and previously established effects on stratifying *APOE*-related risk for AD.^3^ The choice was additionally motivated given that more granular information regarding population or environmental variables was not readily available across the various included cohorts. Although we showed that racial and ethnic groups were associated with ancestry groups (in line with expectations), our findings supported the notion that race and ethnicity descriptors should not be used as a proxy for genetic or biologic variation, most notably shown by the pattern of diminished effect estimates for APOE*4 in African ancestry compared with Black individuals. On the other hand, we showed that the racial and ethnic groups were relevant in revealing health disparities for the effects of APOE*2 and for Hispanic individuals since global population ancestry could not explain the observed differences in the *APOE* genotype associations with AD risk.

Lastly, the sex-by-age–specific association of APOE*34 with AD risk among White individuals (higher risk among women) was reproduced but shifted to ages 60 to 70 years (compared with ages 65-75 years in the previous largest analysis [N = 19 764], to our knowledge).^7^ The earlier shift in age may be explained by our harmonization of phenotype data (including age at onset). Expanding on these prior insights, we also replicated this association among Black and Hispanic individuals. Similarly, in survival analyses among White individuals, we reproduced the sex-by-APOE*34 interaction (greater risk among women) previously reported by Altmann et al^4^ in 2014 (N = 5496) and additionally replicated this among Black and Hispanic individuals. The interaction in the Altmann et al^4^ study appeared larger than in our study, which may be because they evaluated conversion from control status using longitudinal data, which in turn may have provided more accurate effect estimates. Overall, it was compelling that we observed consistency for the sex-by-APOE*34 association across multiple racial and ethnic groups, emphasizing that future studies and clinical trials with racial and ethnic minority populations should consider sex-by-APOE*4 interactions.

### Future Perspectives

The current study only considered global population ancestry, but prior work has suggested that local ancestry on *APOE* may be relevant to explain heterogeneity of *APOE* associations with AD risk.^25,29,30,31^ Local ancestry analyses can provide insight into the ancestral origin of the genetic information surrounding *APOE*, which may differ from the global average ancestry assessed across an individual’s entire genome. It is particularly interesting that ancestry-specific haplotypes on *APOE* may carry ancestry-specific variants that specifically modulate the association of APOE*2 or APOE*4 genotypes with AD risk. An example is provided by Griswold et al^32^ in 2021, suggesting that African compared with European ancestry haplotypes on APOE*4 reduced expression of APOE*4, which in turn may explain attenuated effect sizes for APOE*44 and AD risk in individuals of African ancestry compared with those of European ancestry. Our findings are thus important to guide future studies assessing *APOE* local ancestry across racial and ethnic groups. It will be particularly interesting to see whether local Amerindian ancestry may play a role in the diminished ORs for *APOE* among Hispanic individuals, although 2 prior, smaller studies may suggest the opposite.^25,33^ Despite the large increase in samples for racial and ethnic minority groups, additional data will be needed to further increase robustness, particularly for APOE*2 effects and *APOE*-by-sex associations and to add more Asian samples that are expected to become available through publicly available resources from the Alzheimer’s Disease Genetics Consortium and the Alzheimer’s Disease Sequencing Project. For the current East Asian samples, it is also relevant to note that these were based on Japanese and Korean samples, whereas effect estimates in Chinese,^34^ Indian,^35^ and Iranian^36^ populations may differ (Miyashita et al^37^ provides a relevant review). Similarly, it is relevant that more than half of the Hispanic individuals in this study were Caribbean Hispanic individuals (eTable 5 in Supplement 1), such that larger and more diverse groups of Hispanic individuals will need to be included in future work to obtain more representative results. Some gains in sample sizes may also be achieved by using mixed model approaches that integrate genetic relationship matrices to include related individuals.

### Limitations

The current study largely made use of cross-sectional data, which have obvious advantages in terms of power, but the addition of more longitudinal cognitive data should help provide more accurate effect estimates of the association of *APOE* genotypes with AD risk and allow modeling of effects on mild cognitive impairment. Along this line, it is also relevant that effect estimates obtained in this study were approximate and dependent on recruitment criteria in the AD case-control cohorts that were aggregated, whereas population-based studies may provide effect estimates more representative of the general population (eTable 11 in Supplement 1). It would further be relevant that future studies consider investigating differences in the rate of cognitive decline after symptom onset across racial and ethnic groups, as recent data mainly from White cohorts suggest neuropathology-independent, *APOE*-driven differences in rate of progression.^38,39^ Furthermore, the current study was unable to examine whether the attenuated AD risk associated with *APOE* in Black and Hispanic individuals was attributable to social determinants of health or clinical or modifiable risk factors moderating the association with *APOE*. Considering these limitations, novel data from ongoing diversity and phenotype harmonization efforts in AD genetics^14,15,16,40^ should enable future extensions on this study that will generate additional, valuable insights.

## Conclusions

We provided, to our knowledge, the largest-to-date overview of the associations of *APOE* with AD risk across age, sex, race and ethnicity, and global population ancestry. Our most notable observations were the following. First, ORs for *APOE* genotypes and AD risk were least pronounced among Hispanic individuals, which was not explained by global population ancestry. Second, there was a stepwise pattern of increasing ORs for APOE*2 (combination of APOE*22 and APOE*23) and AD risk following White, Black, and Hispanic individuals, with no association in East Asian and Hispanic individuals. Third, the sex-by-age–specific association of APOE*34 with AD risk among White individuals (greater risk among women) was reproduced but shifted to ages 60 to 70 years and was additionally replicated among Black and Hispanic individuals. Fourth, survival analyses indicated no difference in AD risk associated with APOE*44 across Black and White individuals, suggesting that future studies should evaluate age-at-onset effects among racial and ethnic minority populations. Overall, these novel insights should help guide AD research and clinical trial design.

## Notes

Supplement 1.
eMethods
**eAppendix.** Additional Contributions **eFigure 1.** Flowchart of Sample/Participant Filtering for *APOE* Association Analyses With Alzheimer Disease Risk **eFigure 2.** Admixture Plots Across the Five Major Super Populations, for Participants Included in Association Analyses **eFigure 3.** Alternative Visualizations of Case-Control Regression Results Presented in Figure 1 **eFigure 4.** Sex-and-Age Stratified Results for *APOE* Genotype Case-Control Regression Analyses Across Non-Hispanic White (NHW), Non-Hispanic Black (NHB), and Hispanic (HISP) Individuals **eFigure 5.** *APOE**34-by-Sex Interaction Effect in Case-Control Regression Analyses for Individuals 60 to 70 Years of Age Is Preserved Across Sensitivity Analyses **eFigure 6.** Survival Analyses Results, Through Cox Regression, Across *APOE* Genotypes, for Non-Hispanic White (NHW), Non-Hispanic Black (NHB), and Hispanic (HISP) Individuals **eTable 1.** Overview of Genotyping Platforms Across All Available AD-Related Genetic Data **eTable 2.** Overview of ADSP Studies With WES or WGS Available Through NIAGADS DSS (NG00067) **eTable 3.** Overview of Participant Demographics Across Race and Ethnicity and *APOE* Genotype Strata **eTable 4.** Overview of Participant Demographics Across the Cohort-Platform Technical Covariate **eTable 5.** Overview of Participant Demographics and Ascertainment Design Across Cohorts **eTable 6.** Case-Control Regression Results Across *APOE* Strata Corresponding to Figure 1a **eTable 7.** Case-Control Regression Results Across *APOE* Dosages and Strata for Hispanic Individuals (HISP), Stratified Into Hispanic Whites (HW) and Hispanic Blacks (HB) **eTable 8.** Sensitivity Case-Control Regression Analyses Corresponding to the Table Using Clinically Determined Phenotypes Only **eTable 9.** Sensitivity Case-Control Regression Analyses Corresponding to the Table Additionally Adjusting for Pathology Verification Status **eTable 10.** Sensitivity Case-Control Regression Analyses Corresponding to the Table and **eTable 10,** Using Only Samples From Cohorts With a Given Ascertainment Design **eTable 11.** Sensitivity Case-Control Regression Analyses Corresponding to the Table and **eTable 10,** Additionally Adjusting for Ascertainment Design **eTable 12.** Sensitivity Case-Control Regression Analyses Corresponding to the Table Removing Samples in Which Race and Ethnicity Status Was Not Directly Provided From Cohort Demographic Files **eTable 13.** Survival Analyses Results, Through Cox Regression, Across *APOE* Dosages and Genotypes, and Additionally Stratified Across Sex, for Non-Hispanic White (NHW), Non-Hispanic Black (NHB), and Hispanic (HISP) Individuals **eTable 14.** Case-Control Regression Results Across *APOE* Dosage and Strata, for Hispanic Individuals, Stratified Into Global Ancestry Quartiles **eTable 15.** Case-Control Regression Results Across *APOE* Dosage and Strata, for Non-Hispanic Black Individuals, Stratified Into Global Ancestry Quartiles **eTable 16.** Sensitivity Case-Control Regression Analyses Mirroring the Table, Considering Stratifications Across Global Population Ancestry Proportion Greater Than 75%
eReferences

Supplement 2.
Data Sharing Statement
