# Supplementary material for: APOE Genotype and Alzheimer Disease Risk Across Age, Sex, and Population Ancestry
Source: JAMA Neurol. 2023 Nov 6;80(12):1284–94. doi: 10.1001/jamaneurol.2023.3599 (PMC10628838; doi:10.1001/jamaneurol.2023.3599)
Supplement: Supplement 1. — eMethods eAppendix. Additional Contributions eFigure 1. Flowchart of Sample/Participant Filtering for APOE Association Analyses With Alzheimer Disease Risk eFigure 2. Admixture Plots Across the Five Major Super Populations, for Participants Included in Association Analyses eFigure 3. Alternative Visualizations of Case-Control Regression Results Presented in Figure 1 eFigure 4. Sex-and-Age Stratified Results for APOE Genotype Case-Control Regression Analyses Across Non-Hispanic White (NHW), Non-Hispanic Black (NHB), and Hispanic (HISP) Individuals eFigure 5. APOE*34-by-Sex Interaction Effect in Case-Control Regression Analyses for Individuals 60 to 70 Years of Age Is Preserved Across Sensitivity Analyses eFigure 6. Survival Analyses Results, Through Cox Regression, Across APOE Genotypes, for Non-Hispanic White (NHW), Non-Hispanic Black (NHB), and Hispanic (HISP) Individuals eTable 1. Overview of Genotyping Platforms Across All Available AD-Related Genetic Data eTable 2. Overview of ADSP Studies With WES or WGS Available Through NIAGADS DSS (NG00067) eTable 3. Overview of Participant Demographics Across Race and Ethnicity and APOE Genotype Strata eTable 4. Overview of Participant Demographics Across the Cohort-Platform Technical Covariate eTable 5. Overview of Participant Demographics and Ascertainment Design Across Cohorts eTable 6. Case-Control Regression Results Across APOE Strata Corresponding to Figure 1a eTable 7. Case-Control Regression Results Across APOE Dosages and Strata for Hispanic Individuals (HISP), Stratified Into Hispanic Whites (HW) and Hispanic Blacks (HB) eTable 8. Sensitivity Case-Control Regression Analyses Corresponding to the Table Using Clinically Determined Phenotypes Only eTable 9. Sensitivity Case-Control Regression Analyses Corresponding to the Table Additionally Adjusting for Pathology Verification Status eTable 10. Sensitivity Case-Control Regression Analyses Corresponding to the Table and eTable 10, Using Only Samples From Cohorts With [file jamaneurol-e233599-s001.pdf]

Supplemental Online Content

Belloy ME, Andrews SJ, Le Guen Y, et al. *APOE* genotype and Alzheimer disease risk across age, sex, and population ancestry. *JAMA Neurol*. Published online November 6, 2023. doi:10.1001/jamaneurol.2023.3599

eMethods
eAppendix. Additional Contributions
eFigure 1. Flow Chart of Sample/Participant Filtering for *APOE* Association Analyses With Alzheimer's Disease Risk
eFigure 2. Admixture Plots Across the Five Major Super Populations, for Participants Included in Association Analyses
eFigure 3. Alternative Visualizations of Case-Control Regression Results Presented in Figure 1
eFigure 4. Sex-and-Age Stratified Results for *APOE* Genotype Case-Control Regression Analyses Across Non-Hispanic White (NHW), Non-Hispanic Black (NHB), and Hispanic (HISP) Individuals
eFigure 5. *APOE**34-by-Sex Interaction Effect in Case-Control Regression Analyses for Individuals 60 to 70 Years of Age Is Preserved Across Sensitivity Analyses
eFigure 6. Survival Analyses Results, Through Cox Regression, Across *APOE* Genotypes, for Non-Hispanic White (NHW), Non-Hispanic Black (NHB), and Hispanic (HISP) Individuals
eTable 1. Overview of Genotyping Platforms Across All Available AD-Related Genetic Data
eTable 2. Overview of ADSP Studies With WES or WGS Available Through NIAGADS DSS (NG00067)
eTable 3. Overview of Participant Demographics Across Race and Ethnicity and *APOE* Genotype Strata
eTable 4. Overview of Participant Demographics Across the Cohort-Platform Technical Covariate
eTable 5. Overview of Participant Demographics and Ascertainment Design Across Cohorts
eTable 6. Case-Control Regression Results Across *APOE* Strata Corresponding to Figure 1a
eTable 7. Case-Control Regression Results Across *APOE* Dosages and Strata for Hispanic Individuals (HISP), Stratified Into Hispanic Whites (HW) and Hispanic Blacks (HB)
eTable 8. Sensitivity Case-Control Regression Analyses Corresponding to the Table, Using Clinically Determined Phenotypes Only
eTable 9. Sensitivity Case-Control Regression Analyses Corresponding to the Table, Additionally Adjusting for Pathology Verification Status
eTable 10. Sensitivity Case-Control Regression Analyses Corresponding to the Table and eTable 10, Using Only Samples From Cohorts With a Given Ascertainment Design
eTable 11. Sensitivity Case-Control Regression Analyses Corresponding to the Table and eTable 10, Additionally Adjusting for Ascertainment Design
eTable 12. Sensitivity Case-Control Regression Analyses Corresponding to the Table, Removing Samples in Which Race and Ethnicity Status Was Not Directly Provided From Cohort Demographic Files
eTable 13. Survival Analyses Results, Through Cox Regression, Across *APOE* Dosages and Genotypes, and Additionally Stratified Across Sex, for Non-Hispanic White (NHW), Non-Hispanic Black (NHB), and Hispanic (HISP) Individuals
eTable 14. Case-Control Regression Results Across *APOE* Dosage and Strata, for Hispanic Individuals, Stratified Into Global Ancestry Quartiles
eTable 15. Case-Control Regression Results Across *APOE* Dosage and Strata, for Non-Hispanic Black Individuals, Stratified Into Global Ancestry Quartiles

© 2023 Belloy ME et al. *JAMA Neurology.*

eTable 16. Sensitivity Case-Control Regression Analyses Mirroring the Table, Considering Stratifications Across Global Population Ancestry Proportion Greater Than 75%
eReferences

eMethods

In the current study, we used data from a variety of cohorts and sequencing projects related to AD[1-23]. All available genetic/phenotypic data were jointly harmonized with the purpose of performing phenotype/covariate harmonization. Details are provided below.

Phenotype Ascertainment

Cohorts and Phenotype Ascertainment

Details on phenotype ascertainment are described elsewhere[1–6]. Briefly, all individuals with a diagnosis of AD met National Institute of Neurological and Communicative Disorders and Stroke/Alzheimer's Disease and Related Disorders Association (NINCDS-ADRDA) criteria for definite, probable, or possible late onset AD[7], or met Diagnosis and Statistical Manual of Mental Disorders IV-V (DSMIV-V) criteria[8–10], or had a clinical dementia rating (CDR® Dementia Staging Instrument[11]) > 0.5. Some cohorts verified AD diagnoses by means of neuropathology, using Braak staging[12], CERAD scoring[13], or National Institute on Aging Reagan (NIA-Reagan) 1997 criteria[14]. Cognitively normal subjects did not have AD according to the above clinical AD criteria, did not have a diagnosis of mild-cognitive impairment (MCI), and had a CDR of 0 and/or Mini-Mental State Examination (MMSE[15]) > 25. In the MIRAGE cohort, control status was evaluated through a Modified Telephone Interview of Cognitive Status score ≥ 86 (a telephone version of the MMSE)[16].

Further, the National Alzheimer's Coordinating Center (NACC), Rush University Religious Orders Study/Memory and Aging Project (ROSMAP), and Alzheimer's Disease Neuroimaging Initiative (ADNI), are longitudinal cohorts that provide detailed information regarding clinical status (control, MCI, demented) and presumed disease etiology at repeated examinations. Additionally, deceased subjects are assessed for neuropathology. Where possible, in NACC, a final diagnoses of MCI or possible/probable/definite AD was obtained using NIA Alzheimer's Association (NIA-AA) 2011 criteria[17,18]. In all three cohorts, AD diagnoses were verified by neuropathology as middle or high AD likelihood following NIA-Reagan 1997 criteria (moderate to frequent neuritic plaques and Braak stage III-VI)[14]. In concordance with the category "possible AD dementia with evidence of the AD pathophysiological process" from the NIA-AA 2011 criteria[17], we attributed possible AD diagnoses to subjects who met clinical criteria for non-AD dementia but also met AD neuropathological criteria. In concordance with the NIA-AA 2011/2012 framework[18,19], we also evaluated neuropathology in MCI subjects to verify presumed AD etiology (cf. page 5). Controls were not re-evaluated based on neuropathology data. Subjects that reverted from dementia to control status during longitudinal follow-up were excluded. Additional cohort-specific details are listed below.

NACC

Genotyping waves 1 through 7 from the Alzheimer's Disease Centers (ADC1-7) and a subset of the ADSP projects include subjects ascertained and evaluated by the clinical and neuropathological cores of 32 NIA-funded ADCs. NACC coordinates the collection of these phenotypes, implements diagnoses (cognitively normal, cognitively impaired but not MCI, MCI, demented; and presumed disease etiology) and then provides all data to researchers under the form of the Minimum Data Set (MDS), Uniform Data Set (UDS)[20–22], and Neuropathology data set (NP)[23]. The MDS represents an older subset of the NACC data and only contains cross-sectional data, while the more recent UDS provides longitudinal phenotypes and covariates. Since 2015, the UDS was updated to incorporate the NIA-AA 2011 criteria for MCI and AD[18,24]. In the current study, we used the UDS and NP for which data was collected between September 2005 and March 2022, to determine phenotypes for subjects in ADC1-7, ADSP WES/WGS, and ADGC Exome arrays.

Subjects that had a diagnosis of Down syndrome, central nervous system neoplasm, bipolar disorder, schizophrenia, alcohol-induced dementia, or substance-abuse-induced dementia, were excluded. Subjects carrying mutations of dominantly inherited AD or frontotemporal lobar degeneration (FTLD) were also excluded. Subjects with a final diagnosis of MCI or dementia, for which the etiology was unknown, not due to AD, or only secondary due to AD (and without AD neuropathological information), were excluded. Subjects with a final diagnosis of "cognitively impaired but not MCI", but having no other neurological disorder, were kept as controls, considering that this more consistently matched control criteria in many of the other cohorts considered in this study.

ROSMAP

In ROSMAP, subjects were diagnosed at each visit: as possible/probable AD according to NINCDS-ADRDA criteria[7]; as MCI when judged to have cognitive impairment but not meeting dementia criteria according to the clinician; or as control when there was no cognitive impairment or the subject did not meet dementia criteria[25,26]. At time of death, a final clinical diagnosis was made by an expert neurologist, followed by case conference consensus review (blinded to postmortem data)[27].

ADNI

In ADNI, subjects were diagnosed at regular visits: as possible/probable AD according to NINCDS-ADRDA criteria[7]; as MCI according to Petersen/Winblad criteria; or as control when not demented, not MCI, CDR = 0, and MMSE > 28. Neuropathology assessments followed the NACC NP framework.

Phenotype Harmonization

The available sample contained many subjects that were genotyped multiple times across different studies. This largely reflected efforts from the ADGC, ADSP, and AMP-AD, to perform next generation sequencing (NGS) on existing cohort samples for the purpose of rare variant discovery and AD gene prioritization. In other instances, participants were recruited in different studies at different times. Therefore, to handle potential duplicate discordance and phenotype heterogeneity, we implemented a cross-sample phenotype harmonization procedure aiming to standardize pathology-verified diagnoses where possible, share unique missing information across all duplicate entries of a given subject, resolve longitudinal changes in diagnosis, and flag subjects with unresolvable duplicate discordance for exclusion.

Duplicate samples were identified by determining genetic cryptic relatedness (cf. below), but for the purpose of sample cross-referencing did not include known identical twins in LOAD and ROSMAP samples. First, duplicate samples were flagged as discordant if their age-at-death information differed by more than 2 years or if pathology measures (Braak or neuritic plaque density) differed. Across all cohorts, where possible, AD diagnoses were verified by neuropathology as middle or high AD likelihood following NIA-Reagan 1997 criteria (moderate to frequent neuritic plaques and Braak stage III-VI)[14]. Additionally, when only either neuritic plaque or Braak information was available and in line with NIA-Reagan 1997 middle or high AD likelihood criteria, and/or the cohort/project demographics provided a diagnosis of definite AD, the subject was considered to have pathology-verified AD status. Cognitively normal (CN) subjects with evidence of AD pathology were kept as CN. Further, if at least one entry across duplicate samples indicated a diagnosis of Down syndrome, central nervous system neoplasm, bipolar disorder, schizophrenia, alcohol-induced dementia, substance-abuse-induced dementia, neurological (not including Parkinson's disease) or systemic disease despite being cognitively normal, or carrying mutations of dominantly inherited AD or frontotemporal lobar degeneration (FTLD), then all duplicate samples were marked as such and flagged for exclusion. Extending on the above, all genetic samples were checked for the presence of known pathogenic mutations on *APP, PSEN1, PSEN2* and *MAPT*, whereby carriers and their duplicate samples were flagged for exclusion.

Then, duplicate samples with differing age entries (i.e. longitudinal changes) were evaluated. Reversions from AD or dementia to MCI status, or from MCI to cognitively normal (CN) status, were permitted, but reversions from AD or non-AD dementia to CN status were flagged for exclusion. "Reversions" from AD to non-AD dementia status were permitted, unless pathology (cf. above) indicated the presence of AD pathology, thereby marking the subject as AD. Vice versa, "conversions" from non-AD

dementia to AD status were permitted, unless pathology (cf. above) indicated no presence of AD pathology, thereby marking the subject as non-AD dementia. All other types of conversions were directly permitted. Then, duplicate samples for which the diagnoses at the oldest shared age entries differed, or for which diagnoses differed but age was consistent (i.e. apparent cross-sectional discordances), were evaluated. Discordances between AD and non-AD dementia status were resolved on the basis of pathology (cf. above) or flagged as discordant if no pathology data was available. Discordances between CN and AD status, or CN and non-AD dementia status, were resolved as respectively AD or non-AD dementia when those dementia diagnoses corresponded to a unique age-at-onset (of symptoms) without other available age information (i.e. indicating that a conversion likely occurred after the subject was lost to follow-up in the cohort that last observed a CN status), or, were flagged as discordant if duplicate entries shared the same age-at-examination and age-at-last-exam. Discordances between CN and MCI status, or MCI and AD status, or MCI and non-AD dementia status, were resolved as respectively MCI, AD, or non-AD dementia (i.e. keeping the most severe diagnosis).

Finally, once all clinical diagnostic and pathological data were unified across duplicate entries, pathological criteria were applied once more to obtain the final diagnoses. Where possible, AD diagnoses were verified by neuropathology as middle or high AD likelihood following NIA-Reagan 1997 criteria (moderate to frequent neuritic plaques and Braak stage III-VI)[14]. In concordance with the category "possible AD dementia with evidence of the AD pathophysiological process" from the NIA-AA 2011 criteria[17], we attributed possible AD diagnoses to subjects who met clinical criteria for non-AD dementia but also met AD neuropathological criteria. In concordance with the NIA-AA 2011/2012 framework[18,19], we also evaluated neuropathology in MCI subjects to verify presumed AD etiology and considered subjects as cases if AD pathology, following NIA-Reagan 1997 criteria (cf. above), was present (i.e. marking high likelihood of AD etiology). Controls were not re-evaluated based on neuropathology data.

Beyond cross-referencing clinical diagnostic and pathological data across subjects, other covariates were considered for cross-referencing or sharing in case of missingness across duplicate entries. These included age-at-onset of cognitive symptoms, age-at-examination providing clinical diagnosis, at-at-last exam, age-at-death, sex, race, ethnicity, *APOE* genotype provided from demographics, *APOE* genotype provided from whole-genome sequencing, and *APOE* genotype provided from whole-exome sequencing. Duplicate entries with discordant sex or race information were flagged for exclusion.

© 2023 Belloy ME et al. *JAMA Neurology.*

Genetic Data Quality Control and Processing

Genetic Data Harmonization and Standard Quality Control

Genotypes were available from commercial high-density single-nucleotide polymorphism (SNP) genotyping microarrays (Illumina or Affymetrix), Exome microarrays, Exome sequencing (ES), or Genome sequencing (GS) (**eTable 1-2**). Genotype samples had their genetic variants lifted to hg38 using liftOver if not released in hg38[28]. Autosomal variants were extracted from the SNP array data and further processed in several stages. First, SNP array data were processed by the Genotype Harmonizer with CEU and TSI HapMap populations as the reference panel, to perform automatic strand alignment[29]. Then, multi-allelic SNPs, SNPs located on common copy number or segmental duplication regions, and duplicated or monomorphic SNPs, were removed. The list of multi-allelic SNPs or SNPs located on common copy number and segmental duplication regions was created using Tri-Typer[30]. The list of CNV and segmental duplication regions was curated from the Eichler lab (eichlerlab.gs.washington.edu/database.html)[31] and the gnomAD website (gnomad.broadinstitute.org/downloads)[32]. All respective genotype data sets were then iteratively merged with each other, applying strand flipping and variant ID updating as applicable, to ultimately obtain parsimonious data sets that could be merged for cross-sample relationship determination and principal component analyses (cf. below).

Genetic data were then further processed using Plink v1.9. For each sample platform, subjects with autosome missingness (≥ 5%) and sex problems (discordance between genetic sex and demographic sex, or deviation of expected X-chromosome homozygosity/heterozygosity) were flagged for exclusion.

Ancestry Determination

Individual ancestries were determined using SNPweights v.2.1 with populations from the 1000 Genomes Consortium as a reference[33,34]. By applying an ancestry percentage cut-off ≥ 75%, the samples were stratified into the five super populations, South-Asians (SAS), East-Asians (EAS), Amerindians (AMR), Africans (AFR) and Europeans (EUR) (**eFigure 2**). When multiple samples were available for a single unique individual, the ancestry was inferred from the sample with the highest genetic coverage.

Genetic Relationship Determination using King

Across all cohorts the relatedness of subjects (after QC indicated above) was evaluated through identity-by-descent (IBD) analysis (using directly genotyped non-palindromic SNPs that shared across all genetic datasets with a call rate > 95%, minor allele frequency (MAF) > 1%). This outcome was used for

© 2023 Belloy ME et al. *JAMA Neurology.*

duplicate tracking across samples, which in turn was used to enable phenotype harmonization, and to identify first-degree related samples, which in turn was used to retain only one individual per relatedness cluster in *APOE* dosage and genotype association analyses.

APOE genotype assessment in ADSP WES/WGS

In ADSP WGS, the rs429358 and rs7412 variants showed low genotype missingness across subjects, reflecting good variant quality metrics. In ADSP WES, there was a high genotype missingness at rs7412 (32.5%). This resulted from a low read depth and genotype quality in some of the different WES capture kits that were used in the ADSP WES[2]. We therefore sought to re-call both variants in order to fill out missing *APOE* information where possible. We first inferred the variants' genotype using data called by the ADSP, which required a read depth read depth (DP) >=10 and genotype quality (GQ) >= 20. We then further inferred the variants' genotype if DP and GQ were respectively greater than or equal to 6 and 20, observing at least 20% alternate allele reads to call a heterozygote (e.g. *APOE**3/4).

After this first round of *APOE* genotype ascertainment, some individuals still had either the rs7412 or rs429358 genotype missing (i.e., only one of the two variants could be called using the above criteria), making it impossible to infer their *APOE* genotype from the ADSP NGS data alone. Many of these remaining individuals however had a reported *APOE* genotype in their demographics that could be used to complete the missing information in a second additional round of *APOE* genotype ascertainment. This approach was preferred over relying solely on the *APOE* genotype in the demographics, since the genotype calls on the ADSP NGS data are expected to provide higher accuracy compared to other commonly used *APOE* direct genotyping methods[36]. To illustrate, consider the example where one of these remaining individuals in the sequencing data was homozygous for the reference allele at rs429358, which would suggest the subject is *APOE**3/3, but had a missing genotype at rs7412. In this case, from the ADSP NGS data, we know that this individual is not carrying an *APOE**4 allele, but we cannot determine the presence or absence of an *APOE**2 allele. We then turned to the information from the *APOE* genotype provided in the demographics to infer the most likely *APOE* genotype. For the current example, if the individual has a provided *APOE* genotype that was 2/2, 2/3, or 3/3, then the information in the ADSP NGS data is deemed concordant with the provided *APOE* genotype (that is, rs429358 is always the reference allele for those provided *APOE* genotypes) and we used the provided *APOE* genotype. However, if the provided *APOE* genotype was 4/4 or 3/4, then we would correct it to *APOE**3/3, because the ADSP NGS information clearly indicated there was no *APOE**4 genotype call (similarly a provided *APOE**2/4 genotype would be corrected to *APOE**2/3). This can be generalized as: for remaining individuals with DP>=6 and

© 2023 Belloy ME et al. *JAMA Neurology.*

GQ>=20 at rs429358, the ADSP NGS data at rs429358 was used to change, when discordant, the provided *APOE**3 genotype to *APOE**4, or vice-versa. One additional extension to this step was implemented for the few scenarios where the ADSP NGS data called two rs429358 alleles (i.e. *APOE**4/4) but the allelic distribution indicated that the reference allele was still observed (e.g. 1 REF allele and 7 ALT alleles). In these situations, if the provided *APOE* genotype indicated the presence of *APOE**3, then the genotype was corrected to *APOE**3/4 (reasoning there is sufficient evidence to support the presence of an *APOE**3 genotype). The extra checks described in this paragraph were also applied to subjects in the first QC round (prior paragraph), who had 6<=DP<10 and GQ>=20 for both rs429358 and rs7412.

As a quality check, using these thresholds, we did not observe any discordance in the inferred *APOE* genotype across 3,499 duplicates between the ADSP WGS and ADSP WES.

APOE genotype consensus harmonization and imputation

We used the consensus *APOE* genotyping approach as described in Belloy et al. 2022[37], namely, to prioritize first WGS/WES *APOE**2/3/4 genotypes if available (and if only either rs429358 or rs7412 is available from WGS/WES, to use those genotype data to verify the provided/demographic *APOE**2/3/4 genotypes); second to use provided/demographic *APOE**2/3/4 genotypes; and third, in subjects without WGS/WES information, to exclude those for whom the provided/demographic and imputed (R2>0.8) *APOE**2/3/4 genotypes are discordant. Regarding imputation, SNP array data were used to perform genotype imputation with regard to the TOPMed imputation reference panel[38,39], which was performed per SNP array and per sample groups of >75% European ancestry, >75% African ancestry, >75% Amerindian ancestry, or admixed samples where all global ancestries where <75%. The final step to ensure the highest quality of *APOE**2/3/4 genotypes was to verify and harmonize *APOE* genotype information across available duplicate samples.

Race and Ethnicity Ascertainment

Race and ethnicity (Hispanic versus non-Hispanic) information was directly available for a large fraction of samples (87.4% and 79.9% respectively). In many instances, this missing race information could be inferred from the respective cohorts/studies that contributed the samples (and often corresponds to White samples). Missing ethnicity information similarly reflected that related cohort/studies mainly recruited non-Hispanic samples and as such, we considered missing ethnicity information as samples being non-Hispanic. Based on prior observations of higher European ancestry in non-Hispanic Whites and higher African ancestry in non-Hispanic Blacks, and in an attempt to reduce potential ambiguity about

© 2023 Belloy ME et al. *JAMA Neurology.*

missing race status in available genetic samples, we used ancestry information in some individuals to inform on race status. For Non-Hispanic White (NHW) individuals, 18.4% of samples included in the final association analyses did not have race information directly available in cohort covariate files, but these subjects showed a global European ancestry >75%. For Non-Hispanic Black (NHB) individuals, 11.3% of samples included in the final association analyses did not have race information directly available in cohort covariate files, but these subjects showed a global African ancestry >75%. For Hispanic (HISP) individuals, ethnicity information was always available and indicated Hispanic status. Within HISP subjects, for Hispanic White (HW) individuals, 62.9% of samples included in the final association analyses did not have race information directly available in cohort covariate files, but these subjects showed a global European ancestry >75%, and, for Hispanic Black (HB) individuals, 87.2% of samples included in the final association analyses did not have race information directly available in cohort covariate files, but these subjects showed a global African ancestry >75%.

Statistical Analyses

Design of statistical analyses and General model criteria

All association analyses with AD risk (primary) or survival (secondary) adjusted for sex, array/batch/center, and global European, African, and Amerindian ancestry. Other technical covariates were considered in respective sensitivity analyses. Age adjustment in case-control analyses was not performed, given that the current AD genetic samples often showed younger ages for cases than controls due to the use of age-at-onset information (**eTable3**), which violates the assumption for age adjustment (which is that older age is associated with increased AD incidence). In prior work, we showed that age adjustment in such scenarios leads to significantly decreased power for genetic association analyses[40]. To avoid concern given the lack of age adjustment (which may be relevant particularly for HISP where ages in controls was on average higher than ages in controls), secondary survival analyses provide alternative insight into *APOE* genotype associations with AD by directly integrating age-at-onset data.

All association analyses evaluated the effect *APOE**2 dosage, *APOE**4 dosage, or *APOE* genotype with regard to *APOE**33 as the reference. Dosage effects were evaluated as a scalar, additive genetic model. All interaction between *APOE* dosage or genotype with sex were evaluated through formal interaction tests (i.e. an *APOE*-by-SEX variable in the association model in addition to *APOE* and sex variables). Differences across race/ethnicity groups were evaluated through heterogeneity tests, using the following formulation: Z-value = $(Beta_{Group1} - Beta_{Group2})/\sqrt{(SE_{Group1}^2 + SE_{Group2}^2)}$. P-values were then determined

using the normal distribution with a two-sided hypothesis in R using the following formulation: P-value = 2*pnorm(q=Z-value, lower.tail=FALSE).

Age information

For cases that only had age-at-death (AAD) available, the final ages used for regression analysis were subtracted by 10 years in order to approximate age-at-onset (AAO). This reflects expected mean delays between AAO and AAD for AD patients[41], and is consistent with the derived age covariate for AD cohorts provided by the Alzheimer's Disease Genetics Consortium (ADGC) on NIAGADS[42]. In cohorts that provide conversion information but not AAO, age-at-examination (AAE) was used and followed a prioritization of age-at-MCI-diagnosis > age-at-dementia-diagnosis (incident) > age-at-dementia diagnosis (prevalent). This was done to most closely approximate AAO. For the remaining control samples, age-at-last-examination (AAL) was used. After implementing these criteria, samples were filtered to have a minimal age of 55 years. Some samples were censored at ages 90+, for which we assumed the age was 90.

Multivariate Case-Control Logistic Regression Analyses

Case-control analyses were conducted using the standard *glm* function in R (v.4.2.1). In age-stratified analyses, the oldest age window was 90+, meaning that there was no concern for the use of samples censored at ages 90+.

Multivariate Cox Proportional Hazards Regression Analyses

Survival analyses were conducted using the *coxph* and *Surv* functions from the "survival" package in R (v.4.2.1). In these time-to-event analyses, events were defined at AAO (or best approximation) for individuals who developed AD (i.e. conversion to AD). Controls were right-censored at AAD or AAL. Left censoring was set at age 55 years. Individuals with ages censored at 90+ were removed from analyses.

Meta-Analysis

Meta-analyses were conducted using the *metagen* function from the "meta" package in R (v.4.2.1).

eAppendix. Additional Contributions

Acknowledgments for the use of ADSP WES and WGS data: The Alzheimer's Disease Sequencing Project (ADSP) is comprised of two Alzheimer's Disease (AD) genetics consortia and three National Human Genome Research Institute (NHGRI) funded Large Scale Sequencing and Analysis Centers (LSAC). The two AD genetics consortia are the Alzheimer's Disease Genetics Consortium (ADGC) funded by NIA (U01 AG032984), and the Cohorts for Heart and Aging Research in Genomic Epidemiology (CHARGE) funded by NIA (R01 AG033193), the National Heart, Lung, and Blood Institute (NHLBI), other National Institute of Health (NIH) institutes and other foreign governmental and non-governmental organizations. The Discovery Phase analysis of sequence data is supported through UF1AG047133 (to Drs Schellenberg, Farrer, Pericak-Vance, Mayeux, and Haines); U01AG049505 to Dr Seshadri; U01AG049506 to Dr Boerwinkle; U01AG049507 to Dr Wijsman; and U01AG049508 to Dr Goate and the Discovery Extension Phase analysis is supported through U01AG052411 to Dr Goate, U01AG052410 to Dr Pericak-Vance and U01 AG052409 to Drs Seshadri and Fornage. Sequencing for the Follow Up Study (FUS) is supported through U01AG057659 (to Drs PericakVance, Mayeux, and Vardarajan) and U01AG062943 (to Drs Pericak-Vance and Mayeux). Data generation and harmonization in the Follow-up Phase is supported by U54AG052427 (to Drs Schellenberg and Wang). The FUS Phase analysis of sequence data is supported through U01AG058589 (to Drs Destefano, Boerwinkle, De Jager, Fornage, Seshadri, and Wijsman), U01AG058654 (to Drs Haines, Bush, Farrer, Martin, and Pericak-Vance), U01AG058635 (to Dr Goate), RF1AG058066 (to Drs Haines, Pericak-Vance, and Scott), RF1AG057519 (to Drs Farrer and Jun), R01AG048927 (to Dr Farrer), and RF1AG054074 (to Drs Pericak-Vance and Beecham). The ADGC cohorts include: Adult Changes in Thought (ACT) (UO1 AG006781, UO1 HG004610, UO1 HG006375, U01 HG008657), the Alzheimer's Disease Centers (ADC) (P30 AG019610, P30 AG013846, P50 AG008702, P50 AG025688, P50 AG047266, P30 AG010133, P50 AG005146, P50 AG005134, P50 AG016574, P50 AG005138, P30 AG008051, P30 AG013854, P30 AG008017, P30 AG010161, P50 AG047366, P30 AG010129, P50 AG016573, P50 AG016570, P50 AG005131, P50 AG023501, P30 AG035982, P30 AG028383, P30 AG010124, P50 AG005133, P50 AG005142, P30 AG012300, P50 AG005136, P50 AG033514, P50 AG005681, and P50 AG047270), the Chicago Health and Aging Project (CHAP) (R01 AG11101, RC4 AG039085, K23 AG030944), Indianapolis Ibadan (R01 AG009956, P30 AG010133), the Memory and Aging Project (MAP) (R01 AG17917), Mayo Clinic (MAYO) (R01 AG032990, U01 AG046139, R01 NS080820, RF1 AG051504, P50 AG016574), Mayo Parkinson's Disease controls (NS039764, NS071674, 5RC2HG005605), University of Miami (R01 AG027944, R01 AG028786, R01 AG019085, IIRG09133827, A2011048), the Multi-Institutional Research in Alzheimer's Genetic Epidemiology Study

(MIRAGE) (R01 AG09029, R01 AG025259), the National Cell Repository for Alzheimer's Disease (NCRAD) (U24 AG21886), the National Institute on Aging Late Onset Alzheimer's Disease Family Study (NIA-LOAD) (R01 AG041797), the Religious Orders Study (ROS) (P30 AG10161, R01 AG15819), the Texas Alzheimer's Research and Care Consortium (TARCC) (funded by the Darrell K Royal Texas Alzheimer's Initiative), Vanderbilt University/Case Western Reserve University (VAN/CWRU) (R01 AG019757, R01 AG021547, R01 AG027944, R01 AG028786, P01 NS026630, and Alzheimer's Association), the Washington Heights-Inwood Columbia Aging Project (WHICAP) (RF1 AG054023), the University of Washington Families (VA Research Merit Grant, NIA: P50AG005136, R01AG041797, NINDS: R01NS069719), the Columbia University HispanicEstudio Familiar de Influencia Genetica de Alzheimer (EFIGA) (RF1 AG015473), the University of Toronto (UT) (funded by Wellcome Trust, Medical Research Council, Canadian Institutes of Health Research), and Genetic Differences (GD) (R01 AG007584). The CHARGE cohorts are supported in part by National Heart, Lung, and Blood Institute (NHLBI) infrastructure grant HL105756 (Psaty), RC2HL102419 (Boerwinkle) and the neurology working group is supported by the National Institute on Aging (NIA) R01 grant AG033193. The CHARGE cohorts participating in the ADSP include the following: Austrian Stroke Prevention Study (ASPS), ASPS-Family study, and the Prospective Dementia Registry-Austria (ASPS/PRODEM-Aus), the Atherosclerosis Risk in Communities (ARIC) Study, the Cardiovascular Health Study (CHS), the Erasmus Rucphen Family Study (ERF), the Framingham Heart Study (FHS), and the Rotterdam Study (RS). ASPS is funded by the Austrian Science Fond (FWF) grant number P20545-P05 and P13180 and the Medical University of Graz. The ASPS-Fam is funded by the Austrian Science Fund (FWF) project I904),the EU Joint Programme - Neurodegenerative Disease Research (JPND) in frame of the BRIDGET project (Austria, Ministry of Science) and the Medical University of Graz and the Steiermärkische Krankenanstalten Gesellschaft. PRODEM-Austria is supported by the Austrian Research Promotion agency (FFG) (Project No. 827462) and by the Austrian National Bank (Anniversary Fund, project 15435. ARIC research is carried out as a collaborative study supported by NHLBI contracts (HHSN268201100005C, HHSN268201100006C, HHSN268201100007C, HHSN268201100008C, HHSN268201100009C, HHSN268201100010C, HHSN268201100011C, and HHSN268201100012C). Neurocognitive data in ARIC is collected by U01 2U01HL096812, 2U01HL096814, 2U01HL096899, 2U01HL096902, 2U01HL096917 from the NIH (NHLBI, NINDS, NIA and NIDCD), and with previous brain MRI examinations funded by R01-HL70825 from the NHLBI. CHS research was supported by contracts HHSN268201200036C, HHSN268200800007C, N01HC55222, N01HC85079, N01HC85080, N01HC85081, N01HC85082, N01HC85083, N01HC85086, and grants U01HL080295 and U01HL130114 from the NHLBI with additional contribution from the National

Institute of Neurological Disorders and Stroke (NINDS). Additional support was provided by R01AG023629, R01AG15928, and R01AG20098 from the NIA. FHS research is supported by NHLBI contracts N01-HC-25195 and HHSN268201500001I. This study was also supported by additional grants from the NIA (R01s AG054076, AG049607 and AG033040 and NINDS (R01 NS017950). The ERF study as a part of EUROSPAN (European Special Populations Research Network) was supported by European Commission FP6 STRP grant number 018947 (LSHG-CT-2006-01947) and also received funding from the European Community's Seventh Framework Programme (FP7/2007-2013)/grant agreement HEALTH-F4-2007-201413 by the European Commission under the programme "Quality of Life and Management of the Living Resources" of 5th Framework Programme (No. QLG2-CT-2002- 01254). High-throughput analysis of the ERF data was supported by a joint grant from the Netherlands Organization for Scientific Research and the Russian Foundation for Basic Research (NWO-RFBR 047.017.043). The Rotterdam Study is funded by Erasmus Medical Center and Erasmus University, Rotterdam, the Netherlands Organization for Health Research and Development (ZonMw), the Research Institute for Diseases in the Elderly (RIDE), the Ministry of Education, Culture and Science, the Ministry for Health, Welfare and Sports, the European Commission (DG XII), and the municipality of Rotterdam. Genetic data sets are also supported by the Netherlands Organization of Scientific Research NWO Investments (175.010.2005.011, 911-03-012), the Genetic Laboratory of the Department of Internal Medicine, Erasmus MC, the Research Institute for Diseases in the Elderly (014-93-015; RIDE2), and the Netherlands Genomics Initiative (NGI)/Netherlands Organization for Scientific Research (NWO) Netherlands Consortium for Healthy Aging (NCHA), project 050-060-810. All studies are grateful to their participants, faculty and staff. The content of these manuscripts is solely the responsibility of the authors and does not necessarily represent the official views of the National Institutes of Health or the U.S. Department of Health and Human Services. The FUS cohorts include: the Alzheimer's Disease Centers (ADC) (P30 AG019610, P30 AG013846, P50 AG008702, P50 AG025688, P50 AG047266, P30 AG010133, P50 AG005146, P50 AG005134, P50 AG016574, P50 AG005138, P30 AG008051, P30 AG013854, P30 AG008017, P30 AG010161, P50 AG047366, P30 AG010129, P50 AG016573, P50 AG016570, P50 AG005131, P50 AG023501, P30 AG035982, P30 AG028383, P30 AG010124, P50 AG005133, P50 AG005142, P30 AG012300, P50 AG005136, P50 AG033514, P50 AG005681, and P50 AG047270), Alzheimer's Disease Neuroimaging Initiative (ADNI) (U19AG024904), Amish Protective Variant Study (RF1AG058066), Cache County Study (R01AG11380, R01AG031272, R01AG21136, RF1AG054052), Case Western Reserve University Brain Bank (CWRUBB) (P50AG008012), Case Western Reserve University Rapid Decline (CWRURD) (RF1AG058267, NU38CK000480), CubanAmerican Alzheimer's Disease Initiative (CuAADI)

© 2023 Belloy ME et al. *JAMA Neurology.*

(3U01AG052410), Estudio Familiar de Influencia Genetica en Alzheimer (EFIGA) (5R37AG015473, RF1AG015473, R56AG051876), Genetic and Environmental Risk Factors for Alzheimer Disease Among African Americans Study (GenerAAtions) (2R01AG09029, R01AG025259, 2R01AG048927), Gwangju Alzheimer and Related Dementias Study (GARD) (U01AG062602), Hussman Institute for Human Genomics Brain Bank (HIHGBB) (R01AG027944, Alzheimer's Association "Identification of Rare Variants in Alzheimer Disease"), Ibadan Study of Aging (IBADAN) (5R01AG009956), Mexican Health and Aging Study (MHAS) (R01AG018016), Multi-Institutional Research in Alzheimer's Genetic Epidemiology (MIRAGE) (2R01AG09029, R01AG025259, 2R01AG048927), Northern Manhattan Study (NOMAS) (R01NS29993), Peru Alzheimer's Disease Initiative (PeADI) (RF1AG054074), Puerto Rican 1066 (PR1066) (Wellcome Trust (GR066133/GR080002), European Research Council (340755)), Puerto Rican Alzheimer Disease Initiative (PRADI) (RF1AG054074), Reasons for Geographic and Racial Differences in Stroke (REGARDS) (U01NS041588), Research in African American Alzheimer Disease Initiative (REAAADI) (U01AG052410), Rush Alzheimer's Disease Center (ROSMAP) (P30AG10161, R01AG15819, R01AG17919), University of Miami Brain Endowment Bank (MBB), and University of Miami/Case Western/North Carolina A&T African American (UM/CASE/NCAT) (U01AG052410, R01AG028786). The four LSACs are: the Human Genome Sequencing Center at the Baylor College of Medicine (U54 HG003273), the Broad Institute Genome Center (U54HG003067), The American Genome Center at the Uniformed Services University of the Health Sciences (U01AG057659), and the Washington University Genome Institute (U54HG003079). Biological samples and associated phenotypic data used in primary data analyses were stored at Study Investigators institutions, and at the National Cell Repository for Alzheimer's Disease (NCRAD, U24AG021886) at Indiana University funded by NIA. Associated Phenotypic Data used in primary and secondary data analyses were provided by Study Investigators, the NIA funded Alzheimer's Disease Centers (ADCs), and the National Alzheimer's Coordinating Center (NACC, U01AG016976) and the National Institute on Aging Genetics of Alzheimer's Disease Data Storage Site (NIAGADS, U24AG041689) at the University of Pennsylvania, funded by NIA This research was supported in part by the Intramural Research Program of the National Institutes of health, National Library of Medicine. Contributors to the Genetic Analysis Data included Study Investigators on projects that were individually funded by NIA, and other NIH institutes, and by private U.S. organizations, or foreign governmental or nongovernmental organizations. An up to date acknowledgment statement can be found on the ADSP site: https://www.niagads.org/adsp/content/acknowledgement-statement. Data collection and sharing for this project was funded by the Alzheimer's Disease Neuroimaging Initiative (ADNI) (National Institutes of Health Grant U01 AG024904) and DOD ADNI (Department of Defense

© 2023 Belloy ME et al. *JAMA Neurology.*

award number W81XWH-12-2-0012). ADNI is funded by the National Institute on Aging, the National Institute of Biomedical Imaging and Bioengineering, and through generous contributions from the following: AbbVie, Alzheimer's Association; Alzheimer's Drug Discovery Foundation; Araclon Biotech; BioClinica, Inc.; Biogen; Bristol-Myers Squibb Company; CereSpir, Inc.; Cogstate; Eisai Inc.; Elan Pharmaceuticals, Inc.; Eli Lilly and Company; EuroImmun; F. Hoffmann-La Roche Ltd and its affiliated company Genentech, Inc.; Fujirebio; GE Healthcare; IXICO Ltd; Janssen Alzheimer Immunotherapy Research & Development, LLC.; Johnson & Johnson Pharmaceutical Research & Development LLC.; Lumosity; Lundbeck; Merck & Co., Inc.; Meso Scale Diagnostics, LLC.; NeuroRx Research; Neurotrack Technologies; Novartis Pharmaceuticals Corporation; Pfizer Inc.; Piramal Imaging; Servier; Takeda Pharmaceutical Company; and Transition Therapeutics. The Canadian Institutes of Health Research is providing funds to support ADNI clinical sites in Canada. Private sector contributions are facilitated by the Foundation for the National Institutes of Health (www.fnih.org). The grantee organization is the Northern California Institute for Research and Education, and the study is coordinated by the Alzheimer's Therapeutic Research Institute at the University of Southern California. ADNI data are disseminated by the Laboratory for Neuro Imaging at the University of Southern California. Additional information to include in an acknowledgment statement can be found on the LONI site: https://adni.loni.usc.edu/wp-content/uploads/how_to_apply/ADNI_Data_Use_Agreement.pdf. The Alzheimer's Disease Genetics Consortium (ADGC) supported sample preparation, whole exome sequencing and data processing through NIA grant U01AG032984. Sequencing data generation and harmonization is supported by the Genome Center for Alzheimer's Disease, U54AG052427, and data sharing is supported by NIAGADS, U24AG041689. Samples from the National Centralized Repository for Alzheimer's Disease and Related Dementias (NCRAD), which receives government support under a cooperative agreement grant (U24 AG021886) awarded by the National Institute on Aging (NIA), were used in this study. We thank contributors who collected samples used in this study, as well as patients and their families, whose help and participation made this work possible. NIH grants supported enrollment and data collection for the individual studies including: GenerAAtions R01AG20688 (PI M. Daniele Fallin, PhD); Miami/Duke R01 AG027944, R01 AG028786 (PI Margaret A. Pericak-Vance, PhD); NC A&T P20 MD000546, R01 AG28786-01A1 (PI Goldie S. Byrd, PhD); Case Western (PI Jonathan L. Haines, PhD); MIRAGE R01 AG009029 (PI Lindsay A. Farrer, PhD); ROS P30AG10161, R01AG15819, R01AG30146, TGen (PI David A. Bennett, MD); MAP R01AG17917, R01AG15819, TGen (PI David A. Bennett, MD). The NACC database is funded by NIA/NIH Grant U01 AG016976. NACC data are contributed by the NIA-funded ADCs: P30 AG019610 (PI Eric Reiman, MD), P30 AG013846 (PI Neil

© 2023 Belloy ME et al. *JAMA Neurology.*

Kowall, MD), P30 AG062428-01 (PI James Leverenz, MD) P50 AG008702 (PI Scott Small, MD), P50 AG025688 (PI Allan Levey, MD, PhD), P50 AG047266 (PI Todd Golde, MD, PhD), P30 AG010133 (PI Andrew Saykin, PsyD), P50 AG005146 (PI Marilyn Albert, PhD), P30 AG062421-01 (PI Bradley Hyman, MD, PhD), P30 AG062422-01 (PI Ronald Petersen, MD, PhD), P50 AG005138 (PI Mary Sano, PhD), P30 AG008051 (PI Thomas Wisniewski, MD), P30 AG013854 (PI Robert Vassar, PhD), P30 AG008017 (PI Jeffrey Kaye, MD), P30 AG010161 (PI David Bennett, MD), P50 AG047366 (PI Victor Henderson, MD, MS), P30 AG010129 (PI Charles DeCarli, MD), P50 AG016573 (PI Frank LaFerla, PhD), P30 AG062429-01(PI James Brewer, MD, PhD), P50 AG023501 (PI Bruce Miller, MD), P30 AG035982 (PI Russell Swerdlow, MD), P30 AG028383 (PI Linda Van Eldik, PhD), P30 AG053760 (PI Henry Paulson, MD, PhD), P30 AG010124 (PI John Trojanowski, MD, PhD), P50 AG005133 (PI Oscar Lopez, MD), P50 AG005142 (PI Helena Chui, MD), P30 AG012300 (PI Roger Rosenberg, MD), P30 AG049638 (PI Suzanne Craft, PhD), P50 AG005136 (PI Thomas Grabowski, MD), P30 AG062715-01 (PI Sanjay Asthana, MD, FRCP), P50 AG005681 (PI John Morris, MD), P50 AG047270 (PI Stephen Strittmatter, MD, PhD). This work was supported by grants from the National Institutes of Health (R01AG044546, P01AG003991, RF1AG053303, R01AG058501, U01AG058922, RF1AG058501 and R01AG057777). The recruitment and clinical characterization of research participants at Washington University were supported by NIH P50 AG05681, P01 AG03991, and P01 AG026276. This work was supported by access to equipment made possible by the Hope Center for Neurological Disorders, and the Departments of Neurology and Psychiatry at Washington University School of Medicine. We thank the contributors who collected samples used in this study, as well as patients and their families, whose help and participation made this work possible. Members of the National Institute on Aging Late-Onset Alzheimer Disease/National Cell Repository for Alzheimer Disease (NIA-LOAD NCRAD) Family Study Group include the following: Richard Mayeux, MD, MSc; Martin Farlow, MD; Tatiana Foroud, PhD; Kelley Faber, MS; Bradley F. Boeve, MD; Neill R. Graff-Radford, MD; David A. Bennett, MD; Robert A. Sweet, MD; Roger Rosenberg, MD; Thomas D. Bird, MD; Carlos Cruchaga, PhD; and Jeremy M. Silverman, PhD. This work was partially supported by grant funding from NIH R01 AG039700 and NIH P50 AG005136. Participants and samples used here were originally collected with grant funding from NIH U24 AG026395, U24 AG021886, P50 AG008702, P01 AG007232, R37 AG015473, P30 AG028377, P50 AG05128, P50 AG16574, P30 AG010133, P50 AG005681, P01 AG003991, U01MH046281, U01 MH046290 and U01 MH046373. The funders had no role in study design, analysis or preparation of the manuscript. The authors declare no competing interests. This work was supported by the National Institutes of Health (R01 AG027944, R01 AG028786 to MAPV, R01 AG019085 to JLH, P20 MD000546); a joint grant from the Alzheimer's Association (SG-14-

© 2023 Belloy ME et al. *JAMA Neurology.*

312644) and the Fidelity Biosciences Research Initiative to MAPV; the BrightFocus Foundation (A2011048 to MAPV). NIA-LOAD Family-Based Study supported the collection of samples used in this study through NIH grants U24 AG026395 and R01 AG041797 and the MIRAGE cohort was supported through the NIH grants R01 AG025259 and R01 AG048927. We thank contributors, including the Alzheimer's disease Centers who collected samples used in this study, as well as patients and their families, whose help and participation made this work possible. Study design: HNC, BWK, JLH, MAPV; Sample collection: MLC, JMV, RMC, LAF, JLH, MAPV; Whole exome sequencing and Sanger sequencing: SR, PLW; Sequencing data analysis: HNC, BWK, KLHN, SR, MAK, JRG, ERM, GWB, MAPV; Statistical analysis: BWK, KLHN, JMJ, MAPV; Preparation of manuscript: HNC, BWK. The authors jointly discussed the experimental results throughout the duration of the study. All authors read and approved the final manuscript. Data collection and sharing for this project was supported by the Washington Heights-Inwood Columbia Aging Project (WHICAP, PO1AG07232, R01AG037212, RF1AG054023) funded by the National Institute on Aging (NIA) and by the National Center for Advancing Translational Sciences, National Institutes of Health, through Grant Number UL1TR001873. This manuscript has been reviewed by WHICAP investigators for scientific content and consistency of data interpretation with previous WHICAP Study publications. We acknowledge the WHICAP study participants and the WHICAP research and support staff for their contributions to this study. This work was supported by grants from the National Institutes of Health (R01AG044546, P01AG003991, RF1AG053303, R01AG058501, U01AG058922, RF1AG058501 and R01AG057777). The recruitment and clinical characterization of research participants at Washington University were supported by NIH P50 AG05681, P01 AG03991, and P01 AG026276. This work was supported by access to equipment made possible by the Hope Center for Neurological Disorders, and the Departments of Neurology and Psychiatry at Washington University School of Medicine. We thank the contributors who collected samples used in this study, as well as patients and their families, whose help and participation made this work possible. Members of the National Institute on Aging Late-Onset Alzheimer Disease/National Cell Repository for Alzheimer Disease (NIA-LOAD NCRAD) Family Study Group include the following: Richard Mayeux, MD, MSc; Martin Farlow, MD; Tatiana Foroud, PhD; Kelley Faber, MS; Bradley F. Boeve, MD; Neill R. Graff-Radford, MD; David A. Bennett, MD; Robert A. Sweet, MD; Roger Rosenberg, MD; Thomas D. Bird, MD; Carlos Cruchaga, PhD; and Jeremy M. Silverman, PhD. This work was supported by grants from the National Institutes of Health (R01AG044546, P01AG003991, RF1AG053303, R01AG058501, U01AG058922, RF1AG058501 and R01AG057777). The recruitment and clinical characterization of research participants at Washington University were supported by NIH P50 AG05681, P01 AG03991, and P01 AG026276. This work was

© 2023 Belloy ME et al. *JAMA Neurology.*

supported by access to equipment made possible by the Hope Center for Neurological Disorders, and the Departments of Neurology and Psychiatry at Washington University School of Medicine. We thank the contributors who collected samples used in this study, as well as patients and their families, whose help and participation made this work possible. Members of the National Institute on Aging Late-Onset Alzheimer Disease/National Cell Repository for Alzheimer Disease (NIA-LOAD NCRAD) Family Study Group include the following: Richard Mayeux, MD, MSc; Martin Farlow, MD; Tatiana Foroud, PhD; Kelley Faber, MS; Bradley F. Boeve, MD; Neill R. Graff-Radford, MD; David A. Bennett, MD; Robert A. Sweet, MD; Roger Rosenberg, MD; Thomas D. Bird, MD; Carlos Cruchaga, PhD; and Jeremy M. Silverman, PhD. Mayo RNaseq Study- Study data were provided by the following sources: The Mayo Clinic Alzheimer's Disease Genetic Studies, led by Dr Nilufer Ertekin-Taner and Dr Steven G. Younkin, Mayo Clinic, Jacksonville, FL using samples from the Mayo Clinic Study of Aging, the Mayo Clinic Alzheimer's Disease Research Center, and the Mayo Clinic Brain Bank. Data collection was supported through funding by NIA grants P50 AG016574, R01 AG032990, U01 AG046139, R01 AG018023, U01 AG006576, U01 AG006786, R01 AG025711, R01 AG017216, R01 AG003949, NINDS grant R01 NS080820, CurePSP Foundation, and support from Mayo Foundation. Study data includes samples collected through the Sun Health Research Institute Brain and Body Donation Program of Sun City, Arizona. The Brain and Body Donation Program is supported by the National Institute of Neurological Disorders and Stroke (U24 NS072026 National Brain and Tissue Resource for Parkinson's Disease and Related Disorders), the National Institute on Aging (P30 AG19610 Arizona Alzheimer's Disease Core Center), the Arizona Department of Health Services (contract 211002, Arizona Alzheimer's Research Center), the Arizona Biomedical Research Commission (contracts 4001, 0011, 05-901 and 1001 to the Arizona Parkinson's Disease Consortium) and the Michael J. Fox Foundation for Parkinson's Research ROSMAP- We are grateful to the participants in the Religious Order Study, the Memory and Aging Project. This work is supported by the US National Institutes of Health [U01 AG046152, R01 AG043617, R01 AG042210, R01 AG036042, R01 AG036836, R01 AG032990, R01 AG18023, RC2 AG036547, P50 AG016574, U01 ES017155, KL2 RR024151, K25 AG041906-01, R01 AG30146, P30 AG10161, R01 AG17917, R01 AG15819, K08 AG034290, P30 AG10161 and R01 AG11101. Mount Sinai Brain Bank (MSBB)- This work was supported by the grants R01AG046170, RF1AG054014, RF1AG057440 and R01AG057907 from the NIH/National Institute on Aging (NIA). R01AG046170 is a component of the AMP-AD Target Discovery and Preclinical Validation Project. Brain tissue collection and characterization was supported by NIH HHSN271201300031C. This study was supported by the National Institute on Aging (NIA) grants AG030653, AG041718, AG064877 and P30-AG066468. We would like to thank study participants, their

© 2023 Belloy ME et al. *JAMA Neurology.*

families, and the sample collectors for their invaluable contributions. This research was supported in part by the National Institute on Aging grant U01AG049508 (PI Alison M. Goate). This research was supported in part by Genentech, Inc. (PI Alison M. Goate, Robert R. Graham). The NACC database is funded by NIA/NIH Grant U01 AG016976. NACC data are contributed by these NIA-funded ADCs: P30 AG013846 (PI Neil Kowall, MD), P50 AG008702 (PI Scott Small, MD), P50 AG025688 (PI Allan Levey, MD, PhD), P30 AG010133 (PI Andrew Saykin, PsyD), P50 AG005146 (PI Marilyn Albert, PhD), P50 AG005134 (PI Bradley Hyman, MD, PhD), P50 AG016574 (PI Ronald Petersen, MD, PhD), P30 AG013854 (PI M. Marsel Mesulam, MD), P30 AG008017 (PI Jeffrey Kaye, MD), P30 AG010161 (PI David Bennett, MD), P30 AG010129 (PI Charles DeCarli, MD), P50 AG016573 (PI Frank LaFerla, PhD), P50 AG005131 (PI Douglas Galasko, MD), P30 AG028383 (PI Linda Van Eldik, PhD), P30 AG010124 (PI John Trojanowski, MD, PhD), P50 AG005142 (PI Helena Chui, MD), P30 AG012300 (PI Roger Rosenberg, MD), P50 AG005136 (PI Thomas Grabowski, MD), P50 AG005681 (PI John Morris, MD), P30 AG028377 (Kathleen Welsh-Bohmer, PhD), and P50 AG008671 (PI Henry Paulson, MD, PhD). Samples from the National Cell Repository for Alzheimer's Disease (NCRAD), which receives government support under a cooperative agreement grant (U24 AG21886) awarded by the National Institute on Aging (NIA), were used in this study. We thank contributors who collected samples used in this study, as well as patients and their families, whose help and participation made this work possible. The Alzheimer's Disease Genetics Consortium supported the collection of samples used in this study through National Institute on Aging (NIA) grants U01AG032984 and RC2AG036528. We acknowledge the generous contributions of the Cache County Memory Study participants. Sequencing for this study was funded by RF1AG054052 (PI: John S.K. Kauwe) Acknowledgments for the use of GWAS data distributed by NIAGADS: The NIA Genetics of Alzheimer's Disease Data Storage Site (NIAGADS) is supported by a collaborative agreement from the National Institute on Aging, U24AG041689. NG00047: The NIA supported this work through grants U01-AG032984, RC2-AG036528, U01-AG016976 (Dr Kukull); U24 AG026395, U24 AG026390, R01AG037212, R37 AG015473 (Dr Mayeux); K23AG034550 (Dr Reitz); U24-AG021886 (Dr Foroud); R01AG009956, RC2 AG036650 (Dr Hall); UO1 AG06781, UO1 HG004610 (Dr Larson); R01 AG009029 (Dr Farrer); 5R01AG20688 (Dr Fallin); P50 AG005133, AG030653 (Dr Kamboh); R01 AG019085 (Dr Haines); R01 AG1101, R01 AG030146, RC2 AG036650 (Dr Evans); P30AG10161, R01AG15819, R01AG30146, R01AG17917, R01AG15819 (Dr Bennett); R01AG028786 (Dr Manly); R01AG22018, P30AG10161 (Dr Barnes); P50AG16574 (Dr Ertekin-Taner, Dr Graff-Radford), R01 AG032990 (Dr Ertekin-Taner), KL2 RR024151 (Dr Ertekin-Taner); R01 AG027944, R01 AG028786 (Dr Pericak-Vance); P20 MD000546, R01 AG28786-01A1 (Dr Byrd); AG005138 (Dr Buxbaum); P50 AG05681, P01 AG03991, P01 AG026276 (Dr

Goate); and P30AG019610, P30AG13846, U01-AG10483, R01CA129769, R01MH080295, R01AG017173, R01AG025259, R01AG33193, P50AG008702, P30AG028377, AG05128, AG025688, P30AG10133, P50AG005146, P50AG005134, P01AG002219, P30AG08051, MO1RR00096, UL1RR029893, P30AG013854, P30AG008017, R01AG026916, R01AG019085, P50AG016582, UL1RR02777, R01AG031581, P30AG010129, P50AG016573, P50AG016575, P50AG016576, P50AG016577, P50AG016570, P50AG005131, P50AG023501, P50AG019724, P30AG028383, P50AG008671, P30AG010124, P50AG005142, P30AG012300, AG010491, AG027944, AG021547, AG019757, P50AG005136 (Alzheimer Disease GeneticsConsortium [ADGC]). We thank Creighton Phelps, Stephen Synder, and Marilyn Miller from the NIA, who are ex-officio members of the ADGC. Support was also provided by the Alzheimer's Association (IIRG-08-89720 [Dr Farrer] and IIRG-05-14147 [Dr Pericak-Vance]), National Institute of Neurological Disorders and Stroke grant NS39764, National Institute of Mental Health grant MH60451, GlaxoSmithKline, and the Office of Research and Development, Biomedical Laboratory Research Program, US Department of Veterans Affairs Administration. For the ADGC, biological samples and associated phenotypic data used in primary data analyses were stored at principal investigators' institutions and at the National Cell Repository for Alzheimer's Disease (NCRAD) at Indiana University, funded by the NIA. Associated phenotypic data used in secondary data analyses were stored at the National Alzheimer's Coordinating Center and at the NIA Alzheimer's Disease Data Storage Site at the University of Pennsylvania, funded by the NIA. Contributors to the genetic analysis data included principal investigators on projects ndividually funded by the NIA, other NIH institutes, or private entities. Acknowledgments for other GWAS and phenotype data: NACC: The NACC database is funded by NIA/NIH Grant U01 AG016976. NACC data are contributed by the NIA-funded ADCs: P30 AG019610 (PI Eric Reiman, MD), P30 AG013846 (PI Neil Kowall, MD), P30 AG062428-01 (PI James Leverenz, MD) P50 AG008702 (PI Scott Small, MD), P50 AG025688 (PI Allan Levey, MD, PhD), P50 AG047266 (PI Todd Golde, MD, PhD), P30 AG010133 (PI Andrew Saykin, PsyD), P50 AG005146 (PI Marilyn Albert, PhD), P30 AG062421-01 (PI Bradley Hyman, MD, PhD), P30 AG062422-01 (PI Ronald Petersen, MD, PhD), P50 AG005138 (PI Mary Sano, PhD), P30 AG008051 (PI Thomas Wisniewski, MD), P30 AG013854 (PI Robert Vassar, PhD), P30 AG008017 (PI Jeffrey Kaye, MD), P30 AG010161 (PI David Bennett, MD), P50 AG047366 (PI Victor Henderson, MD, MS), P30 AG010129 (PI Charles DeCarli, MD), P50 AG016573 (PI Frank LaFerla, PhD), P30 AG062429-01(PI James Brewer, MD, PhD), P50 AG023501 (PI Bruce Miller, MD), P30 AG035982 (PI Russell Swerdlow, MD), P30 AG028383 (PI Linda Van Eldik, PhD), P30 AG053760 (PI Henry Paulson, MD, PhD), P30 AG010124 (PI John Trojanowski, MD, PhD), P50 AG005133 (PI Oscar Lopez, MD), P50 AG005142 (PI Helena Chui, MD), P30 AG012300 (PI Roger

© 2023 Belloy ME et al. *JAMA Neurology.*

Rosenberg, MD), P30 AG049638 (PI Suzanne Craft, PhD), P50 AG005136 (PI Thomas Grabowski, MD), P30 AG062715-01 (PI Sanjay Asthana, MD, FRCP), P50 AG005681 (PI John Morris, MD), P50 AG047270 (PI Stephen Strittmatter, MD, PhD). MARS & LATC: We thank all Minority Aging Research Study and Latino Core participants and the Rush Alzheimer's Disease Center staff. This database was funded by the NIH/NIA grants R01AG22018 (MARS) and P30AG 072975 (ADC). GenADA: The genotypic and associated phenotypic data used in the study "Multi-Site Collaborative Study for Genotype-Phenotype Associations in Alzheimer's Disease (GenADA)" were provided by the GlaxoSmithKline, R&D Limited. ROSMAP: ROSMAP study data were provided by the Rush Alzheimer's Disease Center, Rush University Medical Center, Chicago. Data collection was supported through funding by NIA grants P30AG10161, R01AG15819, R01AG17917, R01AG30146, R01AG36836, U01AG32984, U01AG46152, the Illinois Department of Public Health, and the Translational Genomics Research Institute. AddNeuroMed: The AddNeuroMed data are from a public-private partnership supported by EFPIA companies and SMEs as part of InnoMed (Innovative Medicines in Europe), an Integrated Project funded by the European Union of the Sixth Framework program priority FP6-2004-LIFESCIHEALTH-5. Clinical leads responsible for data collection are Iwona Kłoszewska (Lodz), Simon Lovestone (London), Patrizia Mecocci (Perugia), Hilkka Soininen (Kuopio), Magda Tsolaki (Thessaloniki), and Bruno Vellas (Toulouse), imaging leads are Andy Simmons (London), Lars-Olad Wahlund (Stockholm) and Christian Spenger (Zurich) and bioinformatics leads are Richard Dobson (London) and Stephen Newhouse (London). ADNI: Data collection and sharing for this project was funded by the Alzheimer's Disease Neuroimaging Initiative (ADNI) (National Institutes of Health Grant U01 AG024904) and DOD ADNI (Department of Defense award number W81XWH-12-2-0012). ADNI is funded by the National Institute on Aging, the National Institute of Biomedical Imaging and Bioengineering and through generous contributions from the following: AbbVie. Alzheimer's Association; Alzheimer's Drug Discovery Foundation; Araclon Biotech; BioClinica. Inc.; Biogen; Bristol-Myers Squibb Company; CereSpir. Inc.; Cogstate; Eisai Inc.; Elan Pharmaceuticals. Inc.; Eli Lilly and Company; EuroImmun; F. Hoffmann-La Roche Ltd and its affiliated company Genentech. Inc.; Fujirebio; GE HealtControlsare; IXICO Ltd; Janssen Alzheimer Immunotherapy Research & Development. LLC.; Johnson & Johnson Pharmaceutical Research & Development LLC.; Lumosity; Lundbeck; Merck & Co. Inc.; Meso Scale Diagnostics. LLC.; NeuroRx Research; Neurotrack Technologies; Novartis Pharmaceuticals Corporation; Pfizer Inc.; Piramal Imaging; Servier; Takeda Pharmaceutical Company; and Transition Therapeutics. The Canadian Institutes of Health Research is providing funds to support ADNI clinical sites in Canada. Private sector contributions are facilitated by the Foundation for the National Institutes of Health. The grantee organization is the Northern California Institute for Research

© 2023 Belloy ME et al. *JAMA Neurology.*

and Education, and the study is coordinated by the Alzheimer's Therapeutic Research Institute at the University of Southern California. ADNI data are disseminated by the Laboratory for Neuro Imaging at the University of Southern California. NCRAD: Biological samples used in this study were stored at study investigators' institutions and at the National Cell Repository for Alzheimer's Disease (NCRAD) at Indiana University, which receives government support under a cooperative agreement grant (U24 AG21886) awarded by the National Institute on Aging (NIA).

© 2023 Belloy ME et al. *JAMA Neurology.*

eFigure 1. Flow Chart of Sample/Participant Filtering for *APOE* Association Analyses With Alzheimer's Disease Risk

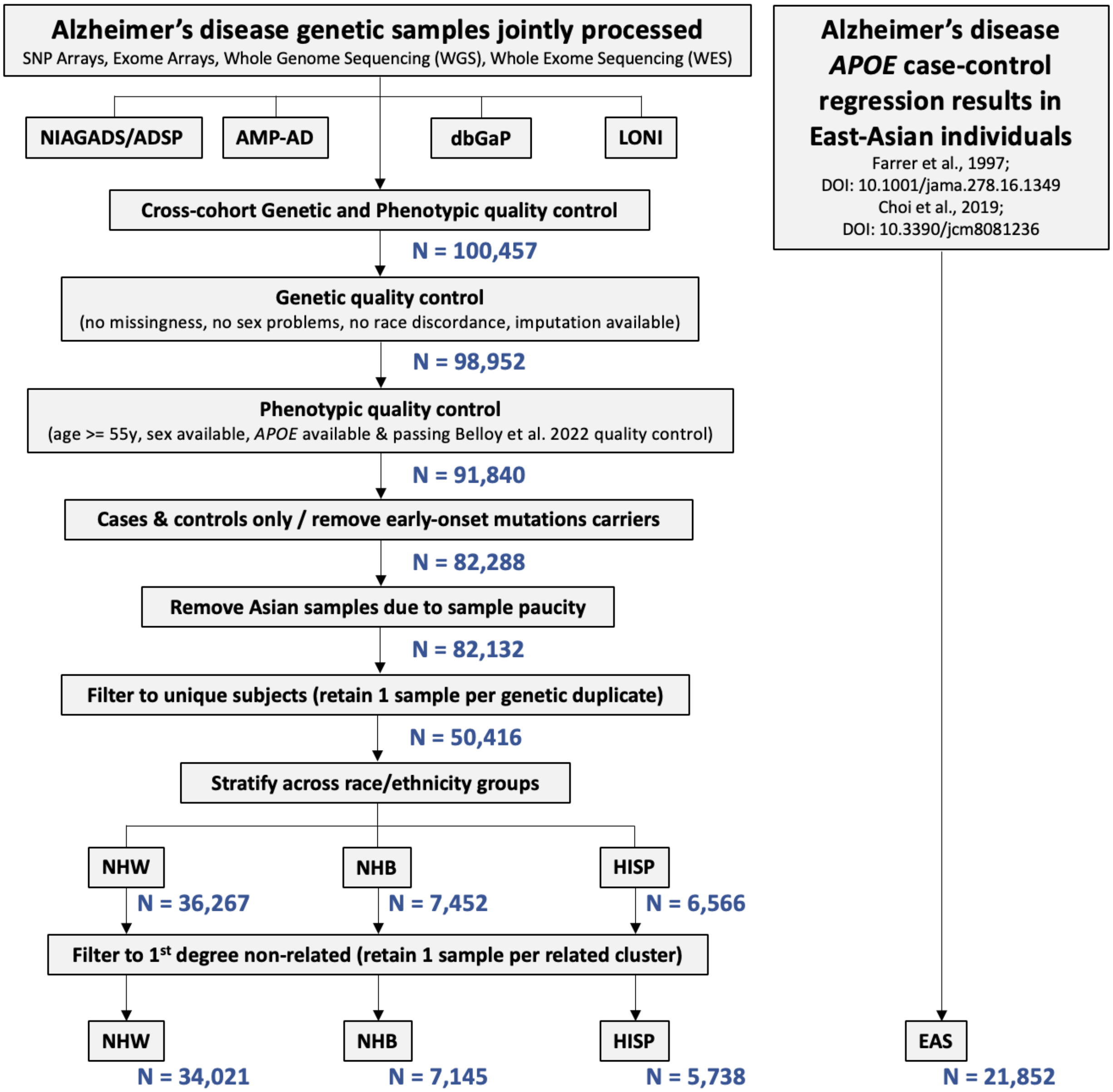

The Belloy et al. 2022 quality control procedure is described in the supplementary methods and in the original study.[37]

© 2023 Belloy ME et al. *JAMA Neurology.*

eFigure 2. Admixture Plots Across the Five Major Super Populations, for Participants Included in Association Analyses

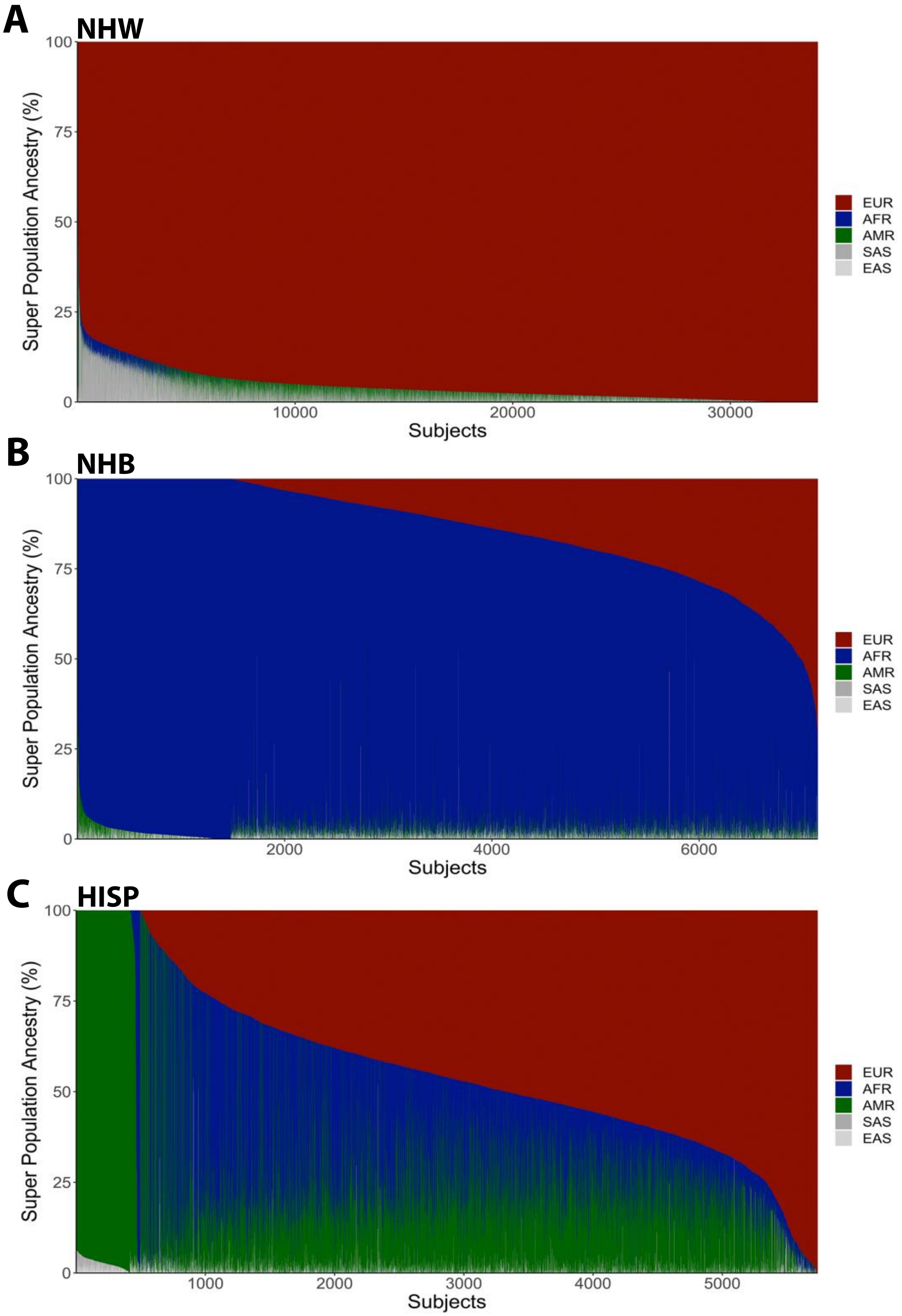

(A) Non-Hispanic White (NHW), (B) Non-Hispanic Black (NHB), (C) Hispanic (HISP).

Abbreviations: EUR, European; AFR, African; AMR; Amerindian; SAS, South Asian; EAS; East Asian.

© 2023 Belloy ME et al. *JAMA Neurology.*

eFigure 3. Alternative Visualizations of Case-Control Regression Results Presented in Figure 1

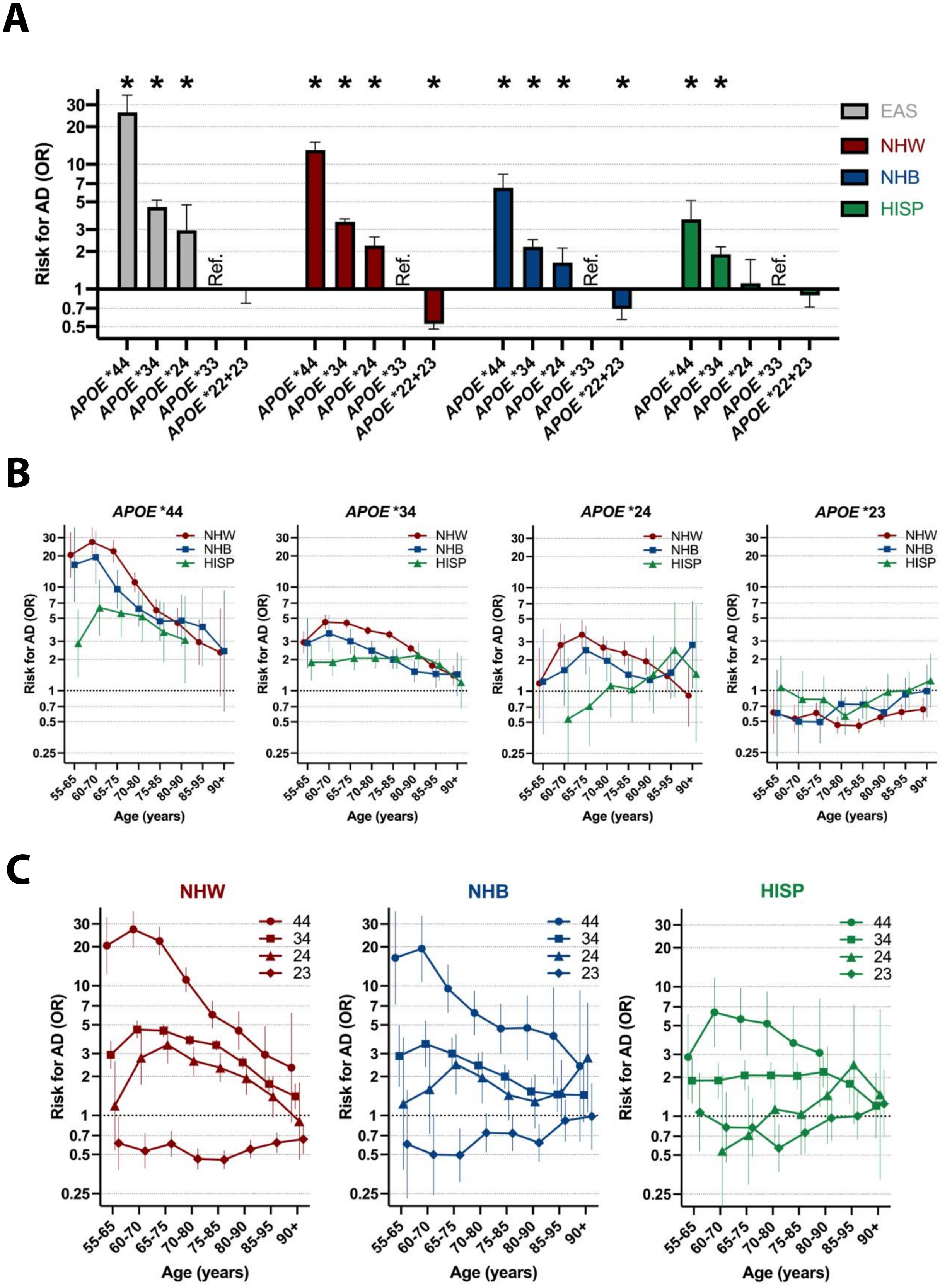

© 2023 Belloy ME et al. *JAMA Neurology.*

eFigure 4. Sex-and-Age Stratified Results for *APOE* Genotype Case-Control Regression Analyses Across Non-Hispanic White (NHW), Non-Hispanic Black (NHB), and Hispanic (HISP) Individuals

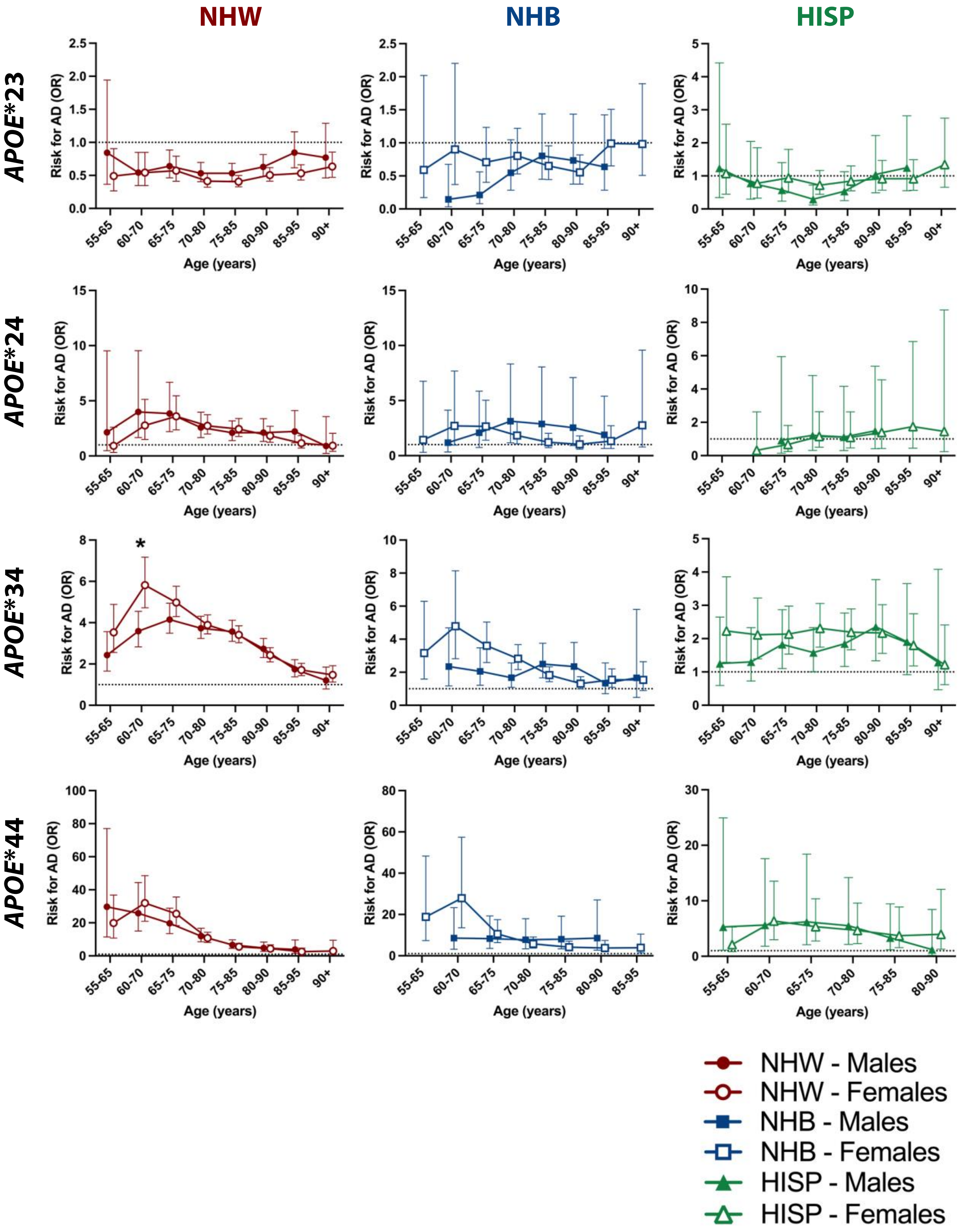

Star marks significant sex difference.

© 2023 Belloy ME et al. *JAMA Neurology.*

eFigure 5. *APOE**34-by-Sex Interaction Effect in Case-Control Regression Analyses for Individuals 60 to 70 Years of Age Is Preserved Across Sensitivity Analyses

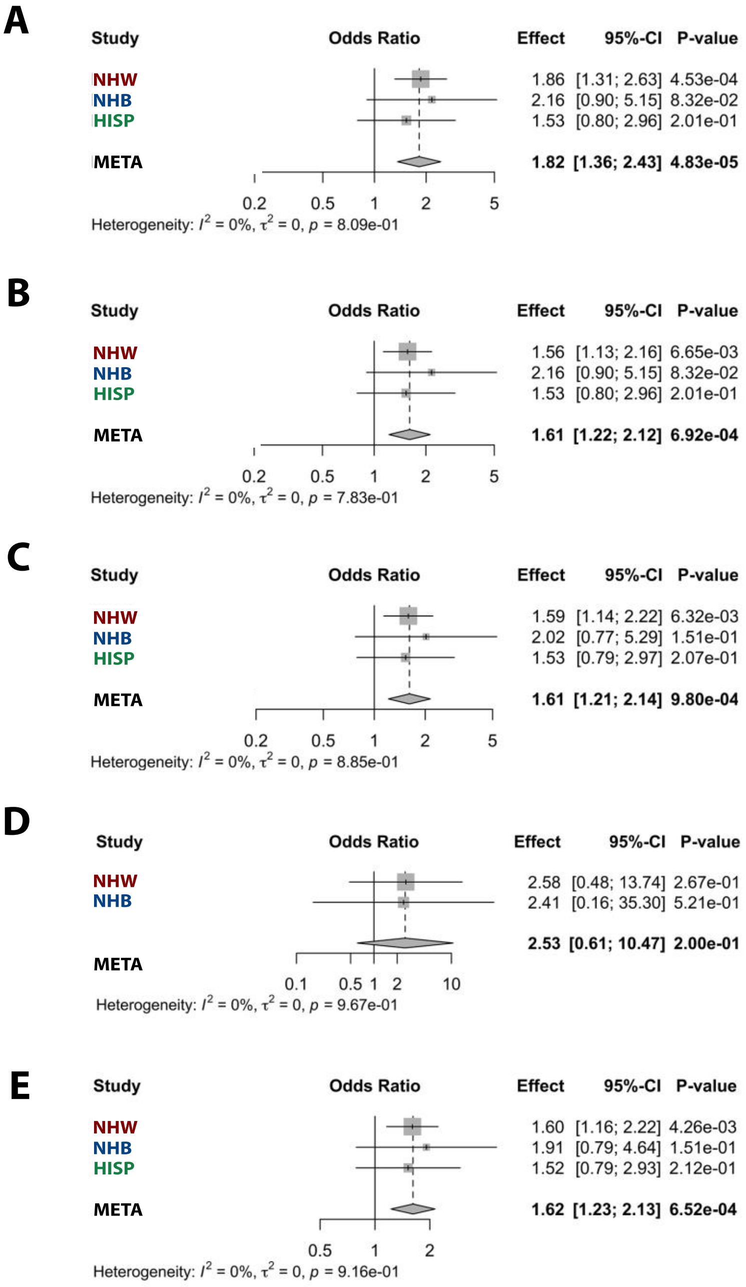

A) Excluding subjects with pathology. B) Additionally adjusting for pathology verification status. C) Using only samples from cohorts with a clinical/hospital or autopsy/brainbank ascertainment design, while additionally adjusting for pathology verification status. D) Using only samples from cohorts with a community/population-based ascertainment design, while additionally adjusting for pathology verification status (HISP not included due to sample paucity). E) Additionally adjusting for ascertainment design and pathology verification status.

© 2023 Belloy ME et al. *JAMA Neurology.*

eFigure 6. Survival Analyses Results, Through Cox Regression, Across *APOE* Genotypes, for Non-Hispanic White (NHW), Non-Hispanic Black (NHB), and Hispanic (HISP) Individuals

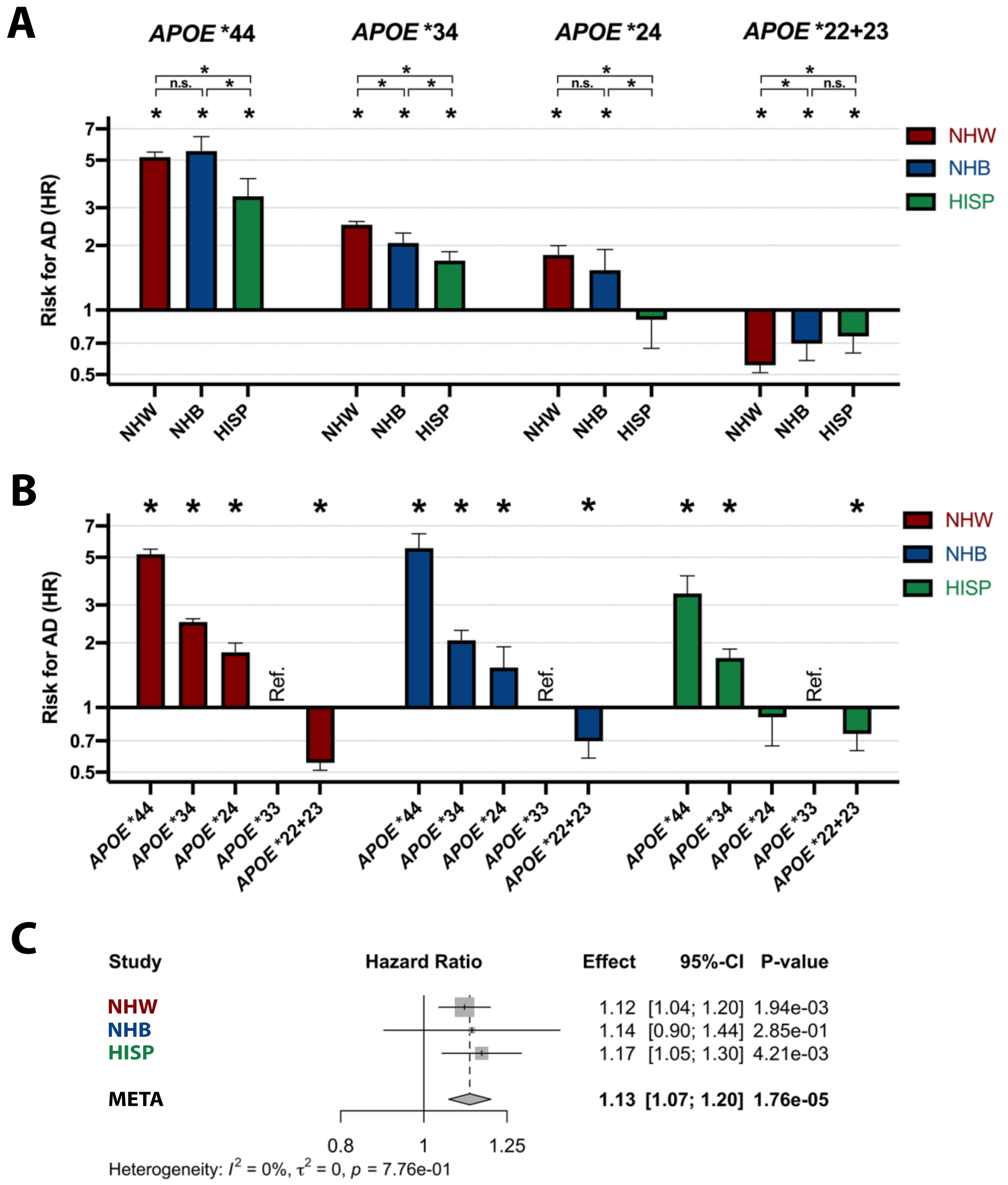

For exact summary statistics corresponding to this figure, please cf. eTable 13. **(A-B)** Compared to case-control regression results presented in Figure 1A, the current figure shows more pronounced *APOE**22+23 effects in HISP individuals and loss of *APOE**24 and *APOE**44 significant differences across NHW and NHB. (C) In line with results present in Figure 1C, the *APOE**34-by-sex interaction was consistent across race/ethnicity groups and was even more significant upon meta-analysis compared the outcome of case-control regression analyses.

© 2023 Belloy ME et al. *JAMA Neurology.*

eTable 1. Overview of Genotyping Platforms Across All Available AD-Related Genetic Data

Cohort/Study	Genotyping Platform	Cohort-Platform ID	Sample count	Data Repository
ACT	Illumina Human 660W-Quad	ACT	2790	NIAGADS (NG00034) / dbGaP (phs000234)
ADC1	Illumina Human 660W-Quad	ADC1	2731	NIAGADS (NG00022) / NACC
ADC2	Illumina Human 660W-Quad	ADC2	928	NIAGADS (NG00023) / NACC
ADC3	Illumina Human OmniExpress	ADC3	1526	NIAGADS (NG00024) / NACC
ADC4	Illumina Human OmniExpress	ADC4	1054	NIAGADS (NG00068) / NACC
ADC5	Illumina Human OmniExpress	ADC5	1224	NIAGADS (NG00069) / NACC
ADC6	Illumina Human OmniExpress	ADC6	1333	NIAGADS (NG00070) / NACC
ADC7	Illumina Infinium Human OmniExpressExome	ADC7	1462	NIAGADS (NG00071) / NACC
ADDNEUROMED	Illumina Human 610-Quad	ADM_Q	315	Synapse AddNeuroMed (syn4907804)
	Illumina Human OmniExpress	ADM_O	329	Synapse AddNeuroMed (syn4907804)
ADNI	Illumina Human 610-Quad	ADNI_1	757	LONI ADNI
	Illumina Human OmniExpress	ADNI_2	361	LONI ADNI
	Illumina Global Screening Array (GSA)	ADNI_3	327	LONI ADNI
	Illumina Omni 2.5	ADNI_O25	812	LONI ADNI
	Whole Genome Sequencing - Illumina	ADNI_WGS	812	LONI ADNI
ADNI-DOD	Illumina Human OmniExpress	ADNI_DOD	204	LONI ADNIDOD
ADSP Exome-Arrays	Illumina HumanExome BeadChip v1.0 - CHOP	CHOP	5180	NIAGADS (NG00081) / NACC
	Illumina HumanExome BeadChip v1.0 - Miami	MIA	1923	NIAGADS (NG00080) / NACC
	Illumina HumanExome BeadChip v1.0 - Northshore	NS	5998	NIAGADS (NG00079) / NACC
	Illumina HumanExome BeadChip v1.0 - WashU	WU	868	NIAGADS (NG00085) / NACC
ADSP WES	Whole Exome Sequencing	ADSP_WES	20503	NIAGADS DSS (NG00067.v3) / NACC
ADSP WGS	Whole Genome Sequencing	ADSP_WGS	16906	NIAGADS DSS (NG00067.v5) / NACC
Indianapolis African-American	Illumina Human 1M-Duo	IIDP_AA	1175	NIAGADS (NG00047)
Indianapolis Yoruba	Illumina Omni 2.5	IIDP_YOR	1264	dbGaP (phs000378)
CIDR	Illumina Human Omni1-Quad	CIDR	3101	NIAGADS (NG00015) / dbGAP (phs000496)
GenADA	Affymetrix 500K	GSK	1571	dbGaP (phs000219)
HBTRC	Illumina Human Hap650Y	HBTRC_ILL	338	Synapse AMP-AD (syn3159435)
	Illumina Human Hap650Y	HBTRC_PERL	402	Synapse AMP-AD (syn3159435)

© 2023 Belloy ME et al. *JAMA Neurology.*

LATC	Illumina Multi-Ethnic – BU	LATC	63	RADC Rush (contact:Gregory_Klein@rush.edu)
NIA-LOAD	Illumina Human 610-Quad	LOAD	5220	NIAGADS (NG00020)
MARS	Illumina Multi-Ethnic – BU	MARS	708	RADC Rush (contact:Gregory_Klein@rush.edu)
MAYO	Illumina Human Hap300	MAYO_1	2099	Synapse AMP-AD (syn5591675) / NIAGADS (NG00029)
MAYO2	Illumina Omni 2.5	MAYO_2	314	Synapse AMP-AD (syn5550404)
MIRAGE	Illumina Human CNV370-Duo	MIRAGE_370	397	NIAGADS (NG00031)
	Illumina Human 610-Quad	MIRAGE_610	1105	NIAGADS (NG00031)
MTC	Illumina Human OmniExpress	MTC	542	NIAGADS (NG00096)
OHSU	Illumina Human CNV370-Duo	OHSU	647	NIAGADS (NG00017)
ROSMAP	Affymetrix GeneChip 6.0 - Broad Institute	ROSMAP_1B	1126	RADC Rush (contact:Gregory_Klein@rush.edu) / Synapse AMP-AD
	Affymetrix GeneChip 6.0 - TGen	ROSMAP_1T	582	RADC Rush (contact:Gregory_Klein@rush.edu) / Synapse AMP-AD
	Illumina Human OmniExpress 12 - Chop	ROSMAP_2C	382	RADC Rush (contact:Gregory_Klein@rush.edu) / Synapse AMP-AD
	Illumina Multi-Ethnic - BU	ROSMAP_3BU	494	RADC Rush (contact:Gregory_Klein@rush.edu)
TARCC	Affymetrix 6.0	TARCC	625	NIAGADS (NG00097)
	Illumina Multi-Ethnic – BU	TARCC_full	2718	TARCC (contact: Bruce.Jones@UTSouthwestern.edu)
TGEN2	Affymetrix 6.0	TGEN	1599	NIAGADS (NG00028)
UPITT	Illumina Human Omni1-Quad	UPITT	2440	NIAGADS (NG00026)
UM/VU/MSSM	Illumina Human 1M-Duo, Illumina 1M	UVM_A	1153	NIAGADS (NG00042)
	Affymetrix 6.0	UVM_B	864	NIAGADS (NG00042)
	Illumina Human 550K. Illumina Human 610-Quad	UVM_C	445	NIAGADS (NG00042)
WASHU	Illumina Human 610-Quad	WASHU_1	670	NIAGADS (NG00030)
WASHU2	Illumina Human OmniExpress	WASHU_2	235	NIAGADS (NG00087)
WHICAP	Illumina Human OmniExpress	WHICAP	647	NIAGADS (NG00093)

© 2023 Belloy ME et al. *JAMA Neurology.*

eTable 2. Overview of ADSP Studies With WES or WGS Available Through NIAGADS DSS (NG00067)

Study	Accession Number	Related Datasets
Accelerating Medicines Partnership- Alzheimer's Disease (AMP-AD)	sa000011	NG00067 – ADSP Umbrella
Cache County Study	sa000014	NG00067 – ADSP Umbrella
University of Pittsburgh- Kamboh WGS	sa000012	NG00067 – ADSP Umbrella
CurePSP and Tau Consortium PSP WGS	sa000016	NG00067 – ADSP Umbrella
NIH, CurePSP and Tau Consortium PSP WGS	sa000015	NG00067 – ADSP Umbrella
UCLA Progressive Supranuclear Palsy	sa000017	NG00067 – ADSP Umbrella
NACC Genentech WGS	sa000013	NG00067 – ADSP Umbrella
Alzheimer's Disease Sequencing Project (ADSP)	sa000001	NG00067 – ADSP Umbrella
Alzheimer's Disease Neuroimaging Initiative (ADNI)	sa000002	NG00067 – ADSP Umbrella
Alzheimer's Disease Genetics Consortium: African Americans (ADGC AA)	sa000003	NG00067 – ADSP Umbrella
The Familial Alzheimer Sequencing (FASe) project	sa000004	NG00067 – ADSP Umbrella
Brkanac – Family-based genome scan for AAO of LOAD	sa000005	NG00067 – ADSP Umbrella
HIHG Miami Families with AD	sa000006	NG00067 – ADSP Umbrella
Washington Heights/Inwood Columbia Aging Project (WHICAP)	sa000007	NG00067 – ADSP Umbrella
Charles F. and Joanne Knight Alzheimer's Disease Research Center (Knight ADRC)	sa000008	NG00067 – ADSP Umbrella
Corticobasal degeneration Study (CBD)	sa000009	NG00067 – ADSP Umbrella
Progressive Supranuclear Palsy Study (PSP)	sa000010	NG00067 – ADSP Umbrella

© 2023 Belloy ME et al. *JAMA Neurology.*

eTable 3. Overview of Participant Demographics Across Race and Ethnicity and *APOE* Genotype Strata

Group & *APOE* stratum		Diagnosis		Pathology		Sex	Age	Global Ancestry		
Name	Participants after QC (N)	Type	(N (%))	Available (N (%))	AD Path. (N (%))	Female (N (%))	Age (Mean (SD))	EUR% (Mean (SD))	AFR% (Mean (SD))	AMR% (Mean (SD))
**Non-Hispanic White (NHW)**										
**All**	34,021	CN	17,058 (50.1 %)	2,070 (12.1 %)	314 (15.2 %)	10,183 (59.7 %)	79.4 (8.8)	95.4 % (5.2 %)	0.6 % (1.9 %)	1.6 % (2.6 %)
		AD	16,963 (49.9 %)	6,639 (39.1 %)	6,639 (100 %)	9,982 (58.8 %)	74.5 (8.7)	95.6 % (4.8 %)	0.6 % (1.7 %)	1.6 % (2.5 %)
***APOE* *22**	133	CN	105 (78.9 %)	22 (21.0 %)	2 (9.1 %)	55 (52.4 %)	81.9 (8.3)	96.0 % (4.2 %)	0.6 % (1.5 %)	1.2 % (1.8 %)
		AD	28 (21.1 %)	9 (32.1 %)	9 (100 %)	13 (46.4 %)	79.1 (9.5)	95.8 % (4.0 %)	0.4 % (1.0 %)	1.6 % (1.7%)
***APOE* *23**	2,886	CN	2,185 (75.7 %)	317 (14.5 %)	44 (13.9 %)	1,329 (60.8 %)	80.8 (8.7)	95.5 % (5.2 %)	0.6 % (2.1 %)	1.6 % (2.3 %)
		AD	701 (24.3 %)	240 (34.2 %)	240 (100 %)	409 (58.3 %)	79.1 (9.5)	95.6 % (5.1 %)	0.6 % (1.7 %)	1.5 % (2.3 %)
***APOE* *33**	741	CN	10,647 (61.9 %)	1,349 (12.7 %)	202 (14.5 %)	6,345 (59.6 %)	79.8 (8.8)	95.2 % (5.2 %)	0.6 % (1.7 %)	1.6 % (2.6 %)
		AD	6,552 (38.1 %)	2,491 (38.0 %)	2,491 (100 %)	3,929 (60.0 %)	76.8 (9.3)	95.1 % (5.1 %)	0.7 % (1.6 %)	1.5 % (2.4 %)
***APOE* *24**	17,199	CN	319 (43.0 %)	32 (10.0 %)	4 (12.5 %)	204 (63.9 %)	78.7 (8.5)	95.5 % (6.8 %)	0.7 % (3.7 %)	1.8 % (4.8 %)
		AD	422 (57.0 %)	180 (42.7 %)	180 (100 %)	267 (63.3 %)	75.4 (7.5)	96.0 % (4.2 %)	0.5 % (2.0 %)	1.6 % (2.0 %)
***APOE* *34**	10,807	CN	3,533 (32.7 %)	333 (9.4 %)	57 (17.1 %)	2,084 (59.0 %)	77.7 (8.6)	95.7 % (5.0 %)	0.5 % (1.9 %)	1.6 % (2.4 %)
		AD	7,274 (67.3 %)	2,906 (40.0 %)	2,906 (100 %)	4,275 (58.8 %)	73.4 (7.6)	95.8 % (4.7 %)	0.5 % (1.7 %)	1.6 % (2.5 %)
***APOE* *44**	2,255	CN	269 (11.9 %)	17 (6.3 %)	5 (29.4 %)	166 (61.7 %)	74.7 (8.0)	96.1 % (6.6 %)	0.6 % (3.8 %)	1.8 % (3.2 %)
		AD	1,986 (88.1 %)	813 (40.9 %)	813 (100 %)	1,089 (54.8 %)	69.2 (6.8)	96.2 % (4.3 %)	0.4 % (1.9 %)	1.7 % (2.5 %)
**Non-Hispanic Black (NHB)**										
**All**	7,145	CN	5,134 (71.9 %)	38 (0.7 %)	5 (13.2 %)	3,627 (70.6 %)	79.2 (8.0)	13.8 % (13.9 %)	83.3 % (14.6 %)	1.6 % (2.8 %)
		AD	2,011 (28.1 %)	197 (9.8 %)	197 (100 %)	1,433 (71.3 %)	76.4 (8.4)	16.2 % (14.3 %)	80.8 % (14.7 %)	1.8 % (3.3 %)
***APOE* *22**	65	CN	55 (84.6 %)	0 (0.0 %)	-	37 (67.3 %)	80.0 (8.9)	11.9 % (15.1 %)	85.1 % (15.9 %)	1.5 % (2.3 %)
		AD	10 (15.4 %)	0 (0.0 %)	-	7 (70.0 %)	83.1 (5.6)	8.0 % (8.3 %)	89.1 % (8.6 %)	1.7 % (2.0 %)
***APOE* *23**	1,012	CN	854 (84.4 %)	7 (0.8 %)	0 (0.0 %)	581 (68.0 %)	79.4 (8.1)	14.5 % (13.9 %)	82.6 % (14.4 %)	1.6 % (3.2 %)
		AD	158 (15.6 %)	16 (10.1 %)	16 (100 %)	110 (69.6 %)	79.6 (8.2)	16.8 % (13.6 %)	80.1 % (15.0 %)	1.9 % (3.9 %)
***APOE* *33**	3,095	CN	2,464 (79.6 %)	21 (0.9 %)	3 (14.3 %)	1,762 (71.5 %)	79.7 (7.9)	14.1 % (14.5 %)	82.9 % (15.3 %)	1.6 % (2.7 %)
		AD	631 (20.4 %)	47 (7.4 %)	47 (100 %)	455 (72.1 %)	78.8 (8.1)	15.8 % (14.5 %)	81.2 % (14.9 %)	1.8 % (3.1 %)
***APOE* *24**	334	CN	231 (69.2 %)	5 (2.2 %)	0 (0.0 %)	164 (71.0 %)	78.6 (8.1)	12.8 % (13.4 %)	84.7 % (13.6 %)	1.4 % (2.0 %)
		AD	103 (30.8 %)	8 (7.8 %)	8 (100 %)	71 (68.9 %)	77.2 (8.1)	13.6 % (14.1 %)	82.9 % (15.1 %)	1.9 % (2.3 %)
***APOE* *34**	2,233	CN	1,391 (62.3 %)	5 (0.4 %)	2 (40.0 %)	978 (70.3 %)	78.6 (8.1)	13.2 % (13.0 %)	83.9 % (13.7 %)	1.7 % (3.0 %)
		AD	842 (37.7 %)	94 (11.2 %)	94 (100 %)	598 (71.0 %)	75.5 (8.1)	16.3 % (14.3 %)	80.7 % (14.6 %)	1.7 % (3.7 %)
***APOE* *44**	406	CN	139 (34.2 %)	0 (0 %)	-	105 (75.5 %)	76.3 (8.1)	13.3 % (12.2 %)	84.1 % (12.3 %)	1.4 % (2.2 %)
		AD	267 (65.8 %)	32 (12.0 %)	32 (100 %)	192 (71.9 %)	71.3 (7.7)	17.5 % (14.3 %)	79.6 % (14.5 %)	1.8 % (2.2 %)
**Hispanic White + Black + Other (HISP)**										
**All**	5,738	CN	3,549 (61.9 %)	16 (0.5 %)	3 (18.8 %)	2,460 (69.3 %)	74.6 (8.7)	42.1 % (23.7 %)	23.7 % (21.8 %)	32.9 % (28.6 %)
		AD	2,189 (38.1 %)	49 (2.2 %)	49 (100 %)	1,455 (66.5 %)	76.6 (9.0)	46.9 % (21.5 %)	29.6 % (21.3 %)	22.3 % (19.3 %)
***APOE* *22**	24	CN	15 (62.5 %)	0 (0.0 %)	-	9 (60.0 %)	79.4 (8.6)	45.2 % (20.0 %)	32.0 % (24.6 %)	21.6 % (19.5 %)
		AD	9 (37.5 %)	0 (0.0 %)	-	8 (88.9 %)	79.7 (7.0)	32.8 % (24.4 %)	52.8 % (31.7 %)	11.6 % (12.6 %)
***APOE* *23**	482	CN	330 (68.5 %)	2 (0.6 %)	0 (0.0 %)	236 (71.5 %)	76.6 (9.0)	42.0 % (23.8 %)	28.6 % (24.1 %)	28.0 % (26.7 %)
		AD	151 (31.5 %)	2 (1.3 %)	2 (100 %)	107 (70.4 %)	79.7 (9.7)	45.2 % (20.1 %)	35.1 % (22.8 %)	18.7 % (14.4 %)
***APOE* *33**	3,517	CN	2,376 (67.6%)	12 (0.5 %)	3 (25.0 %)	1,641 (69.1 %)	74.7 (8.7)	42.2 % (24.2 %)	21.8 % (21.2 %)	34.7 % (29.4 %)
		AD	1,141 (32.4 %)	24 (2.1 %)	24 (100 %)	751 (65.8 %)	77.4 (9.0)	47.8 % (21.8 %)	27.6 % (20.3 %)	23.4 % (20.6 %)
***APOE* *24**	101	CN	57 (56.4 %)	0 (0.0 %)	-	44 (77.2 %)	74.4 (7.9)	41.0 % (21.6 %)	35.9 % (24.7 %)	22.0 % (20.9 %)
		AD	44 (43.6 %)	0 (0.0 %)	-	26 (59.1 %)	80.0 (7.7)	41.4 % (18.4 %)	41.8 % (22.6 %)	15.5 % (10.1 %)
***APOE* *34**	1,426	CN	712 (49.9 %)	2 (0.3 %)	0 (0.0 %)	489 (68.7 %)	73.6 (8.3)	41.8 % (22.1 %)	26.2 % (21.5 %)	30.6 % (27.1 %)
		AD	714 (50.1 %)	20 (2.8 %)	20 (100 %)	481 (67.4 %)	75.3 (8.7)	46.5 % (21.8 %)	30.0 % (21.6 %)	22.1 % (19.2 %)
***APOE* *44**	188	CN	59 (31.4 %)	0 (0.0 %)	-	41 (69.5 %)	70.2 (7.8)	41.3 % (21.7 %)	28.4 % (22.7 %)	28.9 % (25.0 %)
		AD	129 (68.6 %)	3 (2.3 %)	3 (100 %)	82 (63.6 %)	71.5 (7.4)	46.0 % (19.9 %)	33.1 % (21.2 %)	20.1 % (14.0 %)

© 2023 Belloy ME et al. *JAMA Neurology.*

eTable 4. Overview of Participant Demographics Across the Cohort-Platform Technical Covariate

	NHW		NHB		HISP	
**Cohort-Platform ID**	**CN**	**AD**	**CN**	**AD**	**CN**	**AD**
ACT	1,522	558	68	26	4	1
ADC1	292	1,206	21	17	8	18
ADC2	72	537	0	0	0	0
ADC3	297	563	0	0	7	6
ADC4	269	325	0	1	1	4
ADC5	358	324	0	0	0	0
ADC6	204	317	0	0	0	0
ADC7	372	281	0	0	0	0
ADM_O	111	123	0	0	0	0
ADM_Q	62	111	0	0	0	0
ADNI_1	21	53	1	4	1	1
ADNI_DOD	78	NA	5	NA	4	NA
ADNI_OE	1	2	0	0	0	0
ADNI3	197	20	5	0	8	1
ADSP_WES_Baylor	968	727	0	0	2	50
ADSP_WES_Broad	1,015	399	0	1	0	1
ADSP_WES_CU_IGM	590	55	858	125	479	196
ADSP_WES_MGI	211	127	0	10	2	0
ADSP_WES_Otogenetics	8	67	0	0	0	2
ADSP_WES_UM_HIHG	2	10	444	473	2	1
ADSP_WES_UW_GenomeSciences	NA	1	NA	0	NA	0
ADSP_WES_WashU	185	322	0	0	4	40
ADSP_WGS_BAYLOR	74	41	0	0	473	532
ADSP_WGS_BROAD	426	548	0	2	110	49
ADSP_WGS_GENENTECH	2	10	0	0	0	0
ADSP_WGS_ILLUMINA	400	313	7	3	5	4
ADSP_WGS_MACROGEN	NA	1	NA	0	NA	0
ADSP_WGS_NYGC	298	850	2	16	2	6
ADSP_WGS_USUHS	1,644	1,902	1,139	626	917	150
ADSP_WGS_WASHU	32	42	479	445	62	15
CHOP	459	535	3	5	1	0
CIDR	0	0	0	0	888	821
GSK	750	664	0	0	0	0
HBTRC_ILL	45	85	0	0	0	0
HBTRC_PERL	76	151	0	0	0	0
IIDP_AA	0	0	702	56	0	0
IIDP_YOR	0	0	980	85	0	0
LATC	0	NA	9	NA	0	NA
LOAD	466	369	33	21	30	148
MARS	0	0	346	43	1	2
MAYO_1	953	684	3	8	0	0
MAYO_2	87	2	0	0	0	0
MIA	59	333	0	3	0	0
MIRAGE_370	36	21	0	0	0	1
MIRAGE_610	214	48	0	1	0	5
MTC	116	176	0	0	0	0
NS	14	33	0	0	0	0
OHSU	205	57	1	0	0	0
ROSMAP_1B	392	270	0	0	0	0
ROSMAP_1T	75	86	0	0	4	2
ROSMAP_2C	148	72	0	0	3	2
ROSMAP_3BU	172	63	4	5	28	18
TARCC	18	94	0	0	0	0
TARCC_full	409	488	17	23	503	112
TGEN	369	706	0	0	0	1
UPITT	819	1,086	2	1	0	0
UVM_A	600	145	0	0	0	0
UVM_B	322	200	0	0	0	0
UVM_C	109	288	3	5	0	0
WASHU	140	261	0	0	0	0
WASHU2	5	8	0	0	0	0
WHICAP	155	25	0	0	0	0
WU	134	178	2	6	0	0

The technical covariate represents the subdivision of publicly available array-based cohorts/studies across respective arrays, as well as the subdivision of ADSP WES/WGS across respective sequencing platforms.

© 2023 Belloy ME et al. *JAMA Neurology.*

eTable 5. Overview of Participant Demographics and Ascertainment Design Across Cohorts

		NHW		NHB		HISP	
**Cohort**	**Ascertainment design**	**CN**	**AD**	**CN**	**AD**	**CN**	**AD**
ACT	Community/Population	1,703	701	68	26	4	1
ADC	non-Community/Population	3,671	5,552	921	625	41	52
ADM	non-Community/Population	173	234	0	0	0	0
ADNI	non-Community/Population	630	584	29	14	24	17
ARIC	Community/Population	18	36	0	0	0	0
CCS	Community/Population	205	0	0	0	0	0
CHAP	Community/Population	201	25	0	0	0	0
CHS	Community/Population	530	216	0	0	2	1
CIDR	non-Community/Population	0	0	0	0	1,290	1,274
ERF	non-Community/Population	74	86	0	0	0	0
FHS	Community/Population	407	153	0	0	0	0
GAA	Community/Population	0	0	175	204	0	0
GDF	Community/Population	204	50	2	4	0	0
GSK	non-Community/Population	750	664	0	0	0	0
HBTRC	Community/Population	121	236	0	0	0	0
IIDP_AA	Community/Population	0	0	981	159	0	0
IIDP_YOR	Community/Population	0	0	980	85	0	0
KGAD	non-Community/Population	210	132	0	11	2	0
LATC	non-Community/Population	0	0	9	0	0	0
LOAD	non-Community/Population	700	749	35	28	30	151
MARS	Community/Population	0	0	370	66	1	3
MAYO	non-Community/Population	1,112	776	3	8	0	0
MIA	non-Community/Population	80	438	530	362	0	1
MIRAGE	non-Community/Population	250	396	34	91	0	55
MSBB	non-Community/Population	23	166	2	16	1	6
MTC	non-Community/Population	116	176	0	0	0	0
NCRD	non-Community/Population	0	317	0	1	0	0
NS	non-Community/Population	14	33	0	0	0	0
OHSU	non-Community/Population	205	57	1	0	0	0
PRHS	non-Community/Population	0	0	0	0	886	115
RAS	non-Community/Population	0	39	0	0	0	0
ROSMAP	Community/Population	991	887	22	19	37	22
RS	Community/Population	750	268	0	0	0	0
STEP	non-Community/Population	34	96	0	0	0	0
TARCC	non-Community/Population	427	591	17	23	503	112
TGEN	non-Community/Population	369	706	0	0	0	1
TOR	non-Community/Population	0	89	0	0	0	0
UCLA	non-Community/Population	13	3	0	0	1	0
UPITT	non-Community/Population	819	1,289	2	1	0	0
UPN	-	4	17	0	0	0	0
UVM	non-Community/Population	1,031	633	3	5	0	0
VAN	non-Community/Population	2	14	56	38	0	0
WASHU	non-Community/Population	279	447	2	6	0	0
WHICAP	Community/Population	942	107	892	219	727	378
	**non-Community/Population**	10,982	14,267	1,644	1,229	2,778	1,784
	**Community/Population**	6,072	2,679	3,490	782	771	405

Original cohorts in ADSP were identified from sample IDs and pooled with array-based samples, when contributed by respective, matching cohorts. Ascertainment design was annotated based on review of cohorts in a manner similar to Farrer et al. 1997, which used a trichotomous classification scheme based on one of the following recruitment settings: community/population (com), clinic/hospital (clin), or autopsy/brain bank (aut). For the current study, we pooled clin and aut samples into one group: non-community/population (non-com). This is consistent with the primary analyses presented in Farrer et al. 1997, Table 3, which has been a common reference in the field for cross-race/ethnicity differences of *APOE* genotype associations with AD risk (cf. eTable10-11 for related sensitivity analyses).

© 2023 Belloy ME et al. *JAMA Neurology.*

eTable 6. Case-Control Regression Results Across *APOE* Strata Corresponding to Figure 1a

	East-Asian (EAS)			Non-Hispanic Whites (NHW)			Non-Hispanic Blacks (NHB)			Hispanic (HISP)		
**Group/Model**	**No. carriers**	**OR (95% CI)**	**P-value**	**No. carriers**	**OR (95% CI)**	**P-value**	**No. carriers**	**OR (95% CI)**	**P-value**	**No. carriers**	**OR (95% CI)**	**P-value**
***APOE* across race/ethnicity**												
***APOE* genotype**												
APOE *22+23	1,230	0.97 [0.77, 1.23]	0.80	3,019	0.53 [0.48, 0.58]	**2.44E-40**	1,077	0.69 [0.57, 0.84]	**2.56E-04**	506	0.89 [0.72, 1.10]	0.29
APOE *33	15,904	Ref.	-	17,199	Ref.	-	3,095	Ref.	-	3,517	Ref.	-
APOE *24	157	2.96 [1.85, 4.73]	**6.05E-06**	741	2.23 [1.90, 2.62]	**6.39E-23**	334	1.63 [1.24, 2.13]	**4.06E-04**	101	1.11 [0.72, 1.72]	0.63
APOE *34	4,164	4.54 [3.99, 5.17]	**3.11E-115**	10,807	3.46 [3.27, 3.65]	**<1.0E-300**	2,233	2.18 [1.90, 2.49]	**9.82E-30**	1,426	1.90 [1.65, 2.18]	**8.39E-20**
APOE *44	397	26.13 [19.06, 35.82]	**1.81E-91**	2,255	13.04 [11.31, 15.04]	**1.16E-273**	406	6.49 [5.07, 8.31]	**1.10E-49**	188	3.62 [2.56, 5.11]	**2.55E-13**

© 2023 Belloy ME et al. *JAMA Neurology.*

eTable 7. Case-Control Regression Results Across *APOE* Dosages and Strata for Hispanic Individuals (HISP), Stratified Into Hispanic Whites (HW) and Hispanic Blacks (HB)

	Hispanic (HISP)					
	Whites (HW)			Blacks (HB)		
**Group/Model**	**No. carriers**	**OR (95% CI)**	**P-value**	**No. carriers**	**OR (95% CI)**	**P-value**
***APOE* across race/ethnicity**						
***APOE* *4 and *APOE* *2 dosage**						
APOE *2 dosage	120	1.21 [0.79, 1.88]	0.38	108	1.07 [0.67, 1.69]	0.78
APOE *33	791	Ref.	-	545	Ref.	-
APOE *4 dosage	566	2.07 [1.68, 2.57]	**1.79E-11**	479	2.08 [1.65, 2.62]	**5.67E-10**
***APOE* genotype**						
APOE *22	4	1.81 [0.17, 19.19]	0.62	4	2.09 [0.2, 22.29]	0.54
APOE *23	91	1.18 [0.74, 1.87]	0.49	77	0.99 [0.60, 1.64]	0.98
APOE *33	791	Ref.	-	545	Ref.	-
APOE *24	25	1.37 [0.57, 3.28]	0.48	27	1.04 [0.46, 2.34]	0.92
APOE *34	450	2.36 [1.80, 3.08]	**3.82E-10**	366	2.25 [1.67, 3.03]	**8.45E-08**
APOE *44	91	3.08 [1.78, 5.35]	**6.16E-05**	86	3.56 [1.97, 6.44]	**2.63E-05**

© 2023 Belloy ME et al. *JAMA Neurology.*

eTable 8. Sensitivity Case-Control Regression Analyses Corresponding to Table 1, Using Clinically Determined Phenotypes Only

	Non-Hispanic						Hispanic			Race/ethnicity effect		
	Whites (NHW)			Blacks (NHB)			Whites + Blacks + Other (HISP)			NHW vs. NHB	NHW vs. HISP	NHB vs. HISP
Group/Model	No. carriers	OR (95% CI)	P-value	No. carriers	OR (95% CI)	P-value	No. carriers	OR (95% CI)	P-value	P-value	P-value	P-value
***APOE* across race/ethnicity**												
***APOE* *4 and *APOE* *2 dosage**												
APOE *2 dosage	2,960	0.61 [0.55, 0.68]	**2.61E-19**	1,375	0.72 [0.59, 0.87]	**5.35E-04**	603	0.91 [0.74, 1.11]	0.33	0.15	**7.83E-04**	0.10
APOE *33	13,359	Ref.	-	3,027	Ref.	-	3,481	Ref.	-	-	-	-
APOE *4 dosage	9,522	3.27 [3.10, 3.45]	**<1.0E-300**	2,829	2.31 [2.08, 2.57]	**4.33E-55**	1,690	1.89 [1.68, 2.11]	**1.95E-27**	**7.51E-09**	**1.80E-16**	**0.010**
***APOE* genotype**												
APOE *22	102	0.53 [0.32, 0.89]	**0.017**	65	0.82 [0.40, 1.66]	0.58	24	1.01 [0.41, 2.50]	0.98	0.34	0.225	0.72
APOE *23	2,329	0.59 [0.53, 0.67]	**5.47E-19**	989	0.68 [0.55, 0.84]	**2.68E-04**	478	0.89 [0.71, 1.11]	0.29	0.27	**1.48E-03**	0.08
APOE *33	13,359	Ref.	-	3,027	Ref.	-	3,481	Ref.	-	-	-	-
APOE *24	529	1.96 [1.62, 2.37]	**4.02E-12**	321	1.65 [1.25, 2.17]	**4.19E-04**	101	1.12 [0.72, 1.74]	0.61	0.31	**0.022**	0.15
APOE *34	7,568	3.22 [3.02, 3.44]	**2.06E-278**	2,134	2.08 [1.82, 2.39]	**1.69E-25**	1,404	1.88 [1.64, 2.16]	**4.97E-19**	**2.04E-08**	**4.30E-12**	0.30
APOE *44	1,425	11.91 [10.20, 13.90]	**3.03E-216**	374	6.15 [4.78, 7.92]	**2.07E-45**	185	3.57 [2.53, 5.05]	**5.42E-13**	**1.20E-05**	**4.70E-10**	**0.013**
***APOE* across race/ethnicity & sex**												
***APOE* *4 and *APOE* *2 dosage**												
APOE *2 dosage, males	1,483	0.61 [0.53, 0.70]	**2.78E-12**	426	0.61 [0.42, 0.88]	**8.00E-03**	176	0.72 [0.49, 1.06]	0.092	0.54	0.86	0.55
APOE *33, males	5,312	Ref.	-	853	Ref.	-	1,113	Ref.	-	-	-	-
APOE *4 dosage, males	3,790	3.22 [2.96, 3.51]	**5.52E-161**	819	2.45 [1.99, 3.03]	**7.44E-17**	537	1.83 [1.49, 2.24]	**5.09E-09**	**0.018**	**4.63E-07**	0.050
APOE *2 dosage, females	1,790	0.56 [0.49, 0.65]	**1.53E-15**	949	0.77 [0.61, 0.95]	**0.018**	427	0.99 [0.78, 1.25]	0.90	**0.020**	**6.85E-05**	0.13
APOE *33, females	8,047	Ref.	-	2,174	Ref.	-	2,368	Ref.	-	-	-	-
APOE *4 dosage, females	2,960	3.32 [3.10, 3.56]	**1.94E-256**	2,010	2.27 [2.01, 2.57]	**1.27E-39**	1,153	1.93 [1.67, 2.22]	**4.56E-20**	**1.11E-07**	**7.14E-12**	0.080
APOE *2 dosage, sex effect (male ref.)	3,760	0.87 [0.72, 1.06]	0.17	1,375	1.10 [0.76, 1.58]	0.62	603	1.25 [0.83, 1.88]	0.29	0.28	0.12	0.64
APOE *4 dosage, sex effect (male ref.)	9,522	0.97 [0.88, 1.08]	0.60	2,829	0.92 [0.73, 1.17]	0.51	1,690	1.05 [0.83, 1.33]	0.69	0.69	0.57	0.46
***APOE* genotype**												
APOE *22, males	48	0.56 [0.26, 1.21]	0.14	21	0.70 [0.18, 2.65]	0.60	7	0.22 [0.02, 2.34]	0.21	0.78	0.45	0.40
APOE *23, males	930	0.68 [0.57, 0.81]	**2.67E-05**	309	0.56 [0.38, 0.84]	**5.52E-03**	138	0.77 [0.51, 1.15]	0.20	0.40	0.60	0.30
APOE *33, males	5,312	Ref.	-	853	Ref.	-	1,113	Ref.	-	-	-	-
APOE *24, males	192	1.99 [1.45, 2.73]	**2.01E-05**	96	1.95 [1.14, 3.35]	**0.015**	31	1.39 [0.63, 3.07]	0.42	0.95	0.41	0.49
APOE *34, males	2,984	3.03 [2.73, 3.36]	**7.50E-98**	623	2.11 [1.60, 2.77]	**8.99E-08**	444	1.72 [1.35, 2.20]	**1.61E-05**	**0.015**	**3.53E-05**	0.28
APOE *44, males	614	12.80 [9.95, 16.46]	**9.30E-88**	100	7.72 [4.55, 13.08]	**3.36E-14**	62	3.86 [2.07, 7.17]	**2.01E-05**	0.090	**4.44E-04**	0.10
APOE *22, females	54	0.53 [0.26, 1.09]	0.084	44	0.88 [0.38, 2.05]	0.77	17	1.57 [0.57, 4.34]	0.38	0.37	**3.68E-04**	0.39
APOE *23, females	1,399	0.54 [0.46, 0.63]	**9.62E-16**	680	0.73 [0.57, 0.93]	**1.22E-02**	340	0.93 [0.72, 1.22]	0.62	**0.037**	**0.031**	0.18
APOE *33, females	8,047	Ref.	-	2,174	Ref.	-	2,368	Ref.	-	-	-	-
APOE *24, females	337	1.96 [1.55, 2.49]	**3.06E-08**	225	1.56 [1.12, 2.16]	**8.08E-03**	70	1.04 [0.61, 1.76]	0.90	0.258	**0.031**	0.20
APOE *34, females	4,584	3.38 [3.11, 3.68]	**2.93E-182**	1,511	2.08 [1.77, 2.45]	**4.50E-19**	960	1.96 [1.65, 2.31]	**5.97E-15**	**1.50E-07**	**1.03E-08**	0.60
APOE *44, females	811	11.55 [9.46, 14.09]	**4.79E-128**	274	5.84 [4.37, 7.81]	**1.33E-32**	123	3.52 [2.31, 5.38]	**5.67E-09**	**1.51E-04**	**6.56E-07**	0.054
APOE*22, sex effect (male ref.)	102	1.05 [0.37, 2.97]	0.93	65	1.29 [0.27, 6.05]	0.75	24	8.24 [0.63, 108.00]	0.11	0.83	0.15	0.23
APOE*23, sex effect (male ref.)	2,329	0.83 [0.66, 1.05]	0.11	989	1.27 [0.79, 2.02]	0.32	478	1.21 [0.75, 1.96]	0.44	0.11	0.17	0.89
APOE*24, sex effect (male ref.)	529	0.97 [0.66, 1.44]	0.88	321	0.81 [0.45, 1.49]	0.50	101	0.76 [0.29, 1.94]	0.56	0.63	0.63	0.90
APOE*34, sex effect (male ref.)	7,568	1.05 [0.92, 1.19]	0.47	2,134	1.00 [0.74, 1.36]	0.99	1,404	1.14 [0.85, 1.54]	0.38	0.79	0.60	0.55
APOE*44, sex effect (male ref.)	1,425	0.76 [0.56, 1.04]	0.082	374	0.79 [0.45, 1.39]	0.41	185	0.92 [0.44, 1.92]	0.82	0.92	0.65	0.75

Samples with pathology data or pathology-verified diagnoses were excluded. Compared to case-control regression results presented in Table 1, the current table shows lost significant associations in orange-shaded cells. Note the loss of significant *APOE**2, *APOE**23, and *APOE**24 association differences across Non-Hispanic White (NHW) and Non-Hispanic Black (NHB), but the overall conserved pattern (more protective in NHW).

© 2023 Belloy ME et al. *JAMA Neurology.*

eTable 9. Sensitivity Case-Control Regression Analyses Corresponding to Table 1, Additionally Adjusting for Pathology Verification Status

	Non-Hispanic						Hispanic			Race/ethnicity effect		
	Whites (NHW)			Blacks (NHB)			Whites + Blacks + Other (HISP)			NHW vs. NHB	NHW vs. HISP	NHB vs. HISP
**Group/Model**	**No. carriers**	**OR (95% CI)**	**P-value**	**No. carriers**	**OR (95% CI)**	**P-value**	**No. carriers**	**OR (95% CI)**	**P-value**	**P-value**	**P-value**	**P-value**
***APOE* across race/ethnicity**												
***APOE*4 and APOE*2* dosage**												
APOE *2 dosage	3,760	0.56 [0.51, 0.61]	**2.66E-35**	1,411	0.72 [0.60, 0.87]	**5.80E-04**	607	0.90 [0.74, 1.10]	0.32	**1.39E-02**	**2.02E-05**	0.11
APOE *33	17,199	Ref.	-	3,095	Ref.	-	3,517	Ref.	-	-	-	-
APOE *4 dosage	13,803	3.48 [3.32, 3.66]	**<1.0E-300**	2,973	2.36 [2.13, 2.62]	**7.75E-59**	1,715	1.90 [1.69, 2.13]	**4.39E-28**	**2.74E-11**	**7.81E-22**	**5.69E-03**
***APOE* genotype**												
APOE *22	133	0.41 [0.26, 0.64]	**9.47E-05**	65	0.82 [0.40, 1.66]	0.58	24	1.01 [0.41, 2.50]	0.98	0.11	0.078	0.71
APOE *23	2,886	0.54 [0.49, 0.60]	**3.15E-33**	1,012	0.69 [0.56, 0.84]	**3.03E-04**	482	0.89 [0.71, 1.10]	0.28	**0.046**	**7.80E-05**	0.10
APOE *33	17,199	Ref.	-	3,095	Ref.	-	3,517	Ref.	-	-	-	-
APOE *24	741	2.18 [1.84, 2.57]	**5.15E-20**	334	1.59 [1.21, 2.09]	**9.09E-04**	101	1.12 [0.72, 1.74]	0.61	0.054	**5.49E-03**	0.19
APOE *34		3.46 [3.26, 3.66]	**<1.0E-300**	2,233	2.14 [1.87, 2.45]	**9.79E-28**	1,426	1.89 [1.65, 2.17]	**1.41E-19**	**2.35E-10**	**3.11E-15**	0.21
APOE *44	2,255	12.94 [11.20, 14.95]	**4.76E-265**	406	6.38 [4.97, 8.19]	**7.83E-48**	188	3.60 [2.55, 5.09]	**3.36E-13**	**1.57E-06**	**2.10E-11**	**8.60E-03**
***APOE* across race/ethnicity & sex**												
***APOE*4 and APOE*2* dosage**												
APOE *2 dosage, males	1,483	0.63 [0.55, 0.73]	**3.76E-10**	441	0.64 [0.45, 0.91]	**0.013**	177	0.73 [0.50, 1.07]	0.11	0.97	0.49	0.61
APOE *33, males	6,925	Ref.	-	878	Ref.	-	1,125	Ref.	-	-	-	-
APOE *4 dosage, males	5,718	3.48 [3.23, 3.76]	**7.16E-228**	865	2.55 [2.06, 3.14]	**2.26E-18**	552	1.86 [1.52, 2.27]	**1.96E-09**	**5.80E-03**	**1.09E-08**	**0.033**
APOE *2 dosage, females	2,277	0.51 [0.45, 0.57]	**1.05E-27**	970	0.76 [0.61, 0.95]	**0.015**	430	0.98 [0.77, 1.24]	0.85	**1.31E-03**	**1.37E-06**	0.13
APOE *33, females	10,274	Ref.	-	2,217	Ref.	-	2,392	Ref.	-	-	-	-
APOE *4 dosage, females	8,085	3.51 [3.3, 3.74]	**<1.0E-300**	2,108	2.31 [2.05, 2.61]	**6.16E-42**	1,163	1.94 [1.68, 2.23]	**2.12E-20**	**1.91E-09**	**2.66E-14**	0.060
APOE *2 dosage, sex effect (male ref.)	3,760	0.85 [0.72, 1.00]	0.055	1,411	1.05 [0.74, 1.50]	0.78	607	1.22 [0.81, 1.84]	0.33	0.29	0.10	0.59
APOE *4 dosage, sex effect (male ref.)	13,803	0.94 [0.86, 1.04]	0.24	2,973	0.91 [0.72, 1.15]	0.43	1,715	1.05 [0.82, 1.33]	0.71	0.77	0.43	0.41
***APOE* genotype**												
APOE *22, males	65	0.51 [0.27, 0.95]	**0.034**	21	0.69 [0.18, 2.62]	0.59	7	0.22 [0.02, 2.33]	0.21	0.68	0.49	0.40
APOE *23, males	1,148	0.62 [0.53, 0.72]	**1.65E-09**	321	0.59 [0.40, 0.88]	**8.80E-03**	139	0.78 [0.52, 1.17]	0.23	0.85	0.29	0.34
APOE *33, males	6,925	Ref.	-	878	Ref.	-	1,125	Ref.	-	-	-	-
APOE *24, males	270	2.26 [1.72, 2.98]	**6.44E-09**	99	1.93 [1.13, 3.28]	**0.015**	31	1.39 [0.63, 3.08]	0.41	0.60	0.26	0.50
APOE *34, males	4,448	3.33 [3.04, 3.64]	**4.62E-148**	657	2.23 [1.70, 2.93]	**6.70E-09**	456	1.75 [1.37, 2.24]	**8.01E-06**	**6.29E-03**	**1.58E-06**	0.19
APOE *44, males	1,000	14.23 [11.30, 17.93]	**2.45E-112**	109	8.09 [4.79, 13.67]	**5.76E-15**	65	4.03 [2.17, 7.48]	**9.84E-06**	0.053	**1.78E-04**	0.092
APOE *22, females	68	0.35 [0.18, 0.67]	**1.48E-03**	44	0.89 [0.38, 2.07]	0.78	17	1.58 [0.57, 4.35]	0.38	0.083	**0.014**	0.39
APOE *23, females	1,738	0.50 [0.44, 0.56]	**1.99E-26**	691	0.73 [0.57, 0.93]	**0.010**	343	0.93 [0.71, 1.20]	0.57	**5.67E-03**	**2.65E-05**	0.19
APOE *33, females	10,274	Ref.	-	2,217	Ref.	-	2,392	Ref.	-	-	-	-
APOE *24, females	471	2.14 [1.74, 2.65]	**1.28E-12**	235	1.49 [1.08, 2.06]	**0.016**	70	1.04 [0.61, 1.77]	0.88	0.064	**0.013**	0.26
APOE *34, females	6,359	3.59 [3.33, 3.87]	**2.62E-241**	1,576	2.11 [1.80, 2.48]	**3.62E-20**	970	1.97 [1.66, 2.33]	**2.59E-15**	**4.37E-09**	**1.69E-10**	0.55
APOE *44, females	1,255	12.29 [10.19, 14.83]	**8.46E-152**	297	6.06 [4.54, 8.08]	**2.41E-34**	123	3.52 [2.30, 5.37]	**5.80E-09**	**5.54E-05**	**1.17E-07**	**0.038**
APOE*22, sex effect (male ref.)	133	0.76 [0.31, 1.86]	0.55	65	1.30 [0.28, 6.12]	0.74	24	8.27 [0.63, 108.66]	0.11	0.56	0.086	0.23
APOE*23, sex effect (male ref.)	2,886	0.83 [0.68, 1.02]	0.073	1,012	1.19 [0.76, 1.86]	0.46	482	1.18 [0.73, 1.90]	0.50	0.16	0.19	0.98
APOE*24, sex effect (male ref.)	741	0.92 [0.65, 1.30]	0.64	334	0.79 [0.43, 1.43]	0.43	101	0.76 [0.29, 1.94]	0.56	0.66	0.70	0.94
APOE*34, sex effect (male ref.)	10,807	1.01 [0.90, 1.13]	0.93	2,233	0.96 [0.71, 1.31]	0.81	1,426	1.14 [0.85, 1.53]	0.38	0.80	0.43	0.43
APOE*44, sex effect (male ref.)	2,255	0.73 [0.55, 0.98]	**0.034**	406	0.80 [0.45, 1.40]	0.43	188	0.90 [0.43, 1.87]	0.77	0.80	0.62	0.80

The additional covariate marked “1” for samples with pathology data or pathology-verified diagnoses versus “0” for those without. Compared to case-control regression results presented in Table 1, the current table shows lost significant associations in orange-shaded cells. Note that overall the results are highly similar to those presented in Table 1.

© 2023 Belloy ME et al. *JAMA Neurology.*

eTable 10. Sensitivity Case-Control Regression Analyses Corresponding to Table 1 and eTable 10, Using Only Samples From Cohorts With a Given Ascertainment Design

Non-Com Samples

	Non-Hispanic						Hispanic			Race/ethnicity effect		
	Whites (NHW)			Blacks (NHB)			Whites + Blacks + Other (HISP)			NHW vs. NHB	NHW vs. HISP	NHB vs. HISP
Group/Model	No. carriers	OR (95% CI)	P-value	No. carriers	OR (95% CI)	P-value	No. carriers	OR (95% CI)	P-value	P-value	P-value	P-value
***APOE* across race/ethnicity**												
***APOE* *4 and *APOE* *2 dosage**												
APOE *2 dosage	2,415	0.53 [0.47, 0.59]	**1.08E-26**	509	0.59 [0.44, 0.79]	**3.37E-04**	434	0.86 [0.67, 1.1]	0.23	0.47	**4.07E-04**	0.051
APOE *33	11,806	Ref.	-	1,059	Ref.	-	2,792	Ref.	-	-	-	-
APOE *4 dosage	11,608	3.80 [3.59, 4.02]	**<1.0E-300**	1,450	3.01 [2.60, 3.48]	**3.84E-50**	1,416	2.04 [1.8, 2.32]	**5.73E-28**	**3.33E-03**	**2.62E-18**	**8.37E-05**
***APOE* genotype**												
APOE *22	75	0.30 [0.16, 0.55]	**1.04E-04**	21	0.64 [0.21, 1.96]	0.49	17	0.94 [0.32, 2.83]	0.92	0.23	0.071	0.63
APOE *23	1,760	0.53 [0.46, 0.60]	**5.55E-24**	343	0.55 [0.40, 0.76]	**2.79E-04**	337	0.84 [0.64, 1.10]	0.20	0.75	**1.70E-03**	**0.049**
APOE *33	11,806	Ref.	-	1,059	Ref.	-	2,792	Ref.	-	-	-	-
APOE *24	580	2.34 [1.93, 2.84]	**8.71E-18**	145	1.50 [1.03, 2.19]	**4.06E-04**	80	1.05 [0.64, 1.74]	0.84	**0.041**	**3.52E-03**	0.26
APOE *34	8,914	3.86 [3.61, 4.13]	**<1.0E-300**	1,053	2.80 [2.31, 3.39]	**9.82E-30**	1,164	2.04 [1.74, 2.38]	**6.15E-19**	**1.79E-03**	**2.12E-13**	**0.012**
APOE *44	2,114	14.52 [12.35, 17.08]	**6.37E-230**	252	10.06 [7.06, 14.33]	**1.10E-49**	172	4.13 [2.85, 5.98]	**6.87E-14**	0.064	**1.13E-09**	**6.62E-04**

Com Samples

	Non-Hispanic						Hispanic			Race/ethnicity effect		
	Whites (NHW)			Blacks (NHB)			Whites + Blacks + Other (HISP)			NHW vs. NHB	NHW vs. HISP	NHB vs. HISP
Group/Model	No. carriers	OR (95% CI)	P-value	No. carriers	OR (95% CI)	P-value	No. carriers	OR (95% CI)	P-value	P-value	P-value	P-value
***APOE* across race/ethnicity**												
***APOE* *4 and *APOE* *2 dosage**												
APOE *2 dosage	1,343	0.64 [0.55, 0.75]	**1.45E-08**	902	0.84 [0.66, 1.07]	0.15	173	1.02 [0.70, 1.48]	0.92	0.067	**0.025**	0.39
APOE *33	5,383	Ref.	-	2,036	Ref.	-	725	Ref.	-	-	-	-
APOE *4 dosage	2,185	2.05 [1.83, 2.29]	**5.14E-36**	1,523	1.72 [1.47, 2.01]	**1.46E-11**	299	1.21 [0.92, 1.60]	**1.72E-01**	0.078	**5.92E-04**	**0.031**
***APOE* genotype**												
APOE *22	58	0.73 [0.38, 1.40]	0.34	44	1.00 [0.40, 2.52]	0.99	7	1.10 [0.21, 5.77]	0.91	0.58	0.650	0.92
APOE *23	1,125	0.60 [0.51, 0.71]	**2.88E-09**	669	0.80 [0.61, 1.05]	0.10	145	1.01 [0.67, 1.53]	0.95	0.077	**0.021**	0.35
APOE *33	5,383	Ref.	-	2,036	Ref.	-	725	Ref.	-	-	-	-
APOE *24	160	1.57 [1.10, 2.25]	**0.014**	189	1.70 [1.14, 2.54]	**9.23E-03**	21	1.17 [0.47, 2.93]	0.74	0.77	0.56	0.46
APOE *34	1,884	2.04 [1.80, 2.32]	**4.48E-28**	1,180	1.55 [1.27, 1.90]	**1.93E-05**	262	1.24 [0.91, 1.69]	0.18	**0.024**	**3.62E-03**	0.23
APOE *44	141	4.59 [3.06, 6.89]	**1.62E-13**	154	3.56 [2.40, 5.29]	**2.83E-10**	16	1.25 [0.43, 3.65]	0.68	0.38	**0.026**	0.072

Upper Table) Samples from cohorts with a clinical/hospital or autopsy/brainbank ascertainment design (i.e. a non-community/population-based (non-com). **Lower Table)** Samples from cohorts with a community/population-based ascertainment design (com). All analyses were adjusted for pathology verification status as in eTable9 (which helps account for differences across clinical/hospital and autopsy/brainbank ascertainment designs). Compared to case-control regression results presented in Table 1, the current tables show new significant associations in green-shaded cells, and lost significant associations in orange-shaded cells. Overall, the general pattern of Table 1 and eTable9 is similar here, but note the less pronounced differences and loss of significances across NHW and NHB in non-com samples (upper table). Many significances were also lost in com analyses, but sample sizes (and thus power) were substantially decreased, so interpretation of race/ethnicity differences is better guided by judging effect size differences (the same holds true to a lesser extent for non-com samples).

© 2023 Belloy ME et al. *JAMA Neurology.*

eTable 11. Sensitivity Case-Control Regression Analyses Corresponding to Table 1 and eTable 10, Additionally Adjusting for Ascertainment Design

	Non-Hispanic						Hispanic			Race/ethnicity effect		
	Whites (NHW)			Blacks (NHB)			Whites + Blacks + Other (HISP)			NHW vs. NHB	NHW vs. HISP	NHB vs. HISP
**Group/Model**	**No. carriers**	**OR (95% CI)**	**P-value**	**No. carriers**	**OR (95% CI)**	**P-value**	**No. carriers**	**OR (95% CI)**	**P-value**	**P-value**	**P-value**	**P-value**
***APOE* across race/ethnicity**												
***APOE* *4 and *APOE* *2 dosage**												
APOE *2 dosage	3,758	0.57 [0.52, 0.62]	**3.60E-33**	1,411	0.72 [0.60, 0.87]	**5.27E-04**	607	0.90 [0.74, 1.11]	0.33	**0.022**	**3.35E-05**	0.10
APOE *33	17,189	Ref.	-	3,095	Ref.	-	3,517	Ref.	-	-	-	-
APOE *4 dosage	13,793	3.33 [3.17, 3.50]	**<1.0E-300**	2,973	2.36 [2.12, 2.61]	**2.24E-58**	1,715	1.89 [1.69, 2.12]	**1.08E-27**	**3.86E-09**	**4.82E-19**	**5.33E-03**
***APOE* genotype**												
APOE *22	133	0.44 [0.28, 0.69]	**3.28E-04**	65	0.81 [0.40, 1.65]	0.56	24	1.00 [0.41, 2.48]	0.99	0.15	0.11	0.72
APOE *23	2,885	0.55 [0.50, 0.61]	**1.22E-31**	1,012	0.68 [0.56, 0.84]	**2.83E-04**	482	0.89 [0.71, 1.11]	0.29	0.061	**1.07E-04**	0.090
APOE *33	17,189	Ref.	-	3,095	Ref.	-	3,517	Ref.	-	-	-	-
APOE *24	740	2.13 [1.80, 2.52]	**9.55E-19**	334	1.60 [1.22, 2.11]	**7.30E-04**	101	1.13 [0.73, 1.75]	0.59	0.081	**7.80E-03**	0.18
APOE *34	10,798	3.31 [3.12, 3.50]	**<1.0E-300**	2,233	2.13 [1.86, 2.45]	**1.98E-27**	1,426	1.89 [1.64, 2.17]	**2.22E-19**	**7.87E-09**	**2.38E-13**	0.22
APOE *44	2,255	12.24 [10.58, 14.15]	**2.73E-250**	406	6.44 [5.01, 8.27]	**4.07E-48**	188	3.58 [2.53, 5.06]	**4.73E-13**	**1.37E-05**	**1.30E-10**	**7.04E-03**

Ascertainment was classified as non-community/population-based (non-com) versus community/population-based (com) (cf. eTable5), using non-com ascertained samples as the reference. Analyses were additionally adjusted for pathology verification status as in eTable9. Compared to case-control regression results presented in Table 1, the current table shows lost significant associations in orange-shaded cells. Note the loss of significant *APOE**23 and *APOE**24 association differences across Non-Hispanic Whites (NHW) and Non-Hispanic Blacks (NHB), but the overall conserved pattern (more protective in NHW). Note that overall the results are highly similar to those presented in Table 1.

eTable 12. Sensitivity Case-Control Regression Analyses Corresponding to Table 1, Removing Samples in Which Race and Ethnicity Status Was Not Directly Provided From Cohort Demographic Files

	Non-Hispanic					
	Whites (NHW)			Blacks (NHB)		
**Group/Model**	**No. carriers**	**OR (95% CI)**	**P-value**	**No. carriers**	**OR (95% CI)**	**P-value**
***APOE* across race/ethnicity**						
APOE4 and *APOE***2 dosage**						
*APOE**2 dosage	3,096	0.55 [0.50, 0.61]	**2.28E-33**	1,234	0.72 [0.59, 0.86]	**4.84E-04**
*APOE**33	14,102	Ref.	-	2,720	Ref.	-
*APOE**4 dosage	11,146	3.30 [3.13, 3.48]	**<1.0E-300**	2,684	2.44 [2.20, 2.72]	**5.65E-61**
***APOE* genotype**						
*APOE**22	108	0.42 [0.26, 0.67]	**3.41E-04**	60	0.70 [0.33, 1.47]	0.35
*APOE**23	2,391	0.53 [0.48, 0.59]	**4.73E-32**	875	0.69 [0.56, 0.85]	**4.48E-04**
*APOE**33	14,102	Ref.	-	2,720	Ref.	-
*APOE**24	597	2.39 [2.00, 2.87]	**1.78E-21**	299	1.67 [1.26, 2.20]	**2.88E-04**
*APOE**34	8,828	3.35 [3.15, 3.56]	**<1.0E-300**	2,017	2.20 [1.92, 2.53]	**3.67E-29**
*APOE**44	1,721	10.84 [9.25, 12.71]	**1.72E-189**	368	6.93 [5.34, 9.00]	**5.96E-48**

Compared to case-control regression results presented in Table 1, the current table shows very similar findings for the respective race/ethnicity groups, except for potentially a slightly decreased *APOE**4 effect in NHW.

© 2023 Belloy ME et al. *JAMA Neurology.*

eTable 13. Survival Analyses Results, Through Cox Regression, Across *APOE* Dosages and Genotypes, and Additionally Stratified Across Sex, for Non-Hispanic White (NHW), Non-Hispanic Black (NHB), and Hispanic (HISP) Individuals

	Non-Hispanic						Hispanic			Race/ethnicity effect		
	Whites (NHW)			Blacks (NHB)			Whites + Blacks + Other (HISP)			NHW vs. NHB	NHW vs. HISP	NHB vs. HISP
Group/Model	No. carriers	HR (95% CI)	P-value	No. carriers	HR (95% CI)	P-value	No. carriers	HR (95% CI)	P-value	P-value	P-value	P-value
***APOE* across race/ethnicity**												
***APOE* *4 and *APOE* *2 dosage**												
APOE *2 dosage	3,516	0.57 [0.53, 0.61]	**1.63E-47**	1,362	0.73 [0.62, 0.86]	**2.07E-04**	558	0.78 [0.67, 0.91]	**1.23E-03**	**9.11E-03**	**2.94E-04**	0.56
APOE *33	16,121	Ref.	-	3,003	Ref.	-	3,372	Ref.	-	-	-	-
APOE *4 dosage	13,531	2.32 [2.27, 2.38]	**<1.0E-300**	2,902	2.23 [2.07, 2.40]	**1.24E-101**	1,673	1.78 [1.65, 1.92]	**9.19E-50**	0.31	**6.09E-11**	**2.71E-05**
***APOE* genotype**												
APOE *22	122	0.41 [0.28, 0.61]	**7.97E-06**	62	0.79 [0.42, 1.48]	0.46	22	0.89 [0.45, 1.79]	0.75	0.086	0.058	0.80
APOE *23	2,685	0.56 [0.52, 0.61]	**2.33E-44**	975	0.69 [0.57, 0.83]	**1.06E-04**	438	0.75 [0.62, 0.90]	**2.05E-03**	**0.043**	**4.88E-03**	0.55
APOE *33	16,121	Ref.	-	3,003	Ref.	-	3,372	Ref.	-	-	-	-
APOE *24	709	1.81 [1.63, 2.00]	**1.14E-30**	325	1.53 [1.23, 1.91]	**1.47E-04**	98	0.90 [0.66, 1.23]	0.51	0.19	**2.76E-05**	**6.08E-03**
APOE *34	10,581	2.50 [2.41, 2.59]	**<1.0E-300**	2,174	2.05 [1.84, 2.29]	**2.25E-39**	1,387	1.70 [1.54, 1.87]	**2.64E-27**	**6.35E-04**	**1.75E-13**	**0.011**
APOE *44	2,241	5.16 [4.88, 5.46]	**<1.0E-300**	403	5.52 [4.73, 6.44]	**4.76E-104**	188	3.39 [2.80, 4.10]	**4.05E-36**	0.43	**3.25E-05**	**9.80E-05**
***APOE* across race/ethnicity & sex**												
***APOE* *4 and *APOE* *2 dosage**												
APOE *2 dosage, males	1,410	0.61 [0.55, 0.69]	**1.58E-16**	425	0.69 [0.51, 0.94]	**0.018**	167	0.68 [0.50, 0.94]	**0.018**	0.47	0.54	0.95
APOE *33, males	6,588	Ref.	-	863	Ref.	-	1,089	Ref.	-	-	-	-
APOE *4 dosage, males	5,626	2.20 [2.12, 2.28]	**<1.0E-300**	848	2.05 [1.78, 2.35]	**7.73E-24**	543	1.66 [1.45, 1.89]	**9.16E-14**	0.34	**6.07E-05**	**0.031**
APOE *2 dosage, females	2,106	0.55 [0.49, 0.60]	**1.41E-31**	937	0.75 [0.62, 0.92]	**6.08E-03**	391	0.81 [0.68, 0.98]	**0.026**	**5.47E-03**	**2.09E-04**	0.59
APOE *33, females	9,533	Ref.	-	2,140	Ref.	-	2,283	Ref.	-	-	-	-
APOE *4 dosage, females	7,905	2.42 [2.34, 2.49]	**<1.0E-300**	2,054	2.32 [2.12, 2.53]	**1.63E-79**	1,130	1.87 [1.70, 2.05]	**5.29E-39**	0.39	**3.74E-07**	**9.69E-04**
APOE *2 dosage, sex effect (male ref.)	3,516	1.01 [0.89, 1.15]	0.87	1,362	1.04 [0.76, 1.41]	0.81	521	1.08 [0.91, 1.3]	0.37	0.87	0.53	0.81
APOE *4 dosage, sex effect (male ref.)	13,531	1.04 [0.99, 1.09]	0.14	2,902	1.10 [0.94, 1.29]	0.24	1,561	1.11 [0.95, 1.3]	0.19	0.47	0.41	0.95
***APOE* genotype**												
APOE *22, males	60	0.48 [0.28, 0.82]	**6.67E-03**	20	0.46 [0.15, 1.46]	0.19	7	0.45 [0.06, 3.24]	0.43	0.95	0.95	0.99
APOE *23, males	1,091	0.60 [0.53, 0.68]	**3.32E-15**	310	0.69 [0.49, 0.98]	**0.036**	129	0.69 [0.49, 0.96]	**0.027**	0.49	0.48	0.99
APOE *33, males	6,588	Ref.	-	863	Ref.	-	1,089	Ref.	-	-	-	-
APOE *24, males	259	1.60 [1.36, 1.89]	**2.61E-08**	95	1.62 [1.09, 2.42]	**0.018**	31	0.93 [0.57, 1.53]	0.78	0.95	**0.041**	0.09
APOE *34, males	4,371	2.25 [2.13, 2.37]	**1.12E-186**	645	1.87 [1.52, 2.29]	**1.65E-09**	447	1.54 [1.29, 1.83]	**1.12E-06**	0.086	**3.96E-05**	0.15
APOE *44, males	996	4.70 [4.32, 5.11]	**1.97E-282**	108	4.67 [3.49, 6.26]	**4.40E-25**	65	2.95 [2.13, 4.08]	**7.48E-11**	0.97	**6.60E-03**	**0.039**
APOE *22, females	62	0.38 [0.21, 0.67]	**8.50E-04**	42	1.11 [0.52, 2.36]	0.78	15	1.07 [0.51, 2.26]	0.85	**0.025**	**0.029**	0.95
APOE *23, females	1,594	0.54 [0.48, 0.60]	**5.29E-30**	665	0.70 [0.56, 0.88]	**1.77E-03**	309	0.76 [0.61, 0.96]	**0.018**	**0.036**	**5.36E-03**	0.59
APOE *33, females	9,533	Ref.	-	2,140	Ref.	-	2,283	Ref.	-	-	-	-
APOE *24, females	450	1.98 [1.75, 2.26]	**8.21E-26**	230	1.52 [1.16, 1.99]	**2.11E-03**	67	0.90 [0.60, 1.35]	0.60	0.080	**2.62E-04**	**0.033**
APOE *34, females	6,210	2.68 [2.56, 2.81]	**<1.0E-300**	1,529	2.14 [1.88, 2.44]	**2.45E-31**	940	1.81 [1.61, 2.04]	**8.25E-23**	**1.29E-03**	**1.22E-09**	0.057
APOE *44, females	1,245	5.53 [5.13, 5.96]	**<1.0E-300**	295	6.01 [5.00, 7.22]	**1.53E-81**	123	3.70 [2.92, 4.68]	**3.00E-27**	0.41	**1.50E-03**	**1.50E-03**
APOE*22, sex effect (male ref.)	122	0.85 [0.39, 1.86]	0.69	62	2.50 [0.64, 9.82]	0.19	21	2.13 [1.01, 4.48]	**0.046**	0.18	0.10	0.84
APOE*23, sex effect (male ref.)	2,685	0.94 [0.79, 1.10]	0.42	975	0.98 [0.65, 1.47]	0.91	404	1.08 [0.87, 1.34]	0.49	0.84	0.30	0.67
APOE*24, sex effect (male ref.)	709	1.19 [0.96, 1.46]	0.11	325	0.96 [0.60, 1.55]	0.88	96	0.90 [0.60, 1.35]	0.60	0.43	0.23	0.83
APOE*34, sex effect (male ref.)	10,581	1.12 [1.04, 1.20]	**1.94E-03**	2,174	1.14 [0.90, 1.44]	0.28	1,285	1.17 [1.05, 1.30]	**4.21E-03**	0.88	0.48	0.84
APOE*44, sex effect (male ref.)	2,241	1.01 [0.91, 1.12]	0.85	403	1.20 [0.86, 1.67]	0.28	180	1.15 [0.78, 1.70]	0.47	0.32	0.51	0.88

Compared to case-control regression results in Table 1, this table shows new significant associations in green-shaded cells, and lost significant associations in orange-shaded cells. Note more pronounced *APOE**23 effects in HISP individuals. Note loss of *APOE**4 dosage and *APOE**44 differences across NHW and NHB.

© 2023 Belloy ME et al. *JAMA Neurology.*

eTable 14. Case-Control Regression Results Across *APOE* Dosage and Strata, for Hispanic Individuals, Stratified Into Global Ancestry Quartiles

	Hispanic (HISP) across global European ancestry											
	EUR% < 25			EUR% > 25			EUR% > 50			EUR% > 75		
Group/Model	No. carriers	OR (95% CI)	P-value	No. carriers	OR (95% CI)	P-value	No. carriers	OR (95% CI)	P-value	No. carriers	OR (95% CI)	P-value
***APOE* across admixture**												
***APOE* *4 and *APOE* *2 dosage**												
APOE *2 dosage	133	0.86 [0.53, 1.39]	0.54	485	0.92 [0.74, 1.15]	0.46	240	0.75 [0.54, 1.04]	0.082	39	0.73 [0.33, 1.61]	0.44
APOE *33	725	Ref.	-	2,834	Ref.	-	1,571	Ref.	-	270	Ref.	-
APOE *4 dosage	309	2.14 [1.63, 2.81]	**3.84E-08**	1,430	1.85 [1.63, 2.10]	**6.10E-22**	695	1.92 [1.61, 2.29]	**5.45E-13**	120	2.65 [1.67, 4.22]	**3.77E-05**
***APOE* genotype**												
APOE *22	6	3.39 [0.51, 22.52]	0.21	19	0.80 [0.28, 2.29]	0.68	7	0.16 [0.01, 2.38]	0.18	2	0.38 [0.00, 32.5]	0.67
APOE *23	101	0.71 [0.41, 1.23]	0.22	389	0.93 [0.73, 1.17]	0.53	199	0.79 [0.56, 1.11]	0.17	33	0.75 [0.33, 1.73]	0.50
APOE *33	370	Ref.	-	2,834	Ref.	-	1,571	Ref.	-	270	Ref.	-
APOE *24	26	1.00 [0.41, 2.43]	1.00	77	1.11 [0.68, 1.84]	0.67	34	1.08 [0.50, 2.31]	0.85	4	0.11 [0.01, 1.95]	0.13
APOE *34	251	2.35 [1.69, 3.26]	**3.48E-07**	1,194	1.81 [1.56, 2.11]	**1.29E-14**	578	1.86 [1.50, 2.31]	**1.79E-08**	103	3.04 [1.73, 5.36]	**1.20E-04**
APOE *44	32	3.57 [1.60, 7.94]	**1.85E-03**	159	3.63 [2.48, 5.31]	**3.39E-11**	83	4.06 [2.36, 6.98]	**3.89E-07**	13	5.59 [1.42, 21.99]	**0.014**

	Hispanic (HISP) across global African ancestry											
	AFR% < 25			AFR% > 25			AFR% > 50			AFR% > 75		
Group/Model	No. carriers	OR (95% CI)	P-value	No. carriers	OR (95% CI)	P-value	No. carriers	OR (95% CI)	P-value	No. carriers	OR (95% CI)	P-value
***APOE* across admixture**												
***APOE* *4 and *APOE* *2 dosage**												
APOE *2 dosage	271	0.77 [0.56, 1.07]	0.12	347	0.96 [0.75, 1.25]	0.78	146	1.38 [0.93, 2.04]	0.11	35	2.14 [0.88, 5.21]	0.095
APOE *33	2,104	Ref.	-	1,462	Ref.	-	463	Ref.	-	82	Ref.	-
APOE *4 dosage	833	2.11 [1.78, 2.50]	**3.15E-18**	910	1.68 [1.44, 1.96]	**3.98E-11**	310	1.92 [1.46, 2.52]	**2.83E-06**	61	2.80 [1.40, 5.60]	**3.55E-03**
***APOE* genotype**												
APOE *22	9	0.48 [0.07, 3.31]	0.46	15	1.39 [0.48, 4.03]	0.55	9	2.03 [0.49, 8.39]	0.33	3	24.5 [1.32, 453.49]	**0.032**
APOE *23	227	0.78 [0.56, 1.11]	0.17	265	0.92 [0.69, 1.22]	0.57	105	1.39 [0.88, 2.20]	0.15	25	1.52 [0.49, 4.68]	0.47
APOE *33	2,104	Ref.	-	1,462	Ref.	-	463	Ref.	-	82	Ref.	-
APOE *24	35	1.01 [0.46, 2.24]	0.98	67	1.19 [0.71, 2.02]	0.51	32	1.21 [0.56, 2.61]	0.63	7	3.15 [0.53, 18.80]	0.21
APOE *34	719	2.11 [1.73, 2.57]	**1.86E-13**	731	1.64 [1.36, 1.99]	**3.11E-07**	238	1.82 [1.30, 2.55]	**4.67E-04**	46	3.62 [1.52, 8.62]	**3.74E-03**
APOE *44	79	4.48 [2.61, 7.70]	**5.33E-08**	112	3.03 [1.95, 4.72]	**8.22E-07**	40	4.42 [1.94, 10.04]	**3.92E-04**	8	5.63 [0.85, 37.31]	0.073

	Hispanic (HISP) across global Amerindian ancestry											
	AMR% < 25			AMR% > 25			AMR% > 50			AMR% > 75		
Group/Model	No. carriers	OR (95% CI)	P-value	No. carriers	OR (95% CI)	P-value	No. carriers	OR (95% CI)	P-value	No. carriers	OR (95% CI)	P-value
***APOE* across admixture**												
***APOE* *4 and *APOE* *2 dosage**												
APOE *2 dosage	436	0.90 [0.72, 1.13]	0.38	190	0.80 [0.53, 1.22]	0.30	55	0.68 [0.26, 1.76]	0.42	45	0.46 [0.14, 1.51]	0.20
APOE *33	2,071	Ref.	-	1,580	Ref.	-	559	Ref.	-	448	Ref.	-
APOE *4 dosage	1,171	1.84 [1.61, 2.11]	**1.26E-18**	604	1.81 [1.49, 2.20]	**3.30E-09**	172	2.26 [1.56, 3.27]	**1.48E-05**	138	2.10 [1.39, 3.17]	**4.19E-04**
***APOE* genotype**												
APOE *22	19	1.03 [0.39, 2.75]	0.95	5	0.96 [0.10, 8.96]	0.97	-	-	-	-	-	-
APOE *23	340	0.88 [0.69, 1.13]	0.33	158	0.78 [0.50, 1.22]	0.28	50	0.70 [0.26, 1.83]	0.46	41	0.47 [0.14, 1.58]	0.22
APOE *33	2,071	Ref.	-	1,580	Ref.	-	559	Ref.	-	448	Ref.	-
APOE *24	77	1.14 [0.71, 1.86]	0.58	27	0.96 [0.37, 2.51]	0.93	4	0.00 [0.00, Inf]	0.99	3	0.00 [0.00, Inf]	0.99
APOE *34	954	1.76 [1.49, 2.07]	**3.10E-11**	516	1.95 [1.54, 2.47]	**2.86E-08**	156	2.35 [1.53, 3.62]	**1.02E-04**	125	2.35 [1.45, 3.79]	**4.97E-04**
APOE *44	140	4.02 [2.65, 6.10]	**6.00E-11**	61	2.57 [1.41, 4.68]	**2.08E-03**	12	4.15 [1.25, 13.80]	**0.020**	10	2.69 [0.65, 11.11]	0.17

Note there was overall no clear pattern whereby global European, African, or Amerindian ancestry (when considering significant associations only) could explain why HISP showed less pronounced *APOE* effects on AD risk compared to NHW and NHB. One notable observation was an increasing effect of *APOE**44 on AD risk with increasing global EUR ancestry. However, even at >75% EUR ancestry, the *APOE**44 effect was only about half of what was observed in NHW (who are primarily >75% EUR (cf. eFigure2)), indicating an overall diminished *APOE**44 effect in HISP. A potentially interesting finding was that with higher AMR ancestry, the *APOE**44 effect was the most diminished (although based on few samples) and the *APOE**2 dosage effect became the most protective (although not significant).

© 2023 Belloy ME et al. *JAMA Neurology.*

Table 15. Case-Control Regression Results Across *APOE* Dosage and Strata, for Non-Hispanic Black Individuals, Stratified Into Global Ancestry Quartiles

	Non-Hispanic Black (NHB) across global European ancestry														
	EUR% < 25			EUR% > 25			EUR% > 50			EUR% > 75			EUR% > 90		
Group/Model	No. carriers	OR (95% CI)	P-value	No. carriers	OR (95% CI)	P-value	No. carriers	OR (95% CI)	P-value	No. carriers	OR (95% CI)	P-value	No. carriers	OR (95% CI)	P-value
***APOE* across admixture**															
***APOE**4 and *APOE**2 dosage**															
*APOE**2 dosage	868	0.73 [0.58, 0.91]	**6.43E-03**	241	0.67 [0.44, 1.01]	0.056	21	1.27 [0.31, 5.31]	0.74	0	-	-	0	-	-
*APOE**33	1,910	Ref.	-	546	Ref.	-	72	Ref.	-	0	Ref.	-	0	Ref.	-
*APOE**4 dosage	1,886	2.23 [1.96, 2.53]	**7.75E-34**	525	3.09 [2.44, 3.92]	**1.33E-20**	58	5.58 [2.43, 12.82]	**5.15E-05**	0	-	-	0	-	-
***APOE* genotype**															
*APOE**22	44	0.96 [0.42, 2.19]	0.92	11	0.00 [0.00, Inf]	0.98	1	0.00 [0.00, Inf]	1.00	0	-	-	0	-	-
*APOE**23	601	0.67 [0.52, 0.86]	**1.94E-03**	179	0.76 [0.49, 1.17]	0.21	13	2.84 [0.49, 16.53]	0.24	0	-	-	0	-	-
*APOE**33	1,910	Ref.	-	546	Ref.	-	72	Ref.	-	0	Ref.	-	0	Ref.	-
*APOE**24	223	1.85 [1.34, 2.54]	**1.75E-04**	51	1.62 [0.82, 3.18]	0.16	7	7.24 [1.11, 47.4]	**0.039**	0	-	-	0	-	-
*APOE**34	1,424	2.08 [1.76, 2.46]	**1.47E-17**	391	2.70 [1.99, 3.66]	**1.72E-10**	44	5.59 [2.09, 14.95]	**5.97E-04**	0	-	-	0	-	-
*APOE**44	239	5.54 [4.04, 7.59]	**1.79E-26**	83	12.86 [6.86, 24.09]	**1.56E-15**	7	38.96 [2.06, 737.98]	**0.015**	0	-	-	0	-	-

	Non-Hispanic Black (NHB) across global African ancestry														
	AFR% < 25			AFR% > 25			AFR% > 50			AFR% > 75			AFR% > 90		
Group/Model	No. carriers	OR (95% CI)	P-value	No. carriers	OR (95% CI)	P-value	No. carriers	OR (95% CI)	P-value	No. carriers	OR (95% CI)	P-value	No. carriers	OR (95% CI)	P-value
***APOE* across admixture**															
***APOE**4 and *APOE**2 dosage**															
*APOE**2 dosage	1	-	-	1,233	0.71 [0.59, 0.86]	**3.79E-04**	1,193	0.71 [0.58, 0.86]	**4.45E-04**	923	0.73 [0.58, 0.90]	**4.39E-03**	492	0.68 [0.49, 0.94]	**0.022**
*APOE**33	6	Ref.	-	2,714	Ref.	-	2,613	Ref.	-	2,041	Ref.	-	1,119	Ref.	-
*APOE**4 dosage	1	-	-	2,683	2.44 [2.19, 2.71]	**9.33E-61**	2,593	2.38 [2.13, 2.65]	**5.77E-56**	2,028	2.34 [2.06, 2.65]	**9.97E-41**	1,031	2.09 [1.73, 2.52]	**1.06E-14**
***APOE* genotype**															
*APOE**22	1	-	-	60	0.70 [0.33, 1.47]	0.34	58	0.74 [0.35, 1.58]	0.44	46	0.87 [0.38, 1.97]	0.74	31	0.85 [0.30, 2.46]	0.77
*APOE**23	1	-	-	874	0.68 [0.56, 0.84]	**3.41E-04**	847	0.68 [0.55, 0.83]	**2.82E-04**	644	0.67 [0.53, 0.86]	**1.75E-03**	329	0.60 [0.41, 0.89]	**9.99E-03**
*APOE**33	6	Ref.	-	2,714	Ref.	-	2,613	Ref.	-	2,041	Ref.	-	1,119	Ref.	-
*APOE**24	1	-	-	299	1.66 [1.26, 2.19]	**3.01E-04**	288	1.57 [1.18, 2.09]	**1.76E-03**	233	1.84 [1.35, 2.52]	**1.32E-04**	132	1.48 [0.94, 2.32]	0.088
*APOE**34	1	-	-	2,016	2.20 [1.92, 2.53]	**5.23E-29**	1,946	2.12 [1.84, 2.44]	**1.25E-25**	1,529	2.10 [1.79, 2.47]	**2.21E-19**	780	1.89 [1.48, 2.40]	**2.74E-07**
*APOE**44	0	-	-	368	6.92 [5.33, 8.98]	**7.25E-48**	359	6.69 [5.14, 8.71]	**1.80E-45**	266	6.42 [4.73, 8.70]	**5.37E-33**	119	5.17 [3.28, 8.16]	**1.72E-12**

Note (when considering significant associations only) there was a pattern for increasing global European, or decreasing global African, ancestry to associate with increased effect estimates for *APOE**4 (orange-shaded cells). Compared to eTable14, the global African ancestry proportion >90% was added since sample sizes were permissive to do so and we believed it was of interest to better assess the role of global African ancestry on *APOE* genotype associations with AD risk.

© 2023 Belloy ME et al. *JAMA Neurology.*

eTable 16. Sensitivity Case-Control Regression Analyses Mirroring Table 1, Considering Stratifications Across Global Population Ancestry Proportion Greater Than 75%

	Global Population Ancestry Proportion >75%									Race/ethnicity effect		
	European (EUR)			African (AFR)			Amerindian (AMR)			EUR vs. AFR	EUR vs. AMR	EUR vs. AFR
Group/Model	No. carriers	OR (95% CI)	P-value	No. carriers	OR (95% CI)	P-value	No. carriers	OR (95% CI)	P-value	P-value	P-value	P-value
***APOE* across race/ethnicity**												
***APOE* *4 and *APOE* *2 dosage**												
APOE *2 dosage	3,782	0.55 [0.51, 0.60]	**3.45E-39**	1,083	0.77 [0.62, 0.94]	**0.012**	44	0.48 [0.15, 1.59]	0.23	**4.00E-03**	0.83	0.45
APOE *33	17,383	Ref.	-	2,355	Ref.	-	447	Ref.	-	-	-	-
APOE *4 dosage	13,866	3.45 [3.29, 3.62]	**< 1.0E-300**	2,287	2.29 [2.04, 2.58]	**1.12E-42**	133	2.02 [1.32, 3.10]	**1.12E-03**	**3.13E-10**	**0.014**	0.58
***APOE* genotype**												
APOE *22	135	0.40 [0.26, 0.62]	**3.94E-05**	54	1.14 [0.56, 2.34]	0.72	1	0.00 [0.00, Inf]	0.99	**0.015**	0.99	0.99
APOE *23	2,906	0.54 [0.49, 0.59]	**4.64E-37**	765	0.69 [0.55, 0.88]	**2.10E-03**	40	0.50 [0.15, 1.68]	0.26	**0.048**	0.90	0.60
APOE *33	17,383	Ref.	-	2,355	Ref.	-	447	Ref.	-	-	-	-
APOE *24	741	2.22 [1.89, 2.60]	**1.13E-22**	264	1.78 [1.32, 2.40]	**1.78E-04**	3	0.00 [0.00, Inf]	0.99	**0.203**	0.99	0.99
APOE *34	10,867	3.43 [3.25, 3.63]	**< 1.0E-300**	1,727	2.10 [1.80, 2.45]	**5.85E-21**	121	2.15 [1.31, 3.51]	**2.36E-03**	**4.11E-09**	0.063	0.93
APOE *44	2,258	12.84 [11.15, 14.80]	**3.00E-273**	296	5.92 [4.45, 7.88]	**3.03E-34**	9	3.05 [0.71, 13.05]	0.13	**1.96E-06**	0.054	0.38

© 2023 Belloy ME et al. *JAMA Neurology.*

eReferences

1. Kunkle BW, Grenier-Boley B, Sims R, et al. Genetic meta-analysis of diagnosed Alzheimer's disease identifies new risk loci and implicates Aβ, tau, immunity and lipid processing. *Nat Genet*. 2019;51(3):414-430. doi:10.1038/s41588-019-0358-2

2. Bis JC, Jian X, Chen BWK, et al. Whole exome sequencing study identifies novel rare and common Alzheimer's-Associated variants involved in immune response and transcriptional regulation. *Mol Psychiatry*. 2020;25:1859-1875. doi:10.1038/s41380-018-0112-7

3. Beecham GW, Bis JC, Martin ER, et al. The Alzheimer's disease sequencing project: Study design and sample selection. *Neurol Genet*. 2017;3:e194. doi:10.1212/NXG.0000000000000194

4. Wang M, Beckmann ND, Roussos P, et al. The Mount Sinai cohort of large-scale genomic, transcriptomic and proteomic data in Alzheimer's disease. *Sci Data*. 2018;5:180185. doi:10.1038/sdata.2018.185

5. De Jager PL, Ma Y, McCabe C, et al. A multi-omic atlas of the human frontal cortex for aging and Alzheimer's disease research. *Sci Data*. 2018;5:180142. doi:10.1038/sdata.2018.142

6. Crane PK, Foroud T, Montine TJ, Larson EB. Alzheimer's Disease Sequencing Project Discovery and Replication criteria for cases and controls: data from a community-based prospective cohort study with autopsy follow-up. *Alzheimers Dement*. 2017;13(12):1410-1413. doi:10.1016/j.jalz.2017.09.010.

7. Mckhann G, Drachman D, Folstein M, Katzman R, Price D, Mckhann G. Clinical Diagnosis of Alzheimer's Disease: Report of the NINCDS-ADRDA Work Group Under the Auspices of Derpartment of Health and Human Services Task Force on Alzheimer's Disease. *Neurology*. 1984;34:27-28. doi:10.1212/01.wnl.0000400650.92875.cf

8. Bell CC. DSM-IV: Diagnostic and Statistical Manual of Mental Disorders. *JAMA*. 1994;272(10):828-829.

9. American Psychiatric Association. Diagnostic and Statistical Manual of Mental Disorders 5th edn. *Am Psychiatr Assoc*. Published online 2013.

10. American Psychiatric Association. Diagnostic and Statistical Manual of Mental Disorders 4th edn. *Am Psychiatr Assoc*. Published online 1994.

11. Hughes CP, Berg L, Danziger WL, Coben LA, Martin RL. A new clinical scale for the staging of dementia. *Br J Psychiatry*. 1982;140(6):566-572. doi:10.1192/bjp.140.6.566

12. Braak H, Braak E. Neuropathological stageing of Alzheimer-related changes. *Acta Neuropathol*. 1991;82:239-259. doi:10.1109/ICINIS.2015.10

13. Mirra SS, Heyman A, McKeel D, et al. The Consortium to Establish a Registry for Alzheimer's Disease (CERAD). Part II. Standardization of the neuropathologic assessement of Alzheimer's disease. *Neurology*. 1991;41:479-486. doi:10.1017/CBO9781107415324.004

14. Hyman BT, Trojanowski JQ. Editorial on Consensus Recommendations for the Postmortem Diagnosis of Alzheimer Disease from the National Institute on Aging and the Reagan Institute

© 2023 Belloy ME et al. *JAMA Neurology.*

Working Group on Diagnositic Criteria for the Neuropathological Assessment of Alzheimr Disease. *J Neuropathol Exp Neurol*. 1997;56(10):1095-1097.

15. Folstein MF, Folstein SE, McHugh PR. “Mini-Mental State” A Practical Method for Grading the Cognitive State of Patients for the Clinician. *J Psychiatr Res*. 1975;12:189-198. doi:10.3744/snak.2003.40.2.021

16. Roccaforte WH, Burke WJ, Bayer BL, Wengel SP. Validation of a Telephone Version of the Mini-Mental State Examination. *J Am Geriatr Soc*. 1992;40:697-702. https://seirei-univ.repo.nii.ac.jp/?action=repository_action_common_download&item_id=821&item_no=1&attribute_id=20&file_no=1

17. McKhann GM, Knopman DS, Chertkow H, et al. The diagnosis of dementia due to Alzheimer’s disease: Recommendations from the National Institute on Aging- Alzheimer’s Association workgroups on diagnostic guidelines for Alzheimer’s disease. *Alzheimers Dement*. 2011;7(3):263-269. doi:10.1016/j.jalz.2011.03.005.

18. Albert MS, DeKosky ST, Dickson D, et al. The diagnosis of mild cognitive impairment due to Alzheimer’s disease: Recommendations from the National Institute on Aging- Alzheimer’s Association workgroups on diagnostic guidelines for Alzheimer’s disease. *Alzheimers Dement*. 2011;7(3):270-279. doi:10.1016/j.jalz.2011.03.008.

19. Hyman BT, Phelps CH, Beach TG, et al. National Institute on Aging – Alzheimer’s Association guidelines for the neuropathologic assessment of Alzheimer’s disease. *Alzheimer’s Dement*. 2012;8(1):1-13. doi:10.1016/j.jalz.2011.10.007

20. Morris JC, Weintraub S, Chui HC, et al. The Uniform Data Set (UDS): Clinical and cognitive variables and descriptive data from Alzheimer disease centers. *Alzheimer Dis Assoc Disord*. 2006;20(4):210-216. doi:10.1097/01.wad.0000213865.09806.92

21. Weintraub S, Salmon D, Mercaldo N, et al. The Alzheimer’s Disease Centers’ Uniform Data Set (UDS) The Neuropsychologic Test Battery. *Alzheimer Dis Assoc Disord*. 2009;23(2):91-101. https://www.alz.washington.edu

22. Beekly DL, Ramos EM, Lee WW, et al. The National Alzheimer’s Coordinating Center (NACC) Database: The Uniform Data Set. *Alzheimer Dis Assoc Disord*. 2007;21(3):249-258. doi:10.1097/WAD.0b013e318142774e

23. Besser LM, Kukull WA, Teylan MA, et al. The revised national Alzheimer’s coordinating center’s neuropathology form-available data and new analyses. *J Neuropathol Exp Neurol*. 2018;77(8):717-726. doi:10.1093/jnen/nly049

24. Besser L, Kukull W, Knopman DS, et al. Version 3 of the national Alzheimer’s coordinating center’s uniform data set. *Alzheimer Dis Assoc Disord*. 2018;32(4):351-358. doi:10.1097/WAD.0000000000000279

25. Bennett DA, Schneider JA, Buchman AS, De Leon CM, Bienias JL, Wilson RS. The rush memory and aging project: Study design and baseline characteristics of the study cohort. *Neuroepidemiology*. 2005;25(4):163-175. doi:10.1159/000087446

26. Bennett DA, Wilson RS, Schneider JA, et al. Natural history of mild cognitive impairment in older

persons. *Neurology*. 2002;59(2):198-205. doi:10.1212/WNL.59.2.198

27. Schneider JA, Arvanitakis Z, Bang W, Bennett DA. Mixed brain pathologies account for most dementia cases in community-dwelling older persons. *Neurology*. 2007;69(24):2197-2204. doi:10.1212/01.wnl.0000271090.28148.24

28. Hinrichs AS, Karolchik D, Baertsch R, et al. The UCSC Genome Browser Database: update 2006. *Nucleic Acids Res*. 2006;34(Database issue):590-598. doi:10.1093/nar/gkj144

29. Deelen P, Bonder MJ, Van Der Velde KJ, et al. Genotype harmonizer: Automatic strand alignment and format conversion for genotype data integration. *BMC Res Notes*. 2014;7:901. doi:10.1186/1756-0500-7-901

30. Franke L, de Kovel CGF, Aulchenko YS, et al. Detection, Imputation, and Association Analysis of Small Deletions and Null Alleles on Oligonucleotide Arrays. *Am J Hum Genet*. 2008;82(6):1316-1333. doi:10.1016/j.ajhg.2008.05.008

31. Eichler EE, Nickerson DA, Altshuler D, et al. Completing the map of human genetic variation. *Nature*. 2007;447(7141):161-165. doi:10.1038/447161a

32. Karczewski KJ, Francioli LC, Tiao G, et al. The mutational constraint spectrum quantified from variation in 141,456 humans. *Nature*. 2020;581(May). doi:10.1038/s41586-020-2308-7

33. Chen CY, Pollack S, Hunter DJ, Hirschhorn JN, Kraft P, Price AL. Improved ancestry inference using weights from external reference panels. *Bioinformatics*. 2013;29(11):1399-1406. doi:10.1093/bioinformatics/btt144

34. Auton A, Abecasis GR, Altshuler DM, et al. A global reference for human genetic variation. *Nature*. 2015;526(7571):68-74. doi:10.1038/nature15393

35. Conomos MP, Miller MB, Thornton TA. Robust inference of population structure for ancestry prediction and correction of stratification in the presence of relatedness. *Genet Epidemiol*. 2015;39(4):276-293. doi:10.1002/gepi.21896

36. Zhu C, Farrell J, Kuzma A, Schellenberg G, Farrer L. Precision genotyping of APOE from whole genome sequencing. In: *American Society of Human Genetics Congress*. ; 2019.

37. Belloy ME, Eger SJ, Le Guen Y, et al. Challenges at the APOE locus: a robust quality control approach for accurate APOE genotyping. *Alz Res Ther*. 202; 14(22). doi: 10.1186/s13195-022-00962-4

38. Taliun D, Harris DN, Kessler MD, *et al.* Sequencing of 53,831 diverse genomes from the NHLBI TOPMed Program. *Nature*. 2021;590:290-299. doi:10.1038/s41586-021-03205-y

39. Das S, Forer L, Schönherr S, *et al.* Next-generation genotype imputation service and methods. *Nat Genet*. 2016;48:1284–1287. doi:10.1038/ng.3656

40. Le Guen Y, Belloy M, Napolioni V, *et al.* A novel age-informed approach for genetic association analysis in Alzheimer's disease. *Alz Res Ther.* 2021;13(27). doi:10.1186/s13195-021-00808-5

41. Wattmo C, Londos E, Minthon L. Risk factors that affect life expectancy in alzheimer's disease: A 15-year follow-up. *Dement Geriatr Cogn Disord*. 2014;38(5-6):286-299. doi:10.1159/000362926

© 2023 Belloy ME et al. *JAMA Neurology.*

42. Kuzma A, Valladares O, Cweibel R, et al. NIAGADS: The NIA Genetics of Alzheimer's Disease Data Storage Site. *Alzheimer's Dement*. 2016;12(11):1200-1203. doi:10.1016/j.jalz.2016.08.018

© 2023 Belloy ME et al. *JAMA Neurology.*
