# Supplementary material for: APOE Genotype and Alzheimer Disease Risk Across Age, Sex, and Population Ancestry
Source: JAMA Neurol. 2023 Nov 6;80(12):1284–94. doi: 10.1001/jamaneurol.2023.3599 (PMC10628838; doi:10.1001/jamaneurol.2023.3599)
Supplement: Supplement 2. — Data Sharing Statement [file jamaneurol-e233599-s002.pdf]

Data Sharing Statement

Belloy. APOE Genotype and Alzheimer Disease Risk Across Age, Sex, and Population Ancestry. *JAMA Neurol*. Published November 06, 2023. doi:10.1001/jamaneurol.2023.3599

Data
Data available: No

Additional Information
Explanation for why data not available: All data used in the manuscript are “de-identified, off-the-shelf” data publicly available upon application to the appropriate public repositories, so it's not up to us to disseminate them. Our study details exactly which datasets where used and from which repository so that other researchers can apply for them.
